# Supplementary material for: Genetic diversity and population structure of the endangered basal angiosperm Brasenia schreberi (Cabombaceae) in China
Source: PeerJ. 2018 Jul 13;6:e5296. doi: 10.7717/peerj.5296 (PMC6047506; doi:10.7717/peerj.5296)
Supplement: Supplemental Information 1 [file peerj-06-5296-s001.docx]

|  | HZXH | XGLHT | CQSZ | HZTJH | NXQZS | SZDSZ | SZLTS | YNGLG | YNTC | MSGH | YTLHS | LBMH | CLHL | GDSYD | QYBSZ | QYSYH | SCHSY | NDXFS | ZYFS | LCFBS | TWYL |
| --- | --- | --- | --- | --- | --- | --- | --- | --- | --- | --- | --- | --- | --- | --- | --- | --- | --- | --- | --- | --- | --- |
| HZXH | - | 657 | 1130 | 14 | 802 | 105 | 104 | 2111 | 2182 | 907 | 385 | 1605 | 738 | 757 | 286 | 301 | 227 | 344 | 295 | 1108 | 623 |
| XGLHT | 0.459 | - | 812 | 645 | 147 | 742 | 741 | 1671 | 1681 | 261 | 278 | 1174 | 188 | 133 | 423 | 427 | 437 | 407 | 570 | 797 | 640 |
| CQSZ | 0.512 | 0.437 | - | 1116 | 814 | 1147 | 1147 | 987 | 1091 | 719 | 863 | 493 | 626 | 707 | 1078 | 1097 | 1025 | 1104 | 848 | 23 | 1424 |
| HZTJH | 0.575 | 0.209 | 0.230 | - | 791 | 110 | 108 | 2097 | 2167 | 895 | 372 | 1951 | 724 | 745 | 280 | 296 | 218 | 339 | 282 | 1094 | 624 |
| NXQZS | 0.664 | 1.738 | 0.760 | 0.261 | - | 889 | 888 | 1606 | 1597 | 143 | 424 | 1122 | 212 | 120 | 560 | 561 | 581 | 536 | 709 | 803 | 718 |
| SZDSZ | 1.629 | 0.421 | 0.678 | 0.385 | 0.629 | - | 2 | 2133 | 2216 | 986 | 466 | 1632 | 807 | 835 | 390 | 406 | 324 | 449 | 299 | 1125 | 721 |
| SZLTS | 5.460 | 0.418 | 0.449 | 0.449 | 0.538 | 1.227 | - | 2133 | 2215 | 985 | 464 | 1631 | 806 | 834 | 388 | 404 | 323 | 447 | 299 | 1125 | 719 |
| YNGLG | 0.443 | 0.650 | 0.564 | 0.206 | 1.294 | 0.437 | 0.363 | - | 253 | 1468 | 1806 | 508 | 1491 | 1540 | 2022 | 2037 | 1983 | 2036 | 1834 | 1009 | 2311 |
| YNTC | 0.497 | 0.454 | 1.325 | 0.249 | 0.798 | 0.544 | 0.428 | 0.563 | - | 1454 | 1850 | 598 | 1512 | 1548 | 2061 | 2074 | 2032 | 2067 | 1920 | 1113 | 2313 |
| MSGH | 0.408 | 0.192 | 0.225 | 0.223 | 0.278 | 0.365 | 0.341 | 0.193 | 0.256 | - | 522 | 989 | 208 | 151 | 684 | 688 | 694 | 667 | 774 | 711 | 861 |
| YTLHS | 0.576 | 0.359 | 0.485 | 0.403 | 0.548 | 0.688 | 0.455 | 0.452 | 0.675 | 0.383 | - | 1299 | 360 | 373 | 217 | 234 | 185 | 243 | 316 | 843 | 588 |
| LBMH | 0.498 | 0.441 | 1.267 | 0.331 | 0.694 | 0.754 | 0.408 | 0.596 | 1.239 | 0.324 | 1.393 | - | 990 | 1045 | 1515 | 1530 | 1475 | 1530 | 1333 | 516 | 1813 |
| CLHL | 0.449 | 0.358 | 0.503 | 0.282 | 0.560 | 0.489 | 0.351 | 0.436 | 0.770 | 0.287 | 1.872 | 1.365 | - | 92 | 555 | 565 | 543 | 556 | 574 | 613 | 824 |
| GDSYD | 0.546 | 0.230 | 0.328 | 0.322 | 0.323 | 0.579 | 0.412 | 0.278 | 0.398 | 0.295 | 0.684 | 0.549 | 0.614 | - | 545 | 552 | 548 | 535 | 626 | 695 | 770 |
| QYBSZ | 0.195 | 0.147 | 0.222 | 0.187 | 0.295 | 0.196 | 0.160 | 0.257 | 0.247 | 0.150 | 0.274 | 0.307 | 0.290 | 0.197 | - | 24 | 90 | 64 | 401 | 1058 | 412 |
| QYSYH | 0.230 | 0.235 | 0.246 | 0.221 | 0.334 | 0.213 | 0.182 | 0.511 | 0.273 | 0.132 | 0.310 | 0.374 | 0.332 | 0.225 | 0.459 | - | 113 | 44 | 425 | 1077 | 388 |
| SCHSY | 0.276 | 0.198 | 0.354 | 0.316 | 0.307 | 0.258 | 0.218 | 0.307 | 0.326 | 0.157 | 0.378 | 0.489 | 0.390 | 0.292 | 0.432 | 0.528 | - | 152 | 312 | 1004 | 500 |
| NDXFS | 0.341 | 0.194 | 0.404 | 0.365 | 0.340 | 0.360 | 0.263 | 0.327 | 0.297 | 0.193 | 0.368 | 0.460 | 0.343 | 0.349 | 0.579 | 0.443 | 1.039 | - | 461 | 1085 | 358 |
| ZYFS | 0.328 | 0.401 | 0.801 | 0.152 | 0.588 | 0.408 | 0.257 | 0.789 | 0.374 | 0.155 | 0.362 | 0.659 | 0.381 | 0.245 | 0.168 | 0.294 | 0.235 | 0.305 | - | 826 | 812 |
| LCFBS | 0.320 | 0.235 | 1.411 | 0.163 | 0.422 | 0.487 | 0.259 | 0.373 | 0.394 | 0.169 | 0.446 | 1.107 | 0.405 | 0.242 | 0.153 | 0.172 | 0.303 | 0.332 | 0.524 | - | 1407 |

**Table S1**. Pairwise Nm values (below the diagonal) and geographic distances (above the diagonal) among the 21 sampled populations of *B. schreberi.*
