# Supplementary material for: Genetic diversity and population structure of the endangered basal angiosperm Brasenia schreberi (Cabombaceae) in China
Source: PeerJ. 2018 Jul 13;6:e5296. doi: 10.7717/peerj.5296 (PMC6047506; doi:10.7717/peerj.5296)
Supplement: Supplemental Information 2 — */ **/ *** Significant difference (P < 0.1/ 0.05/ 0.001). [file peerj-06-5296-s002.docx]

**Table S2.** Pairwise *F*st values (below the diagonal) and Nei’s genetic distances (above the diagonal) among the 21 sampled populations of *B. schreberi*

|  | HZXH | XGLHT | CQSZ | HZTJH | NXQZS | SZDSZ | SZLTS | YNGLG | YNTC | MSGH | YTLHS | LBMH | CLHL | GDSYD | QYBSZ | QYSYH | SCHSY | NDXFS | ZYFS | LCFBS | TWYL |
| --- | --- | --- | --- | --- | --- | --- | --- | --- | --- | --- | --- | --- | --- | --- | --- | --- | --- | --- | --- | --- | --- |
| HZXH | - | 0.207 | 0.304 | 0.135 | 0.199 | 0.125 | 0.068 | 0.205 | 0.301 | 0.278 | 0.259 | 0.323 | 0.280 | 0.234 | 0.343 | 0.304 | 0.288 | 0.268 | 0.284 | 0.294 | 0.187 |
| XGLHT | 0.353^***^ | - | 0.250 | 0.315 | 0.056 | 0.251 | 0.198 | 0.114 | 0.203 | 0.392 | 0.338 | 0.271 | 0.268 | 0.362 | 0.348 | 0.251 | 0.328 | 0.368 | 0.218 | 0.299 | 0.218 |
| CQSZ | 0.328^***^ | 0.364^***^ | - | 0.405 | 0.210 | 0.245 | 0.294 | 0.206 | 0.125 | 0.459 | 0.354 | 0.143 | 0.293 | 0.365 | 0.330 | 0.332 | 0.269 | 0.262 | 0.151 | 0.100 | 0.326 |
| HZTJH | 0.303^***^ | 0.545^***^ | 0.521^***^ | - | 0.307 | 0.241 | 0.161 | 0.331 | 0.397 | 0.333 | 0.264 | 0.341 | 0.322 | 0.272 | 0.285 | 0.263 | 0.225 | 0.203 | 0.390 | 0.361 | 0.318 |
| NXQZS | 0.273^***^ | 0.126^**^ | 0.247^***^ | 0.489^***^ | - | 0.204 | 0.194 | 0.085 | 0.168 | 0.329 | 0.274 | 0.226 | 0.220 | 0.312 | 0.222 | 0.207 | 0.254 | 0.257 | 0.178 | 0.226 | 0.179 |
| SZDSZ | 0.133^***^ | 0.373^***^ | 0.269^***^ | 0.394^***^ | 0.284^***^ | - | 0.132 | 0.249 | 0.275 | 0.311 | 0.245 | 0.229 | 0.303 | 0.250 | 0.372 | 0.374 | 0.339 | 0.267 | 0.242 | 0.196 | 0.161 |
| SZLTS | 0.044^**^ | 0.374^***^ | 0.358^***^ | 0.358^***^ | 0.317^***^ | 0.169^***^ | - | 0.222 | 0.285 | 0.303 | 0.271 | 0.338 | 0.329 | 0.269 | 0.383 | 0.364 | 0.341 | 0.313 | 0.341 | 0.319 | 0.225 |
| YNGLG | 0.361^***^ | 0.278^***^ | 0.307^***^ | 0.548^***^ | 0.162^***^ | 0.364^***^ | 0.408^***^ | - | 0.175 | 0.394 | 0.275 | 0.211 | 0.217 | 0.298 | 0.229 | 0.149 | 0.237 | 0.261 | 0.147 | 0.239 | 0.182 |
| YNTC | 0.335^***^ | 0.355^***^ | 0.159^***^ | 0.501^***^ | 0.239^***^ | 0.315^***^ | 0.369^***^ | 0.307^***^ | - | 0.386 | 0.261 | 0.148 | 0.184 | 0.270 | 0.290 | 0.283 | 0.274 | 0.331 | 0.252 | 0.237 | 0.257 |
| MSGH | 0.380^***^ | 0.566^***^ | 0.526^***^ | 0.528^***^ | 0.474^***^ | 0.406^***^ | 0.423^***^ | 0.565^***^ | 0.494^***^ | - | 0.296 | 0.375 | 0.365 | 0.305 | 0.393 | 0.468 | 0.432 | 0.381 | 0.460 | 0.424 | 0.372 |
| YTLHS | 0.303^***^ | 0.411^***^ | 0.340^***^ | 0.383^***^ | 0.313^***^ | 0.266^***^ | 0.354^***^ | 0.356^***^ | 0.270^***^ | 0.395^***^ | - | 0.189 | 0.150 | 0.205 | 0.320 | 0.306 | 0.288 | 0.322 | 0.313 | 0.285 | 0.265 |
| LBMH | 0.334^***^ | 0.362^***^ | 0.165^***^ | 0.430^***^ | 0.265^***^ | 0.249^***^ | 0.380^***^ | 0.295^***^ | 0.168^***^ | 0.436^***^ | 0.152^***^ | - | 0.151 | 0.265 | 0.286 | 0.234 | 0.211 | 0.264 | 0.146 | 0.111 | 0.257 |
| CLHL | 0.358^***^ | 0.411^***^ | 0.332^***^ | 0.470^***^ | 0.308^***^ | 0.338^***^ | 0.416^***^ | 0.364^***^ | 0.245^***^ | 0.466^***^ | 0.118^***^ | 0.155^***^ | - | 0.181 | 0.227 | 0.185 | 0.199 | 0.277 | 0.209 | 0.245 | 0.205 |
| GDSYD | 0.314^***^ | 0.521^***^ | 0.433^***^ | 0.437^***^ | 0.436^***^ | 0.302^***^ | 0.378^***^ | 0.473^***^ | 0.386^***^ | 0.459^***^ | 0.268^***^ | 0.313^***^ | 0.289^***^ | - | 0.270 | 0.256 | 0.229 | 0.235 | 0.299 | 0.321 | 0.204 |
| QYBSZ | 0.562^***^ | 0.629^***^ | 0.530^***^ | 0.572^***^ | 0.459^***^ | 0.561^***^ | 0.609^***^ | 0.493^***^ | 0.503^***^ | 0.625^***^ | 0.478^***^ | 0.449^***^ | 0.463^***^ | 0.559^***^ | - | 0.093 | 0.104 | 0.100 | 0.275 | 0.335 | 0.285 |
| QYSYH | 0.521^***^ | 0.516^***^ | 0.504^***^ | 0.531^***^ | 0.428^***^ | 0.540^***^ | 0.579^***^ | 0.329^***^ | 0.478^***^ | 0.654^***^ | 0.446^***^ | 0.401^***^ | 0.429^***^ | 0.526^***^ | 0.353^***^ | - | 0.096 | 0.153 | 0.183 | 0.326 | 0.220 |
| SCHSY | 0.475^***^ | 0.558^***^ | 0.414^***^ | 0.442^***^ | 0.449^***^ | 0.492^***^ | 0.534^***^ | 0.449^***^ | 0.434^***^ | 0.615^***^ | 0.398^***^ | 0.339^***^ | 0.391^***^ | 0.461^***^ | 0.366^***^ | 0.321^***^ | - | 0.074 | 0.256 | 0.236 | 0.274 |
| NDXFS | 0.423^***^ | 0.564^***^ | 0.382^***^ | 0.406^***^ | 0.423^***^ | 0.410^***^ | 0.487^***^ | 0.434^***^ | 0.457^***^ | 0.564^***^ | 0.405^***^ | 0.352^***^ | 0.422^***^ | 0.418^***^ | 0.302^***^ | 0.361^***^ | 0.194^***^ | - | 0.223 | 0.215 | 0.262 |
| ZYFS | 0.432^***^ | 0.384^***^ | 0.238^***^ | 0.623^***^ | 0.298^***^ | 0.380^***^ | 0.493^***^ | 0.241^***^ | 0.401^***^ | 0.617^***^ | 0.408^***^ | 0.275^***^ | 0.396^***^ | 0.505^***^ | 0.598^***^ | 0.459^***^ | 0.516^***^ | 0.450^***^ | - | 0.120 | 0.196 |
| LCFBS | 0.439^***^ | 0.516^***^ | 0.151^***^ | 0.605^***^ | 0.372^***^ | 0.339^***^ | 0.491^***^ | 0.402^***^ | 0.388^***^ | 0.597^***^ | 0.359^***^ | 0.184^***^ | 0.382^***^ | 0.508^***^ | 0.620^***^ | 0.593^***^ | 0.452^***^ | 0.430^***^ | 0.323^***^ | - | 0.273 |
| TWYL | 0.260^***^ | 0.357^***^ | 0.341^***^ | 0.486^***^ | 0.251^***^ | 0.218^***^ | 0.325^***^ | 0.255^***^ | 0.314^***^ | 0.490^***^ | 0.288^***^ | 0.287^***^ | 0.284^***^ | 0.334^***^ | 0.492^***^ | 0.421^***^ | 0.452^***^ | 0.411^***^ | 0.327^***^ | 0.397^***^ | - |

*/ **/ *** Significant difference (*P* < 0.1/ 0.05/ 0.001)
