# Supplementary material for: Genetic diversity and population structure of the endangered basal angiosperm Brasenia schreberi (Cabombaceae) in China
Source: PeerJ. 2018 Jul 13;6:e5296. doi: 10.7717/peerj.5296 (PMC6047506; doi:10.7717/peerj.5296)
Supplement: Supplemental Information 3 — The sequences of six tested individuals for each DNA region were listed below the figure. [file peerj-06-5296-s003.doc]

**Figure S1** Figures showing the alignment of six sequences from different populations for each of the tested twenty chloroplast non-condoing sequences and the nuclear ITS region (the sequences of six tested individuals for each DNA region were listed below the figure).

*rpL16 intron* (pairwise identity: 100%)


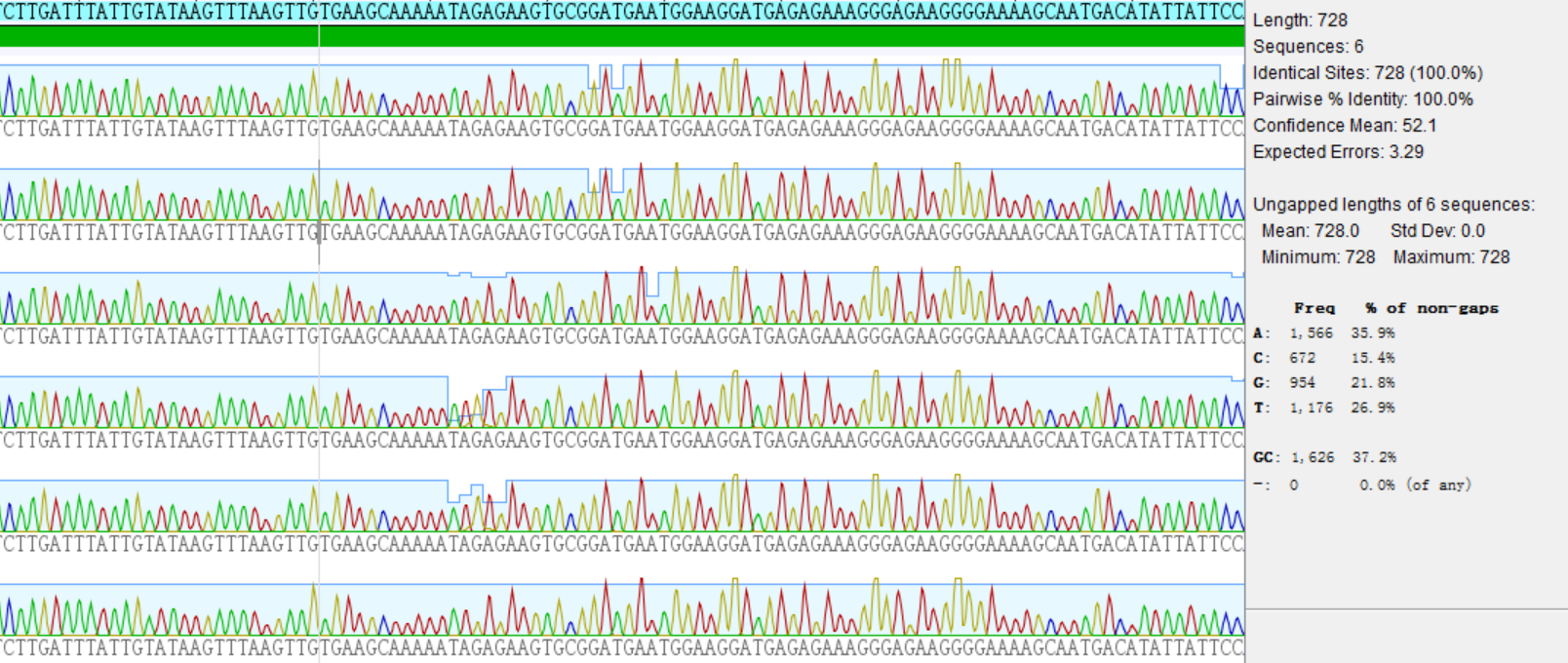


>NXQZS_1

GCTCATTGCTTCGTATTCTCGAGATCCAAGGAATCGGTCATTATATGAGATAATAATCATATAGTTGTAGCAACTGAAATCCTTTTTCAATAAAGTAAAAATGAATCTTGATTTATTGTATAAGTTTAAGTTGTGAAGCAAAAATAGAGAAGTGCGGATGAATGGAAGGATGAGAGAAAGGGAGAAGGGGAAAAGCAATGACATATTATTCCAATATGTATGGTCTATCAATCATCTCAGAAAGGACAATGTGATAAGGCATCAATACTCTAATTCAATTCATCTAAAATTTTTAGATAGATTTCATAGGAGAAAAAAACGAGCCTTGAGTCGATAGAGACTGAGGAGATTGACTCATGTACGGATTACTTTAGAAGCTCCATTGTAGAATTAAGACCTCGACATTAATGGAGGAGAAGCTATGGGAACGACGGAACCTGTGACTGCGGAGGATTCGATTGAAAACGAATCCTAATGATTCATCGGACGGGATGGCGGAAGACGCCGAGATCGAACTTATTTCTTCGAAGAGGTTATGGAACGACCCTACGAATAAAGCAAAAAAAAATAAAAGAGTAAATATTCGCCCGCGAAATTTCATTCAGTAAATAATTTTACTATTATTTATTCTTTCATTCGCGAGGAGCTGGATGAGAAGAAACTCTCATGTCCGGTTCTGTAGTAGAGATGGGATTGAGGCACTACCATCAACTATAACCCCAAAAGAACCAGGTT

>GDSYD_1

GCTCATTGCTTCGTATTCTCGAGATCCAAGGAATCGGTCATTATATGAGATAATAATCATATAGTTGTAGCAACTGAAATCCTTTTTCAATAAAGTAAAAATGAATCTTGATTTATTGTATAAGTTTAAGTTGTGAAGCAAAAATAGAGAAGTGCGGATGAATGGAAGGATGAGAGAAAGGGAGAAGGGGAAAAGCAATGACATATTATTCCAATATGTATGGTCTATCAATCATCTCAGAAAGGACAATGTGATAAGGCATCAATACTCTAATTCAATTCATCTAAAATTTTTAGATAGATTTCATAGGAGAAAAAAACGAGCCTTGAGTCGATAGAGACTGAGGAGATTGACTCATGTACGGATTACTTTAGAAGCTCCATTGTAGAATTAAGACCTCGACATTAATGGAGGAGAAGCTATGGGAACGACGGAACCTGTGACTGCGGAGGATTCGATTGAAAACGAATCCTAATGATTCATCGGACGGGATGGCGGAAGACGCCGAGATCGAACTTATTTCTTCGAAGAGGTTATGGAACGACCCTACGAATAAAGCAAAAAAAAATAAAAGAGTAAATATTCGCCCGCGAAATTTCATTCAGTAAATAATTTTACTATTATTTATTCTTTCATTCGCGAGGAGCTGGATGAGAAGAAACTCTCATGTCCGGTTCTGTAGTAGAGATGGGATTGAGGCACTACCATCAACTATAACCCCAAAAGAACCAGGTT

>NDXFS_1

GCTCATTGCTTCGTATTCTCGAGATCCAAGGAATCGGTCATTATATGAGATAATAATCATATAGTTGTAGCAACTGAAATCCTTTTTCAATAAAGTAAAAATGAATCTTGATTTATTGTATAAGTTTAAGTTGTGAAGCAAAAATAGAGAAGTGCGGATGAATGGAAGGATGAGAGAAAGGGAGAAGGGGAAAAGCAATGACATATTATTCCAATATGTATGGTCTATCAATCATCTCAGAAAGGACAATGTGATAAGGCATCAATACTCTAATTCAATTCATCTAAAATTTTTAGATAGATTTCATAGGAGAAAAAAACGAGCCTTGAGTCGATAGAGACTGAGGAGATTGACTCATGTACGGATTACTTTAGAAGCTCCATTGTAGAATTAAGACCTCGACATTAATGGAGGAGAAGCTATGGGAACGACGGAACCTGTGACTGCGGAGGATTCGATTGAAAACGAATCCTAATGATTCATCGGACGGGATGGCGGAAGACGCCGAGATCGAACTTATTTCTTCGAAGAGGTTATGGAACGACCCTACGAATAAAGCAAAAAAAAATAAAAGAGTAAATATTCGCCCGCGAAATTTCATTCAGTAAATAATTTTACTATTATTTATTCTTTCATTCGCGAGGAGCTGGATGAGAAGAAACTCTCATGTCCGGTTCTGTAGTAGAGATGGGATTGAGGCACTACCATCAACTATAACCCCAAAAGAACCAGGTT

>TWYL_1

GCTCATTGCTTCGTATTCTCGAGATCCAAGGAATCGGTCATTATATGAGATAATAATCATATAGTTGTAGCAACTGAAATCCTTTTTCAATAAAGTAAAAATGAATCTTGATTTATTGTATAAGTTTAAGTTGTGAAGCAAAAATAGAGAAGTGCGGATGAATGGAAGGATGAGAGAAAGGGAGAAGGGGAAAAGCAATGACATATTATTCCAATATGTATGGTCTATCAATCATCTCAGAAAGGACAATGTGATAAGGCATCAATACTCTAATTCAATTCATCTAAAATTTTTAGATAGATTTCATAGGAGAAAAAAACGAGCCTTGAGTCGATAGAGACTGAGGAGATTGACTCATGTACGGATTACTTTAGAAGCTCCATTGTAGAATTAAGACCTCGACATTAATGGAGGAGAAGCTATGGGAACGACGGAACCTGTGACTGCGGAGGATTCGATTGAAAACGAATCCTAATGATTCATCGGACGGGATGGCGGAAGACGCCGAGATCGAACTTATTTCTTCGAAGAGGTTATGGAACGACCCTACGAATAAAGCAAAAAAAAATAAAAGAGTAAATATTCGCCCGCGAAATTTCATTCAGTAAATAATTTTACTATTATTTATTCTTTCATTCGCGAGGAGCTGGATGAGAAGAAACTCTCATGTCCGGTTCTGTAGTAGAGATGGGATTGAGGCACTACCATCAACTATAACCCCAAAAGAACCAGGTT

>QYBSZ_1

GCTCATTGCTTCGTATTCTCGAGATCCAAGGAATCGGTCATTATATGAGATAATAATCATATAGTTGTAGCAACTGAAATCCTTTTTCAATAAAGTAAAAATGAATCTTGATTTATTGTATAAGTTTAAGTTGTGAAGCAAAAATAGAGAAGTGCGGATGAATGGAAGGATGAGAGAAAGGGAGAAGGGGAAAAGCAATGACATATTATTCCAATATGTATGGTCTATCAATCATCTCAGAAAGGACAATGTGATAAGGCATCAATACTCTAATTCAATTCATCTAAAATTTTTAGATAGATTTCATAGGAGAAAAAAACGAGCCTTGAGTCGATAGAGACTGAGGAGATTGACTCATGTACGGATTACTTTAGAAGCTCCATTGTAGAATTAAGACCTCGACATTAATGGAGGAGAAGCTATGGGAACGACGGAACCTGTGACTGCGGAGGATTCGATTGAAAACGAATCCTAATGATTCATCGGACGGGATGGCGGAAGACGCCGAGATCGAACTTATTTCTTCGAAGAGGTTATGGAACGACCCTACGAATAAAGCAAAAAAAAATAAAAGAGTAAATATTCGCCCGCGAAATTTCATTCAGTAAATAATTTTACTATTATTTATTCTTTCATTCGCGAGGAGCTGGATGAGAAGAAACTCTCATGTCCGGTTCTGTAGTAGAGATGGGATTGAGGCACTACCATCAACTATAACCCCAAAAGAACCAGGTT

>YNTC_1

GCTCATTGCTTCGTATTCTCGAGATCCAAGGAATCGGTCATTATATGAGATAATAATCATATAGTTGTAGCAACTGAAATCCTTTTTCAATAAAGTAAAAATGAATCTTGATTTATTGTATAAGTTTAAGTTGTGAAGCAAAAATAGAGAAGTGCGGATGAATGGAAGGATGAGAGAAAGGGAGAAGGGGAAAAGCAATGACATATTATTCCAATATGTATGGTCTATCAATCATCTCAGAAAGGACAATGTGATAAGGCATCAATACTCTAATTCAATTCATCTAAAATTTTTAGATAGATTTCATAGGAGAAAAAAACGAGCCTTGAGTCGATAGAGACTGAGGAGATTGACTCATGTACGGATTACTTTAGAAGCTCCATTGTAGAATTAAGACCTCGACATTAATGGAGGAGAAGCTATGGGAACGACGGAACCTGTGACTGCGGAGGATTCGATTGAAAACGAATCCTAATGATTCATCGGACGGGATGGCGGAAGACGCCGAGATCGAACTTATTTCTTCGAAGAGGTTATGGAACGACCCTACGAATAAAGCAAAAAAAAATAAAAGAGTAAATATTCGCCCGCGAAATTTCATTCAGTAAATAATTTTACTATTATTTATTCTTTCATTCGCGAGGAGCTGGATGAGAAGAAACTCTCATGTCCGGTTCTGTAGTAGAGATGGGATTGAGGCACTACCATCAACTATAACCCCAAAAGAACCAGGTT

*trnC-ycf6* (pairwise identity: 100%)


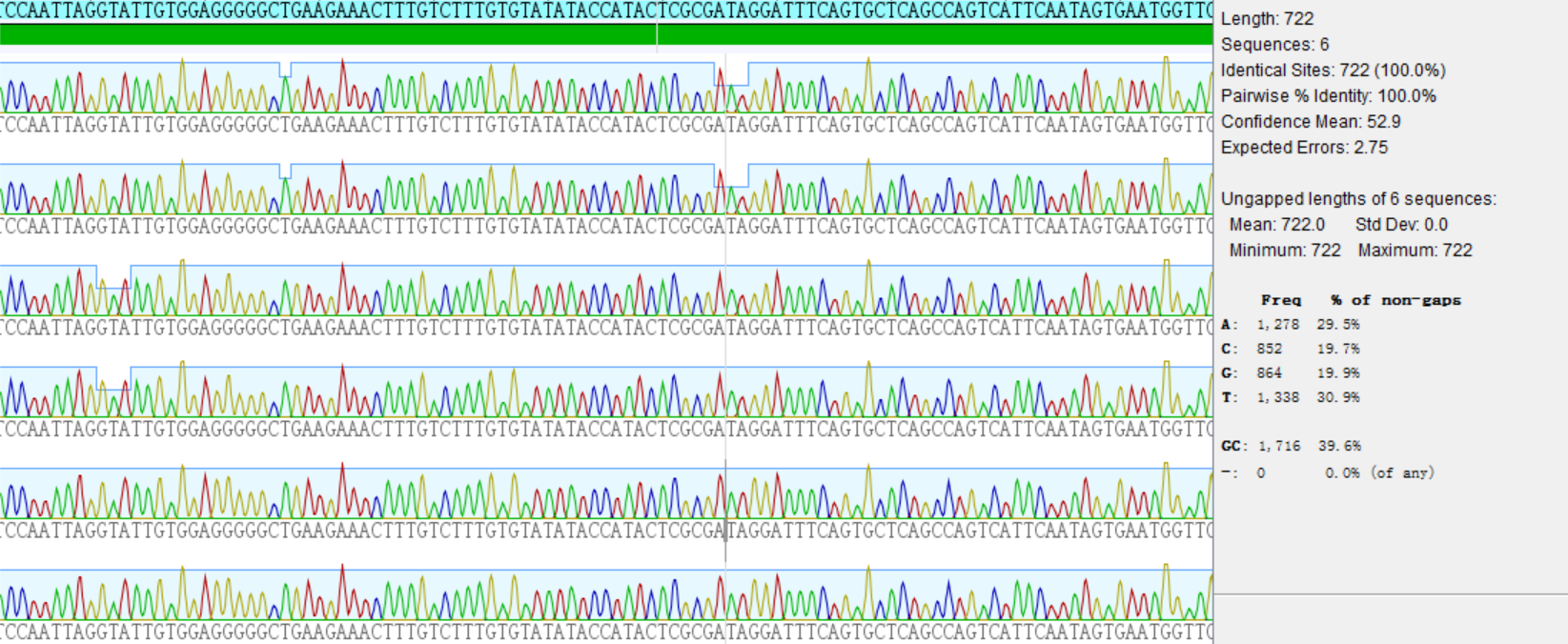


>NXQZS_1

ATCCAGTTTCGCTGATATAACTCGCCCGATTTTTGACCGGCGGACGAAAAGAGCTGTCCATACTTTATTTATCTTCAAAATAAGCTTCAAAATACGCAGGTTCTATGAAAGAAATAGAACTCTTTGCTCCGAGGATCCCATCCAGGGGGCGTGACTTATTGTAAAGACTATAACTATCAATTTTTACTAATTACTTTACCCTACCAGTAATCCACTAATATCCACTGGAGTGTCTCCTGATTCCGTCGTGAATTAGATCTTGCTTGGATTCGCCTTGGGGGAATCCAATTAGGTATTGTGGAGGGGGCTGAAGAAACTTTGTCTTTGTGTATATACCATACTCGCGATAGGATTTCAGTGCTCAGCCAGTCATTCAATAGTGAATGGTTGACTGGTCCCTATAACTATAAAGTAAAAAAGAACTATGACACCCGTAATAGGGTGTCTAACGATTGTTTGACAGAAACAGAGATAGTAAGGTTTAGCTTAGATCAAGGGGAGATACAGTTATCCATCTTGATGGTATATATGTATATATAGATTTTCCCTATGAAAAGCAACAAAACAAAGAATAGGTCTTACTATTAGGACATGTTCTATTCCCTTAGTAGCATCCCCTTGCTACTTCCAATGGGTAACGTGTATCTGTTCCGCTTGGGCTTAATTCTTCCTCACTAGAAATCCGGAAATATAAACTTGTTACAAGCGGATCCATTGGATCGGTTTATCAATAGCTTTAAGTCATAATGTCATTTTTTTGACTCTGCACCACCAATTCCCCTATTATTATTTTAGGGATCAATAATGTAACAATTCCTTCATATTCATCG

>GDSYD_1

ATCCAGTTTCGCTGATATAACTCGCCCGATTTTTGACCGGCGGACGAAAAGAGCTGTCCATACTTTATTTATCTTCAAAATAAGCTTCAAAATACGCAGGTTCTATGAAAGAAATAGAACTCTTTGCTCCGAGGATCCCATCCAGGGGGCGTGACTTATTGTAAAGACTATAACTATCAATTTTTACTAATTACTTTACCCTACCAGTAATCCACTAATATCCACTGGAGTGTCTCCTGATTCCGTCGTGAATTAGATCTTGCTTGGATTCGCCTTGGGGGAATCCAATTAGGTATTGTGGAGGGGGCTGAAGAAACTTTGTCTTTGTGTATATACCATACTCGCGATAGGATTTCAGTGCTCAGCCAGTCATTCAATAGTGAATGGTTGACTGGTCCCTATAACTATAAAGTAAAAAAGAACTATGACACCCGTAATAGGGTGTCTAACGATTGTTTGACAGAAACAGAGATAGTAAGGTTTAGCTTAGATCAAGGGGAGATACAGTTATCCATCTTGATGGTATATATGTATATATAGATTTTCCCTATGAAAAGCAACAAAACAAAGAATAGGTCTTACTATTAGGACATGTTCTATTCCCTTAGTAGCATCCCCTTGCTACTTCCAATGGGTAACGTGTATCTGTTCCGCTTGGGCTTAATTCTTCCTCACTAGAAATCCGGAAATATAAACTTGTTACAAGCGGATCCATTGGATCGGTTTATCAATAGCTTTAAGTCATAATGTCATTTTTTTGACTCTGCACCACCAATTCCCCTATTATTATTTTAGGGATCAATAATGTAACAATTCCTTCATATTCATCG

>NDXFS_1

ATCCAGTTTCGCTGATATAACTCGCCCGATTTTTGACCGGCGGACGAAAAGAGCTGTCCATACTTTATTTATCTTCAAAATAAGCTTCAAAATACGCAGGTTCTATGAAAGAAATAGAACTCTTTGCTCCGAGGATCCCATCCAGGGGGCGTGACTTATTGTAAAGACTATAACTATCAATTTTTACTAATTACTTTACCCTACCAGTAATCCACTAATATCCACTGGAGTGTCTCCTGATTCCGTCGTGAATTAGATCTTGCTTGGATTCGCCTTGGGGGAATCCAATTAGGTATTGTGGAGGGGGCTGAAGAAACTTTGTCTTTGTGTATATACCATACTCGCGATAGGATTTCAGTGCTCAGCCAGTCATTCAATAGTGAATGGTTGACTGGTCCCTATAACTATAAAGTAAAAAAGAACTATGACACCCGTAATAGGGTGTCTAACGATTGTTTGACAGAAACAGAGATAGTAAGGTTTAGCTTAGATCAAGGGGAGATACAGTTATCCATCTTGATGGTATATATGTATATATAGATTTTCCCTATGAAAAGCAACAAAACAAAGAATAGGTCTTACTATTAGGACATGTTCTATTCCCTTAGTAGCATCCCCTTGCTACTTCCAATGGGTAACGTGTATCTGTTCCGCTTGGGCTTAATTCTTCCTCACTAGAAATCCGGAAATATAAACTTGTTACAAGCGGATCCATTGGATCGGTTTATCAATAGCTTTAAGTCATAATGTCATTTTTTTGACTCTGCACCACCAATTCCCCTATTATTATTTTAGGGATCAATAATGTAACAATTCCTTCATATTCATCG

>TWYL_1

ATCCAGTTTCGCTGATATAACTCGCCCGATTTTTGACCGGCGGACGAAAAGAGCTGTCCATACTTTATTTATCTTCAAAATAAGCTTCAAAATACGCAGGTTCTATGAAAGAAATAGAACTCTTTGCTCCGAGGATCCCATCCAGGGGGCGTGACTTATTGTAAAGACTATAACTATCAATTTTTACTAATTACTTTACCCTACCAGTAATCCACTAATATCCACTGGAGTGTCTCCTGATTCCGTCGTGAATTAGATCTTGCTTGGATTCGCCTTGGGGGAATCCAATTAGGTATTGTGGAGGGGGCTGAAGAAACTTTGTCTTTGTGTATATACCATACTCGCGATAGGATTTCAGTGCTCAGCCAGTCATTCAATAGTGAATGGTTGACTGGTCCCTATAACTATAAAGTAAAAAAGAACTATGACACCCGTAATAGGGTGTCTAACGATTGTTTGACAGAAACAGAGATAGTAAGGTTTAGCTTAGATCAAGGGGAGATACAGTTATCCATCTTGATGGTATATATGTATATATAGATTTTCCCTATGAAAAGCAACAAAACAAAGAATAGGTCTTACTATTAGGACATGTTCTATTCCCTTAGTAGCATCCCCTTGCTACTTCCAATGGGTAACGTGTATCTGTTCCGCTTGGGCTTAATTCTTCCTCACTAGAAATCCGGAAATATAAACTTGTTACAAGCGGATCCATTGGATCGGTTTATCAATAGCTTTAAGTCATAATGTCATTTTTTTGACTCTGCACCACCAATTCCCCTATTATTATTTTAGGGATCAATAATGTAACAATTCCTTCATATTCATCG

>QYBSZ_1

ATCCAGTTTCGCTGATATAACTCGCCCGATTTTTGACCGGCGGACGAAAAGAGCTGTCCATACTTTATTTATCTTCAAAATAAGCTTCAAAATACGCAGGTTCTATGAAAGAAATAGAACTCTTTGCTCCGAGGATCCCATCCAGGGGGCGTGACTTATTGTAAAGACTATAACTATCAATTTTTACTAATTACTTTACCCTACCAGTAATCCACTAATATCCACTGGAGTGTCTCCTGATTCCGTCGTGAATTAGATCTTGCTTGGATTCGCCTTGGGGGAATCCAATTAGGTATTGTGGAGGGGGCTGAAGAAACTTTGTCTTTGTGTATATACCATACTCGCGATAGGATTTCAGTGCTCAGCCAGTCATTCAATAGTGAATGGTTGACTGGTCCCTATAACTATAAAGTAAAAAAGAACTATGACACCCGTAATAGGGTGTCTAACGATTGTTTGACAGAAACAGAGATAGTAAGGTTTAGCTTAGATCAAGGGGAGATACAGTTATCCATCTTGATGGTATATATGTATATATAGATTTTCCCTATGAAAAGCAACAAAACAAAGAATAGGTCTTACTATTAGGACATGTTCTATTCCCTTAGTAGCATCCCCTTGCTACTTCCAATGGGTAACGTGTATCTGTTCCGCTTGGGCTTAATTCTTCCTCACTAGAAATCCGGAAATATAAACTTGTTACAAGCGGATCCATTGGATCGGTTTATCAATAGCTTTAAGTCATAATGTCATTTTTTTGACTCTGCACCACCAATTCCCCTATTATTATTTTAGGGATCAATAATGTAACAATTCCTTCATATTCATCG

>YNTC_1

ATCCAGTTTCGCTGATATAACTCGCCCGATTTTTGACCGGCGGACGAAAAGAGCTGTCCATACTTTATTTATCTTCAAAATAAGCTTCAAAATACGCAGGTTCTATGAAAGAAATAGAACTCTTTGCTCCGAGGATCCCATCCAGGGGGCGTGACTTATTGTAAAGACTATAACTATCAATTTTTACTAATTACTTTACCCTACCAGTAATCCACTAATATCCACTGGAGTGTCTCCTGATTCCGTCGTGAATTAGATCTTGCTTGGATTCGCCTTGGGGGAATCCAATTAGGTATTGTGGAGGGGGCTGAAGAAACTTTGTCTTTGTGTATATACCATACTCGCGATAGGATTTCAGTGCTCAGCCAGTCATTCAATAGTGAATGGTTGACTGGTCCCTATAACTATAAAGTAAAAAAGAACTATGACACCCGTAATAGGGTGTCTAACGATTGTTTGACAGAAACAGAGATAGTAAGGTTTAGCTTAGATCAAGGGGAGATACAGTTATCCATCTTGATGGTATATATGTATATATAGATTTTCCCTATGAAAAGCAACAAAACAAAGAATAGGTCTTACTATTAGGACATGTTCTATTCCCTTAGTAGCATCCCCTTGCTACTTCCAATGGGTAACGTGTATCTGTTCCGCTTGGGCTTAATTCTTCCTCACTAGAAATCCGGAAATATAAACTTGTTACAAGCGGATCCATTGGATCGGTTTATCAATAGCTTTAAGTCATAATGTCATTTTTTTGACTCTGCACCACCAATTCCCCTATTATTATTTTAGGGATCAATAATGTAACAATTCCTTCATATTCATCG

*petL-psbE* (pairwise identity: 100%)


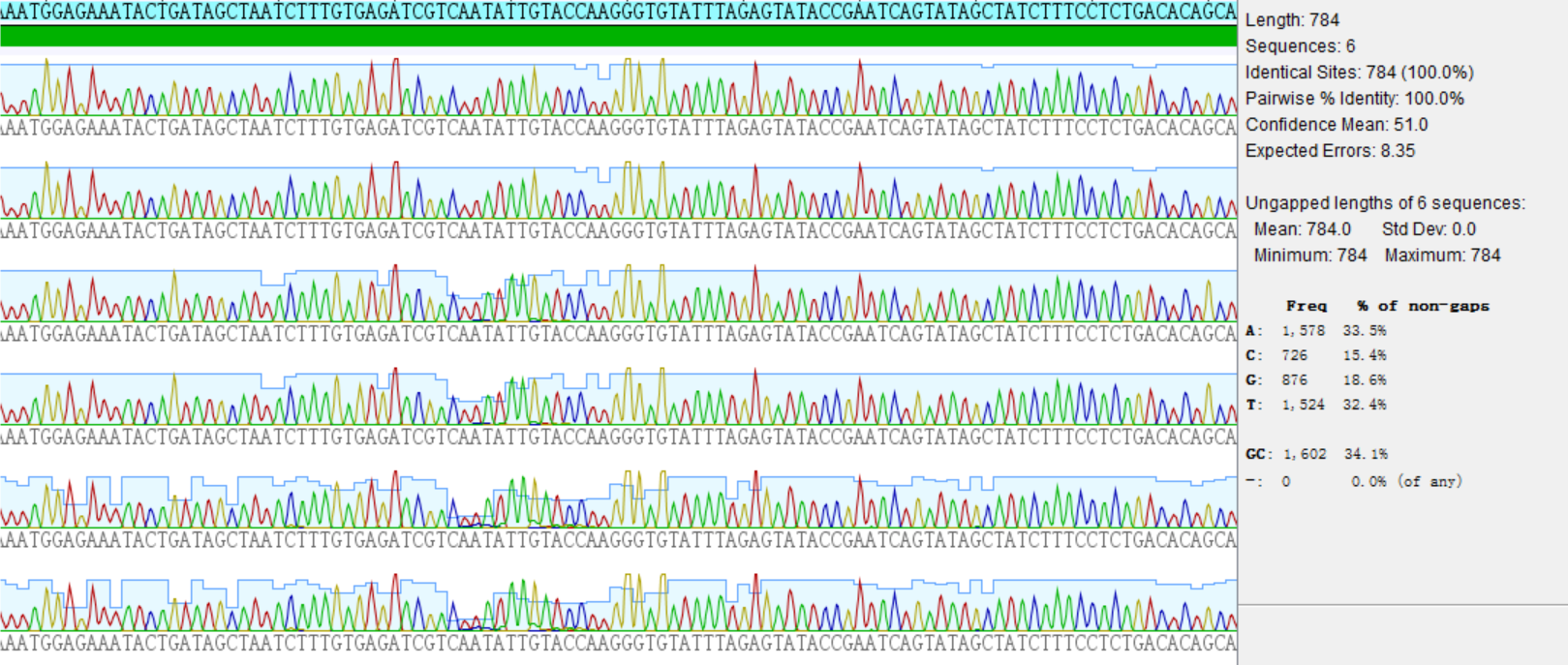


>NXQZS_1

TGAGCTCCAATATTACAGTCAAAGGGGGATCGATTTCGTGAAAGATAGAGATCAGTAAATGGAGAAATACTGATAGCTAATCTTTGTGAGATCGTCAATATTGTACCAAGGGTGTATTTAGAGTATACCGAATCAGTATAGCTATCTTTCCTCTGACACAGCAATGTTGTTTCAATCAGCATCGAAAAAAATGGTACAGAATTCCTTTCTTCCTTTCTTGTTCCTTGTATAGGCCTAAGCAGGTGTCATTCAATAGAATAGAAAATTCCTTGTACTATAATGTTTTACTTTTTAGCGGTTTCGGAATAGGCTGAAGATCTTGGTAAAGCTACTCGATGGCTTTTCTTTTTTATTTAATTAATCTATCTAATAATTCATGAATTAAATTATCATATTCCCAAAATTGGATGCGAAATCTGGAAATCTGGAAACTATTTTTCGTGGTTGTAGAATGGTTGTAGAAGTAGAAAAAGATATTTTCGTTCTAATCCCTTCCCTTCCAATAAGAGGGTTTGTTTGGACAATACATAGTATTCCATAAAAGAAAGTGAACAAACAATATTTGGAGTAGTCGAAATTCGTGATGCAACTATAATGGAATGAGTTGAACTAAACTGTTGTTTCATTAGATTGTTTCATTAGGTCCAATCCAAGGGTTCCCGCCTATAAGAAGATAGGACTACATCATGTGAAAAGAAAAAAAATGTGGAACCCAGAACAAATACAAATAATAGTAATTGCTAATCTTGGAATCCAGTTGTACCTTAAATAACGCTTTTTTTTTTTTTGCTT

>GDSYD_1

TGAGCTCCAATATTACAGTCAAAGGGGGATCGATTTCGTGAAAGATAGAGATCAGTAAATGGAGAAATACTGATAGCTAATCTTTGTGAGATCGTCAATATTGTACCAAGGGTGTATTTAGAGTATACCGAATCAGTATAGCTATCTTTCCTCTGACACAGCAATGTTGTTTCAATCAGCATCGAAAAAAATGGTACAGAATTCCTTTCTTCCTTTCTTGTTCCTTGTATAGGCCTAAGCAGGTGTCATTCAATAGAATAGAAAATTCCTTGTACTATAATGTTTTACTTTTTAGCGGTTTCGGAATAGGCTGAAGATCTTGGTAAAGCTACTCGATGGCTTTTCTTTTTTATTTAATTAATCTATCTAATAATTCATGAATTAAATTATCATATTCCCAAAATTGGATGCGAAATCTGGAAATCTGGAAACTATTTTTCGTGGTTGTAGAATGGTTGTAGAAGTAGAAAAAGATATTTTCGTTCTAATCCCTTCCCTTCCAATAAGAGGGTTTGTTTGGACAATACATAGTATTCCATAAAAGAAAGTGAACAAACAATATTTGGAGTAGTCGAAATTCGTGATGCAACTATAATGGAATGAGTTGAACTAAACTGTTGTTTCATTAGATTGTTTCATTAGGTCCAATCCAAGGGTTCCCGCCTATAAGAAGATAGGACTACATCATGTGAAAAGAAAAAAAATGTGGAACCCAGAACAAATACAAATAATAGTAATTGCTAATCTTGGAATCCAGTTGTACCTTAAATAACGCTTTTTTTTTTTTTGCTT

>NDXFS_1

TGAGCTCCAATATTACAGTCAAAGGGGGATCGATTTCGTGAAAGATAGAGATCAGTAAATGGAGAAATACTGATAGCTAATCTTTGTGAGATCGTCAATATTGTACCAAGGGTGTATTTAGAGTATACCGAATCAGTATAGCTATCTTTCCTCTGACACAGCAATGTTGTTTCAATCAGCATCGAAAAAAATGGTACAGAATTCCTTTCTTCCTTTCTTGTTCCTTGTATAGGCCTAAGCAGGTGTCATTCAATAGAATAGAAAATTCCTTGTACTATAATGTTTTACTTTTTAGCGGTTTCGGAATAGGCTGAAGATCTTGGTAAAGCTACTCGATGGCTTTTCTTTTTTATTTAATTAATCTATCTAATAATTCATGAATTAAATTATCATATTCCCAAAATTGGATGCGAAATCTGGAAATCTGGAAACTATTTTTCGTGGTTGTAGAATGGTTGTAGAAGTAGAAAAAGATATTTTCGTTCTAATCCCTTCCCTTCCAATAAGAGGGTTTGTTTGGACAATACATAGTATTCCATAAAAGAAAGTGAACAAACAATATTTGGAGTAGTCGAAATTCGTGATGCAACTATAATGGAATGAGTTGAACTAAACTGTTGTTTCATTAGATTGTTTCATTAGGTCCAATCCAAGGGTTCCCGCCTATAAGAAGATAGGACTACATCATGTGAAAAGAAAAAAAATGTGGAACCCAGAACAAATACAAATAATAGTAATTGCTAATCTTGGAATCCAGTTGTACCTTAAATAACGCTTTTTTTTTTTTTGCTT

>TWYL_1

TGAGCTCCAATATTACAGTCAAAGGGGGATCGATTTCGTGAAAGATAGAGATCAGTAAATGGAGAAATACTGATAGCTAATCTTTGTGAGATCGTCAATATTGTACCAAGGGTGTATTTAGAGTATACCGAATCAGTATAGCTATCTTTCCTCTGACACAGCAATGTTGTTTCAATCAGCATCGAAAAAAATGGTACAGAATTCCTTTCTTCCTTTCTTGTTCCTTGTATAGGCCTAAGCAGGTGTCATTCAATAGAATAGAAAATTCCTTGTACTATAATGTTTTACTTTTTAGCGGTTTCGGAATAGGCTGAAGATCTTGGTAAAGCTACTCGATGGCTTTTCTTTTTTATTTAATTAATCTATCTAATAATTCATGAATTAAATTATCATATTCCCAAAATTGGATGCGAAATCTGGAAATCTGGAAACTATTTTTCGTGGTTGTAGAATGGTTGTAGAAGTAGAAAAAGATATTTTCGTTCTAATCCCTTCCCTTCCAATAAGAGGGTTTGTTTGGACAATACATAGTATTCCATAAAAGAAAGTGAACAAACAATATTTGGAGTAGTCGAAATTCGTGATGCAACTATAATGGAATGAGTTGAACTAAACTGTTGTTTCATTAGATTGTTTCATTAGGTCCAATCCAAGGGTTCCCGCCTATAAGAAGATAGGACTACATCATGTGAAAAGAAAAAAAATGTGGAACCCAGAACAAATACAAATAATAGTAATTGCTAATCTTGGAATCCAGTTGTACCTTAAATAACGCTTTTTTTTTTTTTGCTT

>QYBSZ_1

TGAGCTCCAATATTACAGTCAAAGGGGGATCGATTTCGTGAAAGATAGAGATCAGTAAATGGAGAAATACTGATAGCTAATCTTTGTGAGATCGTCAATATTGTACCAAGGGTGTATTTAGAGTATACCGAATCAGTATAGCTATCTTTCCTCTGACACAGCAATGTTGTTTCAATCAGCATCGAAAAAAATGGTACAGAATTCCTTTCTTCCTTTCTTGTTCCTTGTATAGGCCTAAGCAGGTGTCATTCAATAGAATAGAAAATTCCTTGTACTATAATGTTTTACTTTTTAGCGGTTTCGGAATAGGCTGAAGATCTTGGTAAAGCTACTCGATGGCTTTTCTTTTTTATTTAATTAATCTATCTAATAATTCATGAATTAAATTATCATATTCCCAAAATTGGATGCGAAATCTGGAAATCTGGAAACTATTTTTCGTGGTTGTAGAATGGTTGTAGAAGTAGAAAAAGATATTTTCGTTCTAATCCCTTCCCTTCCAATAAGAGGGTTTGTTTGGACAATACATAGTATTCCATAAAAGAAAGTGAACAAACAATATTTGGAGTAGTCGAAATTCGTGATGCAACTATAATGGAATGAGTTGAACTAAACTGTTGTTTCATTAGATTGTTTCATTAGGTCCAATCCAAGGGTTCCCGCCTATAAGAAGATAGGACTACATCATGTGAAAAGAAAAAAAATGTGGAACCCAGAACAAATACAAATAATAGTAATTGCTAATCTTGGAATCCAGTTGTACCTTAAATAACGCTTTTTTTTTTTTTGCTT

>YNTC_1

TGAGCTCCAATATTACAGTCAAAGGGGGATCGATTTCGTGAAAGATAGAGATCAGTAAATGGAGAAATACTGATAGCTAATCTTTGTGAGATCGTCAATATTGTACCAAGGGTGTATTTAGAGTATACCGAATCAGTATAGCTATCTTTCCTCTGACACAGCAATGTTGTTTCAATCAGCATCGAAAAAAATGGTACAGAATTCCTTTCTTCCTTTCTTGTTCCTTGTATAGGCCTAAGCAGGTGTCATTCAATAGAATAGAAAATTCCTTGTACTATAATGTTTTACTTTTTAGCGGTTTCGGAATAGGCTGAAGATCTTGGTAAAGCTACTCGATGGCTTTTCTTTTTTATTTAATTAATCTATCTAATAATTCATGAATTAAATTATCATATTCCCAAAATTGGATGCGAAATCTGGAAATCTGGAAACTATTTTTCGTGGTTGTAGAATGGTTGTAGAAGTAGAAAAAGATATTTTCGTTCTAATCCCTTCCCTTCCAATAAGAGGGTTTGTTTGGACAATACATAGTATTCCATAAAAGAAAGTGAACAAACAATATTTGGAGTAGTCGAAATTCGTGATGCAACTATAATGGAATGAGTTGAACTAAACTGTTGTTTCATTAGATTGTTTCATTAGGTCCAATCCAAGGGTTCCCGCCTATAAGAAGATAGGACTACATCATGTGAAAAGAAAAAAAATGTGGAACCCAGAACAAATACAAATAATAGTAATTGCTAATCTTGGAATCCAGTTGTACCTTAAATAACGCTTTTTTTTTTTTTGCTT

*atpI-atpH* (pairwise identity: 100%)


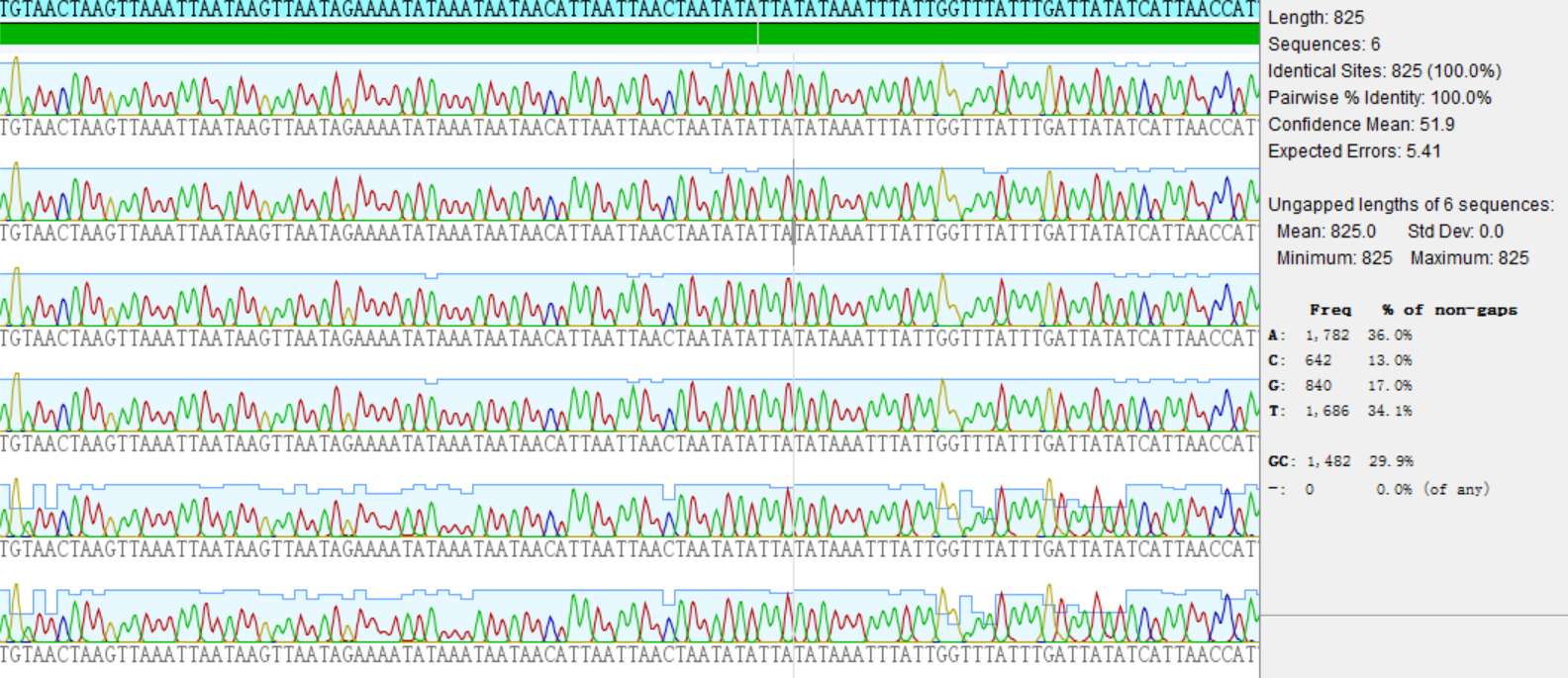


>NXQZS_1

CGGCGGCTTATATAGGTGAATCCATGGAAGGTCATCATTGAGTAGCTTTACATCTAAATAAGAAAATAATGCTTTGAATCGCGTTTTATTTTTGAAATTTATTGCAATTAAAAATGAAGCCCATGATTCTTATTCTAATAAGAAATTCATGGGTAACTCAAGATACCGAACATCTAAGGTCCGATCCGATTCAAATAAGATGGCGATGGTTTCCATTATGGAAGAAACACATGTATATGTGATAGTAGATATTCAATAGTTATATATGGACTACCCATCTATATTATTTCATTACCCATATTACCGACTTTGTATAGATATAGATTCCACTTTATATGGGTTACGATATAAGCTCTTCTTTTTTTCGTCTGTGACTGTAATGTCTGGATTGAATATGAAGTCAAGCCTATGAATGCATATAGAGAAGATGAAGGAACGAAGAGTTGAAAGAATGAGTTCGGAACAAAAACAAAACAAAGAAATGGAAGAACTAGAACTCTACGGATTTACAAAGACTTGTATAGGGAATAAAAACGAGATATTGAAGTAGTTCGTATGATTCAGTAATATCAAATATCATCACTTATCAAATTAGGTTCAGAGTTCTTAGTTTAGTTGTATGGATCTGAGTTATGCAATATGAAATAATAAGTAATGTAACTAAGTTAAATTAATAAGTTAATAGAAAATATAAATAATAACATTAATTAACTAATATATTATATAAATTTATTGGTTTATTTGATTATATCATTAACCATTTCTTCATTTTTTGTTGTGAGGAGCTTACCATGAATCCCATAATTTCTGCTGCTTCCG

>GDSYD_1

CGGCGGCTTATATAGGTGAATCCATGGAAGGTCATCATTGAGTAGCTTTACATCTAAATAAGAAAATAATGCTTTGAATCGCGTTTTATTTTTGAAATTTATTGCAATTAAAAATGAAGCCCATGATTCTTATTCTAATAAGAAATTCATGGGTAACTCAAGATACCGAACATCTAAGGTCCGATCCGATTCAAATAAGATGGCGATGGTTTCCATTATGGAAGAAACACATGTATATGTGATAGTAGATATTCAATAGTTATATATGGACTACCCATCTATATTATTTCATTACCCATATTACCGACTTTGTATAGATATAGATTCCACTTTATATGGGTTACGATATAAGCTCTTCTTTTTTTCGTCTGTGACTGTAATGTCTGGATTGAATATGAAGTCAAGCCTATGAATGCATATAGAGAAGATGAAGGAACGAAGAGTTGAAAGAATGAGTTCGGAACAAAAACAAAACAAAGAAATGGAAGAACTAGAACTCTACGGATTTACAAAGACTTGTATAGGGAATAAAAACGAGATATTGAAGTAGTTCGTATGATTCAGTAATATCAAATATCATCACTTATCAAATTAGGTTCAGAGTTCTTAGTTTAGTTGTATGGATCTGAGTTATGCAATATGAAATAATAAGTAATGTAACTAAGTTAAATTAATAAGTTAATAGAAAATATAAATAATAACATTAATTAACTAATATATTATATAAATTTATTGGTTTATTTGATTATATCATTAACCATTTCTTCATTTTTTGTTGTGAGGAGCTTACCATGAATCCCATAATTTCTGCTGCTTCCG

>NDXFS_1

CGGCGGCTTATATAGGTGAATCCATGGAAGGTCATCATTGAGTAGCTTTACATCTAAATAAGAAAATAATGCTTTGAATCGCGTTTTATTTTTGAAATTTATTGCAATTAAAAATGAAGCCCATGATTCTTATTCTAATAAGAAATTCATGGGTAACTCAAGATACCGAACATCTAAGGTCCGATCCGATTCAAATAAGATGGCGATGGTTTCCATTATGGAAGAAACACATGTATATGTGATAGTAGATATTCAATAGTTATATATGGACTACCCATCTATATTATTTCATTACCCATATTACCGACTTTGTATAGATATAGATTCCACTTTATATGGGTTACGATATAAGCTCTTCTTTTTTTCGTCTGTGACTGTAATGTCTGGATTGAATATGAAGTCAAGCCTATGAATGCATATAGAGAAGATGAAGGAACGAAGAGTTGAAAGAATGAGTTCGGAACAAAAACAAAACAAAGAAATGGAAGAACTAGAACTCTACGGATTTACAAAGACTTGTATAGGGAATAAAAACGAGATATTGAAGTAGTTCGTATGATTCAGTAATATCAAATATCATCACTTATCAAATTAGGTTCAGAGTTCTTAGTTTAGTTGTATGGATCTGAGTTATGCAATATGAAATAATAAGTAATGTAACTAAGTTAAATTAATAAGTTAATAGAAAATATAAATAATAACATTAATTAACTAATATATTATATAAATTTATTGGTTTATTTGATTATATCATTAACCATTTCTTCATTTTTTGTTGTGAGGAGCTTACCATGAATCCCATAATTTCTGCTGCTTCCG

>TWYL_1

CGGCGGCTTATATAGGTGAATCCATGGAAGGTCATCATTGAGTAGCTTTACATCTAAATAAGAAAATAATGCTTTGAATCGCGTTTTATTTTTGAAATTTATTGCAATTAAAAATGAAGCCCATGATTCTTATTCTAATAAGAAATTCATGGGTAACTCAAGATACCGAACATCTAAGGTCCGATCCGATTCAAATAAGATGGCGATGGTTTCCATTATGGAAGAAACACATGTATATGTGATAGTAGATATTCAATAGTTATATATGGACTACCCATCTATATTATTTCATTACCCATATTACCGACTTTGTATAGATATAGATTCCACTTTATATGGGTTACGATATAAGCTCTTCTTTTTTTCGTCTGTGACTGTAATGTCTGGATTGAATATGAAGTCAAGCCTATGAATGCATATAGAGAAGATGAAGGAACGAAGAGTTGAAAGAATGAGTTCGGAACAAAAACAAAACAAAGAAATGGAAGAACTAGAACTCTACGGATTTACAAAGACTTGTATAGGGAATAAAAACGAGATATTGAAGTAGTTCGTATGATTCAGTAATATCAAATATCATCACTTATCAAATTAGGTTCAGAGTTCTTAGTTTAGTTGTATGGATCTGAGTTATGCAATATGAAATAATAAGTAATGTAACTAAGTTAAATTAATAAGTTAATAGAAAATATAAATAATAACATTAATTAACTAATATATTATATAAATTTATTGGTTTATTTGATTATATCATTAACCATTTCTTCATTTTTTGTTGTGAGGAGCTTACCATGAATCCCATAATTTCTGCTGCTTCCG

>QYBSZ_1

CGGCGGCTTATATAGGTGAATCCATGGAAGGTCATCATTGAGTAGCTTTACATCTAAATAAGAAAATAATGCTTTGAATCGCGTTTTATTTTTGAAATTTATTGCAATTAAAAATGAAGCCCATGATTCTTATTCTAATAAGAAATTCATGGGTAACTCAAGATACCGAACATCTAAGGTCCGATCCGATTCAAATAAGATGGCGATGGTTTCCATTATGGAAGAAACACATGTATATGTGATAGTAGATATTCAATAGTTATATATGGACTACCCATCTATATTATTTCATTACCCATATTACCGACTTTGTATAGATATAGATTCCACTTTATATGGGTTACGATATAAGCTCTTCTTTTTTTCGTCTGTGACTGTAATGTCTGGATTGAATATGAAGTCAAGCCTATGAATGCATATAGAGAAGATGAAGGAACGAAGAGTTGAAAGAATGAGTTCGGAACAAAAACAAAACAAAGAAATGGAAGAACTAGAACTCTACGGATTTACAAAGACTTGTATAGGGAATAAAAACGAGATATTGAAGTAGTTCGTATGATTCAGTAATATCAAATATCATCACTTATCAAATTAGGTTCAGAGTTCTTAGTTTAGTTGTATGGATCTGAGTTATGCAATATGAAATAATAAGTAATGTAACTAAGTTAAATTAATAAGTTAATAGAAAATATAAATAATAACATTAATTAACTAATATATTATATAAATTTATTGGTTTATTTGATTATATCATTAACCATTTCTTCATTTTTTGTTGTGAGGAGCTTACCATGAATCCCATAATTTCTGCTGCTTCCG

>YNTC_1

CGGCGGCTTATATAGGTGAATCCATGGAAGGTCATCATTGAGTAGCTTTACATCTAAATAAGAAAATAATGCTTTGAATCGCGTTTTATTTTTGAAATTTATTGCAATTAAAAATGAAGCCCATGATTCTTATTCTAATAAGAAATTCATGGGTAACTCAAGATACCGAACATCTAAGGTCCGATCCGATTCAAATAAGATGGCGATGGTTTCCATTATGGAAGAAACACATGTATATGTGATAGTAGATATTCAATAGTTATATATGGACTACCCATCTATATTATTTCATTACCCATATTACCGACTTTGTATAGATATAGATTCCACTTTATATGGGTTACGATATAAGCTCTTCTTTTTTTCGTCTGTGACTGTAATGTCTGGATTGAATATGAAGTCAAGCCTATGAATGCATATAGAGAAGATGAAGGAACGAAGAGTTGAAAGAATGAGTTCGGAACAAAAACAAAACAAAGAAATGGAAGAACTAGAACTCTACGGATTTACAAAGACTTGTATAGGGAATAAAAACGAGATATTGAAGTAGTTCGTATGATTCAGTAATATCAAATATCATCACTTATCAAATTAGGTTCAGAGTTCTTAGTTTAGTTGTATGGATCTGAGTTATGCAATATGAAATAATAAGTAATGTAACTAAGTTAAATTAATAAGTTAATAGAAAATATAAATAATAACATTAATTAACTAATATATTATATAAATTTATTGGTTTATTTGATTATATCATTAACCATTTCTTCATTTTTTGTTGTGAGGAGCTTACCATGAATCCCATAATTTCTGCTGCTTCCG

*ndhF-rpl32* (pairwise identity: 100%)


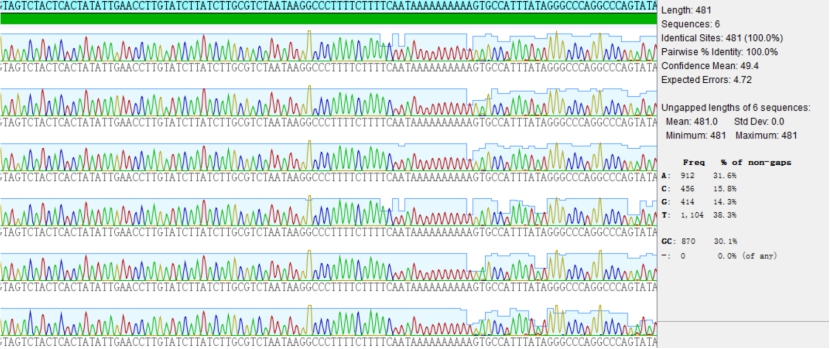


>NXQZS_1

CTAACCTATCTATGTTCAATCGTCGATATTAAGAGGTTCCATGCAATTGCAATAGCTTCTTATTGAATCTATCGAAGAATGGTGATTCAAGTCAATATTTGAAGGTTCAATAATTGTTACTAGCTTACCTTAGAGCTTGATATTGATAATAGTGAATCGATGGGGAAAATGGTAATCCCCATCTCGCAAATCATATAAGAATGTAGTCTACTCACTATATTGAACCTTGTATCTTATCTTGCGTCTAATAAGGCCCTTTTCTTTTCAATAAAAAAAAAAGTGCCATTTATAGGGCCCAGGCCCAGTATACGACTCAGAATTCTAAATTTATCCAATCATTTATTCATGTTGATTTAGATCATTTGAAAGGTTTCTTATCACATTATTATTTATTTTATTTATTCTTTGCTTTTTTTATTTTATTTTTTTTGTCGTACAAAAAAACCTTTTGAATAACCGGTGGAAATGGATTTAGCTAAAGAGAAAGCTTTTACCGCTGCCCAATACCCCTTTCTCCTCCAAATATTTCTACGAATACGCTTTTTTGATATAGAAGT

>GDSYD_1

CTAACCTATCTATGTTCAATCGTCGATATTAAGAGGTTCCATGCAATTGCAATAGCTTCTTATTGAATCTATCGAAGAATGGTGATTCAAGTCAATATTTGAAGGTTCAATAATTGTTACTAGCTTACCTTAGAGCTTGATATTGATAATAGTGAATCGATGGGGAAAATGGTAATCCCCATCTCGCAAATCATATAAGAATGTAGTCTACTCACTATATTGAACCTTGTATCTTATCTTGCGTCTAATAAGGCCCTTTTCTTTTCAATAAAAAAAAAAGTGCCATTTATAGGGCCCAGGCCCAGTATACGACTCAGAATTCTAAATTTATCCAATCATTTATTCATGTTGATTTAGATCATTTGAAAGGTTTCTTATCACATTATTATTTATTTTATTTATTCTTTGCTTTTTTTATTTTATTTTTTTTGTCGTACAAAAAAACCTTTTGAATAACCGGTGGAAATGGATTTAGCTAAAGAGAAAGCTTTTACCGCTGCCCAATACCCCTTTCTCCTCCAAATATTTCTACGAATACGCTTTTTTGATATAGAAGT

>NDXFS_1

CTAACCTATCTATGTTCAATCGTCGATATTAAGAGGTTCCATGCAATTGCAATAGCTTCTTATTGAATCTATCGAAGAATGGTGATTCAAGTCAATATTTGAAGGTTCAATAATTGTTACTAGCTTACCTTAGAGCTTGATATTGATAATAGTGAATCGATGGGGAAAATGGTAATCCCCATCTCGCAAATCATATAAGAATGTAGTCTACTCACTATATTGAACCTTGTATCTTATCTTGCGTCTAATAAGGCCCTTTTCTTTTCAATAAAAAAAAAAGTGCCATTTATAGGGCCCAGGCCCAGTATACGACTCAGAATTCTAAATTTATCCAATCATTTATTCATGTTGATTTAGATCATTTGAAAGGTTTCTTATCACATTATTATTTATTTTATTTATTCTTTGCTTTTTTTATTTTATTTTTTTTGTCGTACAAAAAAACCTTTTGAATAACCGGTGGAAATGGATTTAGCTAAAGAGAAAGCTTTTACCGCTGCCCAATACCCCTTTCTCCTCCAAATATTTCTACGAATACGCTTTTTTGATATAGAAGT

>TWYL_1

CTAACCTATCTATGTTCAATCGTCGATATTAAGAGGTTCCATGCAATTGCAATAGCTTCTTATTGAATCTATCGAAGAATGGTGATTCAAGTCAATATTTGAAGGTTCAATAATTGTTACTAGCTTACCTTAGAGCTTGATATTGATAATAGTGAATCGATGGGGAAAATGGTAATCCCCATCTCGCAAATCATATAAGAATGTAGTCTACTCACTATATTGAACCTTGTATCTTATCTTGCGTCTAATAAGGCCCTTTTCTTTTCAATAAAAAAAAAAGTGCCATTTATAGGGCCCAGGCCCAGTATACGACTCAGAATTCTAAATTTATCCAATCATTTATTCATGTTGATTTAGATCATTTGAAAGGTTTCTTATCACATTATTATTTATTTTATTTATTCTTTGCTTTTTTTATTTTATTTTTTTTGTCGTACAAAAAAACCTTTTGAATAACCGGTGGAAATGGATTTAGCTAAAGAGAAAGCTTTTACCGCTGCCCAATACCCCTTTCTCCTCCAAATATTTCTACGAATACGCTTTTTTGATATAGAAGT

>QYBSZ_1

CTAACCTATCTATGTTCAATCGTCGATATTAAGAGGTTCCATGCAATTGCAATAGCTTCTTATTGAATCTATCGAAGAATGGTGATTCAAGTCAATATTTGAAGGTTCAATAATTGTTACTAGCTTACCTTAGAGCTTGATATTGATAATAGTGAATCGATGGGGAAAATGGTAATCCCCATCTCGCAAATCATATAAGAATGTAGTCTACTCACTATATTGAACCTTGTATCTTATCTTGCGTCTAATAAGGCCCTTTTCTTTTCAATAAAAAAAAAAGTGCCATTTATAGGGCCCAGGCCCAGTATACGACTCAGAATTCTAAATTTATCCAATCATTTATTCATGTTGATTTAGATCATTTGAAAGGTTTCTTATCACATTATTATTTATTTTATTTATTCTTTGCTTTTTTTATTTTATTTTTTTTGTCGTACAAAAAAACCTTTTGAATAACCGGTGGAAATGGATTTAGCTAAAGAGAAAGCTTTTACCGCTGCCCAATACCCCTTTCTCCTCCAAATATTTCTACGAATACGCTTTTTTGATATAGAAGT

>YNTC_1

CTAACCTATCTATGTTCAATCGTCGATATTAAGAGGTTCCATGCAATTGCAATAGCTTCTTATTGAATCTATCGAAGAATGGTGATTCAAGTCAATATTTGAAGGTTCAATAATTGTTACTAGCTTACCTTAGAGCTTGATATTGATAATAGTGAATCGATGGGGAAAATGGTAATCCCCATCTCGCAAATCATATAAGAATGTAGTCTACTCACTATATTGAACCTTGTATCTTATCTTGCGTCTAATAAGGCCCTTTTCTTTTCAATAAAAAAAAAAGTGCCATTTATAGGGCCCAGGCCCAGTATACGACTCAGAATTCTAAATTTATCCAATCATTTATTCATGTTGATTTAGATCATTTGAAAGGTTTCTTATCACATTATTATTTATTTTATTTATTCTTTGCTTTTTTTATTTTATTTTTTTTGTCGTACAAAAAAACCTTTTGAATAACCGGTGGAAATGGATTTAGCTAAAGAGAAAGCTTTTACCGCTGCCCAATACCCCTTTCTCCTCCAAATATTTCTACGAATACGCTTTTTTGATATAGAAGT

*rpL14-rpL36* (pairwise identity: 100%)


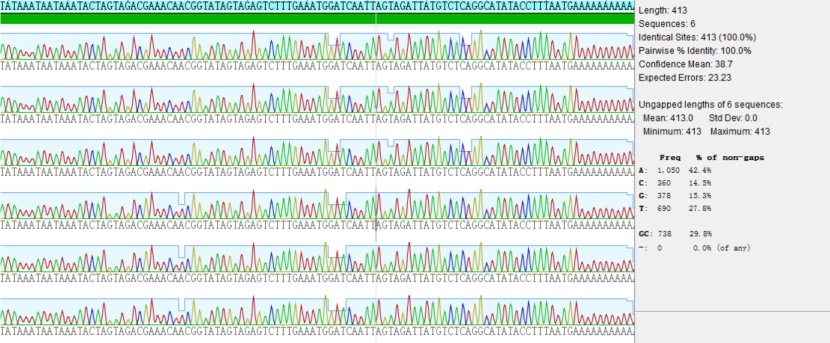


>NXQZS_1

CTCGTGATTGAGACAGTTAAATTTCACTAAGATAGTCTCATTAGCTCCCGAAGTCTTATAAATAATAAATACTAGTAGACGAAACAACGGTATAGTAGAGTCTTTGAAATGGATCAATTAGTAGATTATGTCTCAGGCATATACCTTTAATGAAAAAAAAAAATAAAAAAGAAAAACATGTTGATTATATCAAAAAAAAAAAATGATATTTCGTCATGGGTAAAGACACTATTGCCGATATAATAACTTATATAAAAAATGCTGACATGGATAAAAAAGGAACGGTTCGAATACCCTCCACTAATATTACCGAAAACATTGTTAAAATACTTCTACGAAAGGGTTTTATCGAAAATGTTAGGAAGCACCAGGAAAACAACAAATATTTCTTGGTTTTAACCCTACGACATAGAAGAAATAGGAAAGGAGCATATAGAAATATTTTAAAGCGTATCAGCAGACCAGGTCTGCGAATCTATTGCAACTCTCAACGAATTCCTAGGATTTCAGGCGGGATAGGGGTTGTAATTCTTTCTACTTCTAAAGGTATAATGACAGATCGAGAAGCTCGACTAAAAAGAATTGGAGGAGAAATTTTGTTTTATATATGGGGAGGTGATCCATCTGGTATGCGATACAAATTTGATACGTAACTTCCTATTTATGAAAAAGAGAGTAGAGGTGGGTTGT

>GDSYD_1

CTCGTGATTGAGACAGTTAAATTTCACTAAGATAGTCTCATTAGCTCCCGAAGTCTTATAAATAATAAATACTAGTAGACGAAACAACGGTATAGTAGAGTCTTTGAAATGGATCAATTAGTAGATTATGTCTCAGGCATATACCTTTAATGAAAAAAAAAAATAAAAAAGAAAAACATGTTGATTATATCAAAAAAAAAAAATGATATTTCGTCATGGGTAAAGACACTATTGCCGATATAATAACTTATATAAAAAATGCTGACATGGATAAAAAAGGAACGGTTCGAATACCCTCCACTAATATTACCGAAAACATTGTTAAAATACTTCTACGAAAGGGTTTTATCGAAAATGTTAGGAAGCACCAGGAAAACAACAAATATTTCTTGGTTTTAACCCTACGACATAGAAGAAATAGGAAAGGAGCATATAGAAATATTTTAAAGCGTATCAGCAGACCAGGTCTGCGAATCTATTGCAACTCTCAACGAATTCCTAGGATTTCAGGCGGGATAGGGGTTGTAATTCTTTCTACTTCTAAAGGTATAATGACAGATCGAGAAGCTCGACTAAAAAGAATTGGAGGAGAAATTTTGTTTTATATATGGGGAGGTGATCCATCTGGTATGCGATACAAATTTGATACGTAACTTCCTATTTATGAAAAAGAGAGTAGAGGTGGGTTGT

>NDXFS_1

CTCGTGATTGAGACAGTTAAATTTCACTAAGATAGTCTCATTAGCTCCCGAAGTCTTATAAATAATAAATACTAGTAGACGAAACAACGGTATAGTAGAGTCTTTGAAATGGATCAATTAGTAGATTATGTCTCAGGCATATACCTTTAATGAAAAAAAAAAATAAAAAAGAAAAACATGTTGATTATATCAAAAAAAAAAAATGATATTTCGTCATGGGTAAAGACACTATTGCCGATATAATAACTTATATAAAAAATGCTGACATGGATAAAAAAGGAACGGTTCGAATACCCTCCACTAATATTACCGAAAACATTGTTAAAATACTTCTACGAAAGGGTTTTATCGAAAATGTTAGGAAGCACCAGGAAAACAACAAATATTTCTTGGTTTTAACCCTACGACATAGAAGAAATAGGAAAGGAGCATATAGAAATATTTTAAAGCGTATCAGCAGACCAGGTCTGCGAATCTATTGCAACTCTCAACGAATTCCTAGGATTTCAGGCGGGATAGGGGTTGTAATTCTTTCTACTTCTAAAGGTATAATGACAGATCGAGAAGCTCGACTAAAAAGAATTGGAGGAGAAATTTTGTTTTATATATGGGGAGGTGATCCATCTGGTATGCGATACAAATTTGATACGTAACTTCCTATTTATGAAAAAGAGAGTAGAGGTGGGTTGT

>TWYL_1

CTCGTGATTGAGACAGTTAAATTTCACTAAGATAGTCTCATTAGCTCCCGAAGTCTTATAAATAATAAATACTAGTAGACGAAACAACGGTATAGTAGAGTCTTTGAAATGGATCAATTAGTAGATTATGTCTCAGGCATATACCTTTAATGAAAAAAAAAAATAAAAAAGAAAAACATGTTGATTATATCAAAAAAAAAAAATGATATTTCGTCATGGGTAAAGACACTATTGCCGATATAATAACTTATATAAAAAATGCTGACATGGATAAAAAAGGAACGGTTCGAATACCCTCCACTAATATTACCGAAAACATTGTTAAAATACTTCTACGAAAGGGTTTTATCGAAAATGTTAGGAAGCACCAGGAAAACAACAAATATTTCTTGGTTTTAACCCTACGACATAGAAGAAATAGGAAAGGAGCATATAGAAATATTTTAAAGCGTATCAGCAGACCAGGTCTGCGAATCTATTGCAACTCTCAACGAATTCCTAGGATTTCAGGCGGGATAGGGGTTGTAATTCTTTCTACTTCTAAAGGTATAATGACAGATCGAGAAGCTCGACTAAAAAGAATTGGAGGAGAAATTTTGTTTTATATATGGGGAGGTGATCCATCTGGTATGCGATACAAATTTGATACGTAACTTCCTATTTATGAAAAAGAGAGTAGAGGTGGGTTGT

>QYBSZ_1

CTCGTGATTGAGACAGTTAAATTTCACTAAGATAGTCTCATTAGCTCCCGAAGTCTTATAAATAATAAATACTAGTAGACGAAACAACGGTATAGTAGAGTCTTTGAAATGGATCAATTAGTAGATTATGTCTCAGGCATATACCTTTAATGAAAAAAAAAAATAAAAAAGAAAAACATGTTGATTATATCAAAAAAAAAAAATGATATTTCGTCATGGGTAAAGACACTATTGCCGATATAATAACTTATATAAAAAATGCTGACATGGATAAAAAAGGAACGGTTCGAATACCCTCCACTAATATTACCGAAAACATTGTTAAAATACTTCTACGAAAGGGTTTTATCGAAAATGTTAGGAAGCACCAGGAAAACAACAAATATTTCTTGGTTTTAACCCTACGACATAGAAGAAATAGGAAAGGAGCATATAGAAATATTTTAAAGCGTATCAGCAGACCAGGTCTGCGAATCTATTGCAACTCTCAACGAATTCCTAGGATTTCAGGCGGGATAGGGGTTGTAATTCTTTCTACTTCTAAAGGTATAATGACAGATCGAGAAGCTCGACTAAAAAGAATTGGAGGAGAAATTTTGTTTTATATATGGGGAGGTGATCCATCTGGTATGCGATACAAATTTGATACGTAACTTCCTATTTATGAAAAAGAGAGTAGAGGTGGGTTGT

>YNTC_1

CTCGTGATTGAGACAGTTAAATTTCACTAAGATAGTCTCATTAGCTCCCGAAGTCTTATAAATAATAAATACTAGTAGACGAAACAACGGTATAGTAGAGTCTTTGAAATGGATCAATTAGTAGATTATGTCTCAGGCATATACCTTTAATGAAAAAAAAAAATAAAAAAGAAAAACATGTTGATTATATCAAAAAAAAAAAATGATATTTCGTCATGGGTAAAGACACTATTGCCGATATAATAACTTATATAAAAAATGCTGACATGGATAAAAAAGGAACGGTTCGAATACCCTCCACTAATATTACCGAAAACATTGTTAAAATACTTCTACGAAAGGGTTTTATCGAAAATGTTAGGAAGCACCAGGAAAACAACAAATATTTCTTGGTTTTAACCCTACGACATAGAAGAAATAGGAAAGGAGCATATAGAAATATTTTAAAGCGTATCAGCAGACCAGGTCTGCGAATCTATTGCAACTCTCAACGAATTCCTAGGATTTCAGGCGGGATAGGGGTTGTAATTCTTTCTACTTCTAAAGGTATAATGACAGATCGAGAAGCTCGACTAAAAAGAATTGGAGGAGAAATTTTGTTTTATATATGGGGAGGTGATCCATCTGGTATGCGATACAAATTTGATACGTAACTTCCTATTTATGAAAAAGAGAGTAGAGGTGGGTTGT

*psbJ-petA* (pairwise identity: 99.6%，one Indel)


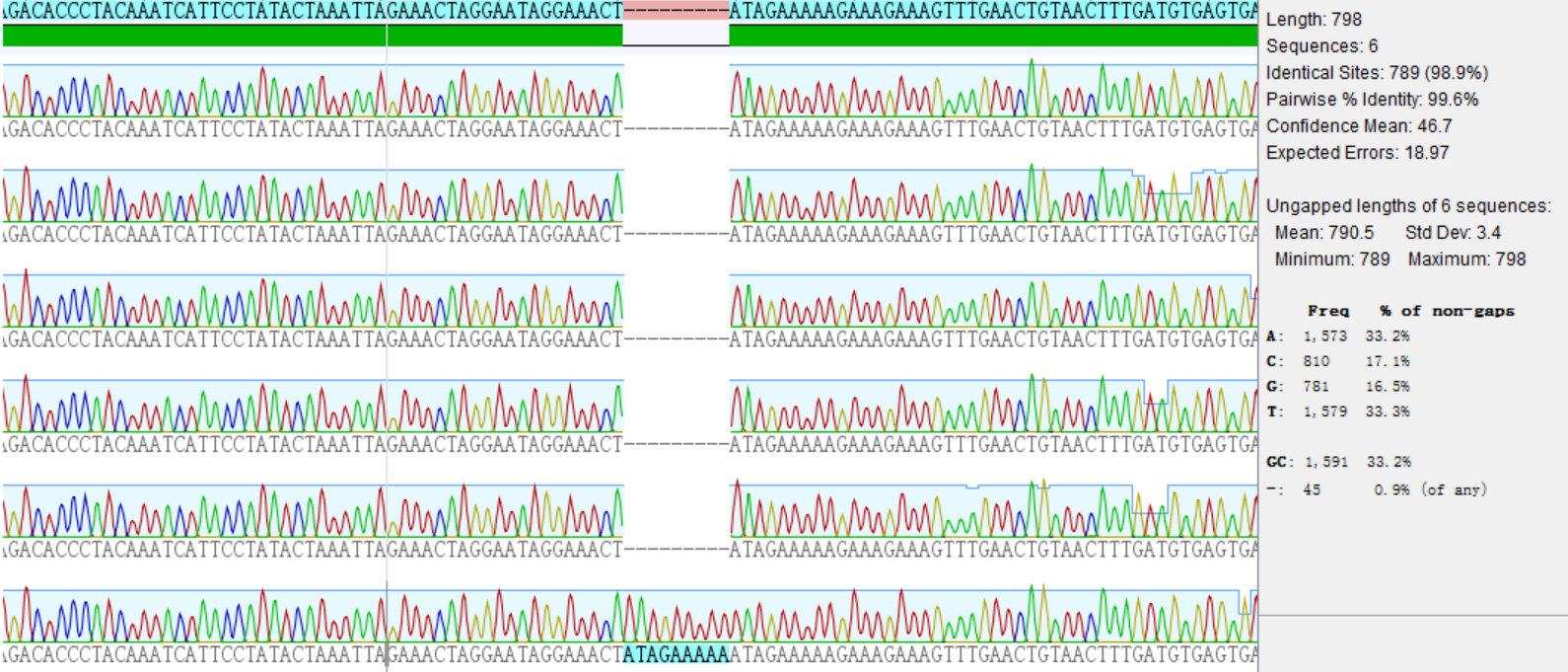


>NXQZS_1

CTTTTCTTCTACGGCTCATATTCCGGATTGGGATCATCCCTGTAATAATCGGATGAACCGGACTGTAGACATGAAAGAGTAAGAACTCAACAGGACCTCAAATCCATATAAGGAGGGGTCTGGTCCTGTTGAGTTCTTACTCTTCGACCGAGACCTTGACCCATAAGGGGTGGAAATTAATCCAGTCAACACAATCATAATACGTATACATGTATTAGACTAAATGAAAAAATGGATCATTTGATTCGTCCCCACAAATTACTTTACTACATTCCTTTATTTTTCATGATGTTTTTGAAACGCCGAATCCGCTGATTGATTCATAGTATCTATAGATATGGTGTGGACTTTTCCGAAGTAAAAATAAAGAAAGAAGGATCGCTTTTTTGAATGACACCATATGGATTTCTCCAGATGAGACACCCTACAAATCATTCCTATACTAAATTAGAAACTAGGAATAGGAAACTATAGAAAAAGAAAGAAAGTTTGAACTGTAACTTTGATGTGAGTGATGAGATAATAAGGTAAGGTATAATAAGATTATCAAATTAATAAGGATTTGTATATGTCCGAATTTAAATTTCCCCTTTTTGACCCGTAAACAGAACATGAAAAATCTTTTTGAGCGAGTCTTTTTTCTTTTTATAATATAAGTTAAAGTTAAGTTAATTGAATAAATT

>GDSYD_1

CTTTTCTTCTACGGCTCATATTCCGGATTGGGATCATCCCTGTAATAATCGGATGAACCGGACTGTAGACATGAAAGAGTAAGAACTCAACAGGACCTCAAATCCATATAAGGAGGGGTCTGGTCCTGTTGAGTTCTTACTCTTCGACCGAGACCTTGACCCATAAGGGGTGGAAATTAATCCAGTCAACACAATCATAATACGTATACATGTATTAGACTAAATGAAAAAATGGATCATTTGATTCGTCCCCACAAATTACTTTACTACATTCCTTTATTTTTCATGATGTTTTTGAAACGCCGAATCCGCTGATTGATTCATAGTATCTATAGATATGGTGTGGACTTTTCCGAAGTAAAAATAAAGAAAGAAGGATCGCTTTTTTGAATGACACCATATGGATTTCTCCAGATGAGACACCCTACAAATCATTCCTATACTAAATTAGAAACTAGGAATAGGAAACTATAGAAAAAGAAAGAAAGTTTGAACTGTAACTTTGATGTGAGTGATGAGATAATAAGGTAAGGTATAATAAGATTATCAAATTAATAAGGATTTGTATATGTCCGAATTTAAATTTCCCCTTTTTGACCCGTAAACAGAACATGAAAAATCTTTTTGAGCGAGTCTTTTTTCTTTTTATAATATAAGTTAAAGTTAAGTTAATTGAATAAATT

>NDXFS_1

CTTTTCTTCTACGGCTCATATTCCGGATTGGGATCATCCCTGTAATAATCGGATGAACCGGACTGTAGACATGAAAGAGTAAGAACTCAACAGGACCTCAAATCCATATAAGGAGGGGTCTGGTCCTGTTGAGTTCTTACTCTTCGACCGAGACCTTGACCCATAAGGGGTGGAAATTAATCCAGTCAACACAATCATAATACGTATACATGTATTAGACTAAATGAAAAAATGGATCATTTGATTCGTCCCCACAAATTACTTTACTACATTCCTTTATTTTTCATGATGTTTTTGAAACGCCGAATCCGCTGATTGATTCATAGTATCTATAGATATGGTGTGGACTTTTCCGAAGTAAAAATAAAGAAAGAAGGATCGCTTTTTTGAATGACACCATATGGATTTCTCCAGATGAGACACCCTACAAATCATTCCTATACTAAATTAGAAACTAGGAATAGGAAACTATAGAAAAAGAAAGAAAGTTTGAACTGTAACTTTGATGTGAGTGATGAGATAATAAGGTAAGGTATAATAAGATTATCAAATTAATAAGGATTTGTATATGTCCGAATTTAAATTTCCCCTTTTTGACCCGTAAACAGAACATGAAAAATCTTTTTGAGCGAGTCTTTTTTCTTTTTATAATATAAGTTAAAGTTAAGTTAATTGAATAAATT

>TWYL_1

CTTTTCTTCTACGGCTCATATTCCGGATTGGGATCATCCCTGTAATAATCGGATGAACCGGACTGTAGACATGAAAGAGTAAGAACTCAACAGGACCTCAAATCCATATAAGGAGGGGTCTGGTCCTGTTGAGTTCTTACTCTTCGACCGAGACCTTGACCCATAAGGGGTGGAAATTAATCCAGTCAACACAATCATAATACGTATACATGTATTAGACTAAATGAAAAAATGGATCATTTGATTCGTCCCCACAAATTACTTTACTACATTCCTTTATTTTTCATGATGTTTTTGAAACGCCGAATCCGCTGATTGATTCATAGTATCTATAGATATGGTGTGGACTTTTCCGAAGTAAAAATAAAGAAAGAAGGATCGCTTTTTTGAATGACACCATATGGATTTCTCCAGATGAGACACCCTACAAATCATTCCTATACTAAATTAGAAACTAGGAATAGGAAACTATAGAAAAAGAAAGAAAGTTTGAACTGTAACTTTGATGTGAGTGATGAGATAATAAGGTAAGGTATAATAAGATTATCAAATTAATAAGGATTTGTATATGTCCGAATTTAAATTTCCCCTTTTTGACCCGTAAACAGAACATGAAAAATCTTTTTGAGCGAGTCTTTTTTCTTTTTATAATATAAGTTAAAGTTAAGTTAATTGAATAAATT

>QYBSZ_1

CTTTTCTTCTACGGCTCATATTCCGGATTGGGATCATCCCTGTAATAATCGGATGAACCGGACTGTAGACATGAAAGAGTAAGAACTCAACAGGACCTCAAATCCATATAAGGAGGGGTCTGGTCCTGTTGAGTTCTTACTCTTCGACCGAGACCTTGACCCATAAGGGGTGGAAATTAATCCAGTCAACACAATCATAATACGTATACATGTATTAGACTAAATGAAAAAATGGATCATTTGATTCGTCCCCACAAATTACTTTACTACATTCCTTTATTTTTCATGATGTTTTTGAAACGCCGAATCCGCTGATTGATTCATAGTATCTATAGATATGGTGTGGACTTTTCCGAAGTAAAAATAAAGAAAGAAGGATCGCTTTTTTGAATGACACCATATGGATTTCTCCAGATGAGACACCCTACAAATCATTCCTATACTAAATTAGAAACTAGGAATAGGAAACTATAGAAAAAGAAAGAAAGTTTGAACTGTAACTTTGATGTGAGTGATGAGATAATAAGGTAAGGTATAATAAGATTATCAAATTAATAAGGATTTGTATATGTCCGAATTTAAATTTCCCCTTTTTGACCCGTAAACAGAACATGAAAAATCTTTTTGAGCGAGTCTTTTTTCTTTTTATAATATAAGTTAAAGTTAAGTTAATTGAATAAATT

>YNTC_1

CTTTTCTTCTACGGCTCATATTCCGGATTGGGATCATCCCTGTAATAATCGGATGAACCGGACTGTAGACATGAAAGAGTAAGAACTCAACAGGACCTCAAATCCATATAAGGAGGGGTCTGGTCCTGTTGAGTTCTTACTCTTCGACCGAGACCTTGACCCATAAGGGGTGGAAATTAATCCAGTCAACACAATCATAATACGTATACATGTATTAGACTAAATGAAAAAATGGATCATTTGATTCGTCCCCACAAATTACTTTACTACATTCCTTTATTTTTCATGATGTTTTTGAAACGCCGAATCCGCTGATTGATTCATAGTATCTATAGATATGGTGTGGACTTTTCCGAAGTAAAAATAAAGAAAGAAGGATCGCTTTTTTGAATGACACCATATGGATTTCTCCAGATGAGACACCCTACAAATCATTCCTATACTAAATTAGAAACTAGGAATAGGAAACTATAGAAAAAATAGAAAAAGAAAGAAAGTTTGAACTGTAACTTTGATGTGAGTGATGAGATAATAAGGTAAGGTATAATAAGATTATCAAATTAATAAGGATTTGTATATGTCCGAATTTAAATTTCCCCTTTTTGACCCGTAAACAGAACATGAAAAATCTTTTTGAGCGAGTCTTTTTTCTTTTTATAATATAAGTTAAAGTTAAGTTAATTGAATAAATT

*psaI-accD* (pairwise identity: 100%)


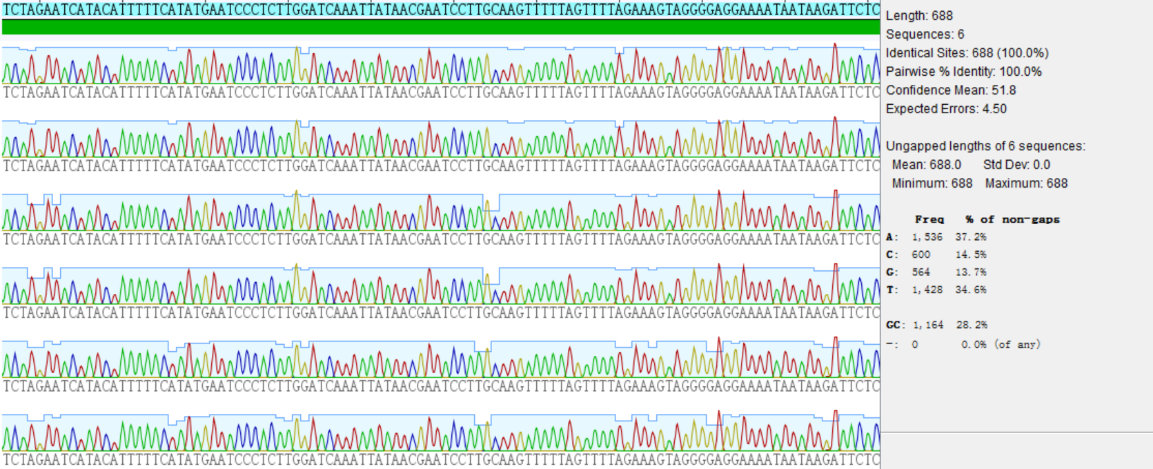


>NXQZS_1

ACTTCTAGAATCATACATTTTTCATATGAATCCCTCTTGGATCAAATTATAACGAATCCTTGCAAGTTTTTAGTTTTAGAAAGTAGGGGAGGAAAATAATAAGATTCTCATACATATATTTCCCGGAAAAGGATGCGTAGGTACACTAATTTCATATTTAGTTTAGATCTACATTCTTTGAACTCATATTCTAATAACTTAGAATATAATATACTTATAATTTAATATATAATTATAATATATTATTCTAAAAAAAAAAAAGATTGTTTATAACAAAGAGAAGTATCAATTAAATACCCCGAGGCACCGATTCTATGACAGATTTCAACTTCCCCTCTATTTTTGTGCCTTTAATAGGCCTATTATTT

>GDSYD_1

ACTTCTAGAATCATACATTTTTCATATGAATCCCTCTTGGATCAAATTATAACGAATCCTTGCAAGTTTTTAGTTTTAGAAAGTAGGGGAGGAAAATAATAAGATTCTCATACATATATTTCCCGGAAAAGGATGCGTAGGTACACTAATTTCATATTTAGTTTAGATCTACATTCTTTGAACTCATATTCTAATAACTTAGAATATAATATACTTATAATTTAATATATAATTATAATATATTATTCTAAAAAAAAAAAAGATTGTTTATAACAAAGAGAAGTATCAATTAAATACCCCGAGGCACCGATTCTATGACAGATTTCAACTTCCCCTCTATTTTTGTGCCTTTAATAGGCCTATTATTT

>NDXFS_1

ACTTCTAGAATCATACATTTTTCATATGAATCCCTCTTGGATCAAATTATAACGAATCCTTGCAAGTTTTTAGTTTTAGAAAGTAGGGGAGGAAAATAATAAGATTCTCATACATATATTTCCCGGAAAAGGATGCGTAGGTACACTAATTTCATATTTAGTTTAGATCTACATTCTTTGAACTCATATTCTAATAACTTAGAATATAATATACTTATAATTTAATATATAATTATAATATATTATTCTAAAAAAAAAAAAGATTGTTTATAACAAAGAGAAGTATCAATTAAATACCCCGAGGCACCGATTCTATGACAGATTTCAACTTCCCCTCTATTTTTGTGCCTTTAATAGGCCTATTATTT

>TWYL_1

ACTTCTAGAATCATACATTTTTCATATGAATCCCTCTTGGATCAAATTATAACGAATCCTTGCAAGTTTTTAGTTTTAGAAAGTAGGGGAGGAAAATAATAAGATTCTCATACATATATTTCCCGGAAAAGGATGCGTAGGTACACTAATTTCATATTTAGTTTAGATCTACATTCTTTGAACTCATATTCTAATAACTTAGAATATAATATACTTATAATTTAATATATAATTATAATATATTATTCTAAAAAAAAAAAAGATTGTTTATAACAAAGAGAAGTATCAATTAAATACCCCGAGGCACCGATTCTATGACAGATTTCAACTTCCCCTCTATTTTTGTGCCTTTAATAGGCCTATTATTT

>QYBSZ_1

ACTTCTAGAATCATACATTTTTCATATGAATCCCTCTTGGATCAAATTATAACGAATCCTTGCAAGTTTTTAGTTTTAGAAAGTAGGGGAGGAAAATAATAAGATTCTCATACATATATTTCCCGGAAAAGGATGCGTAGGTACACTAATTTCATATTTAGTTTAGATCTACATTCTTTGAACTCATATTCTAATAACTTAGAATATAATATACTTATAATTTAATATATAATTATAATATATTATTCTAAAAAAAAAAAAGATTGTTTATAACAAAGAGAAGTATCAATTAAATACCCCGAGGCACCGATTCTATGACAGATTTCAACTTCCCCTCTATTTTTGTGCCTTTAATAGGCCTATTATTT

>YNTC_1

ACTTCTAGAATCATACATTTTTCATATGAATCCCTCTTGGATCAAATTATAACGAATCCTTGCAAGTTTTTAGTTTTAGAAAGTAGGGGAGGAAAATAATAAGATTCTCATACATATATTTCCCGGAAAAGGATGCGTAGGTACACTAATTTCATATTTAGTTTAGATCTACATTCTTTGAACTCATATTCTAATAACTTAGAATATAATATACTTATAATTTAATATATAATTATAATATATTATTCTAAAAAAAAAAAAGATTGTTTATAACAAAGAGAAGTATCAATTAAATACCCCGAGGCACCGATTCTATGACAGATTTCAACTTCCCCTCTATTTTTGTGCCTTTAATAGGCCTATTATTT

*trnT-trnF* (pairwise identity: 100%)


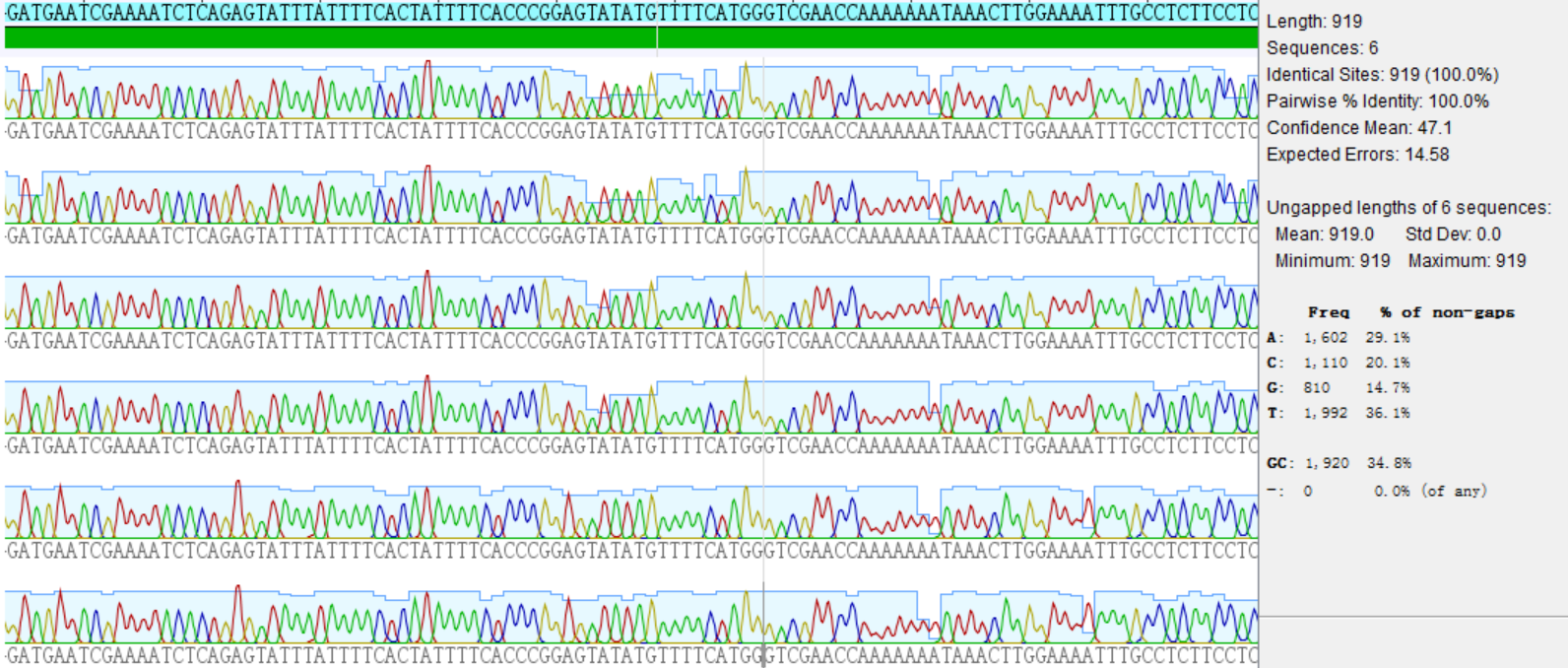


>NXQZS_1

GGGGATATGGCGAAATTGGTAGACGCTACGGACTTGATTGAATTGAGCCTTGGTATGGAAACTTACCAAGTGGTAACTTCCAAATTCAGAGAAACCCTGGAATTAAAAATGGGTAATCCTGAGCCAAATCCTGTTTAAAGAAAACAAGGGTTCCTTTCCTAGAAAGCGAGAATCAAAAAAGGATAGGTGCAGAGACTCAATGGAAGCTGTTCCAACGAATGGGGTTGAGGGTTGGTAGAAGAATCTATCCATCGGAACTCCGGGGGGATGATCCCATGTACTGAAATATCAAACGATTAATCACAACCCGAATCCTTTTTTTTATTTTATTATTTATTACTTTTAATATTTGATAATAAATTCTATTAATAATTAGTTCATAACTCTTGTGTTGTGAATCGATCCAATTTGAATGAAGAATCAAATATTCCGTGATGAAATCATTCACTCCAGAATATGAGGCTGGGATAAAAATTACCGATCGAACGAGAATAAAGATAGAGTCCCATTCTACCTGTCAATGCCGACAACAATGCAATTTGTTTGTAGTAGAAGGAAAATCCGTCGACTTTAGAAATCGTGAGGGTTCGAGTCCCTCTATCCCCAATAAATGCCTAGTTTACGACCTAAGCCTTTATCCTCTTTTTGCTTTTCGCCTGCGGTTCCAAATTAGATATGATATG

>GDSYD_1

GGGGATATGGCGAAATTGGTAGACGCTACGGACTTGATTGAATTGAGCCTTGGTATGGAAACTTACCAAGTGGTAACTTCCAAATTCAGAGAAACCCTGGAATTAAAAATGGGTAATCCTGAGCCAAATCCTGTTTAAAGAAAACAAGGGTTCCTTTCCTAGAAAGCGAGAATCAAAAAAGGATAGGTGCAGAGACTCAATGGAAGCTGTTCCAACGAATGGGGTTGAGGGTTGGTAGAAGAATCTATCCATCGGAACTCCGGGGGGATGATCCCATGTACTGAAATATCAAACGATTAATCACAACCCGAATCCTTTTTTTTATTTTATTATTTATTACTTTTAATATTTGATAATAAATTCTATTAATAATTAGTTCATAACTCTTGTGTTGTGAATCGATCCAATTTGAATGAAGAATCAAATATTCCGTGATGAAATCATTCACTCCAGAATATGAGGCTGGGATAAAAATTACCGATCGAACGAGAATAAAGATAGAGTCCCATTCTACCTGTCAATGCCGACAACAATGCAATTTGTTTGTAGTAGAAGGAAAATCCGTCGACTTTAGAAATCGTGAGGGTTCGAGTCCCTCTATCCCCAATAAATGCCTAGTTTACGACCTAAGCCTTTATCCTCTTTTTGCTTTTCGCCTGCGGTTCCAAATTAGATATGATATG

>NDXFS_1

GGGGATATGGCGAAATTGGTAGACGCTACGGACTTGATTGAATTGAGCCTTGGTATGGAAACTTACCAAGTGGTAACTTCCAAATTCAGAGAAACCCTGGAATTAAAAATGGGTAATCCTGAGCCAAATCCTGTTTAAAGAAAACAAGGGTTCCTTTCCTAGAAAGCGAGAATCAAAAAAGGATAGGTGCAGAGACTCAATGGAAGCTGTTCCAACGAATGGGGTTGAGGGTTGGTAGAAGAATCTATCCATCGGAACTCCGGGGGGATGATCCCATGTACTGAAATATCAAACGATTAATCACAACCCGAATCCTTTTTTTTATTTTATTATTTATTACTTTTAATATTTGATAATAAATTCTATTAATAATTAGTTCATAACTCTTGTGTTGTGAATCGATCCAATTTGAATGAAGAATCAAATATTCCGTGATGAAATCATTCACTCCAGAATATGAGGCTGGGATAAAAATTACCGATCGAACGAGAATAAAGATAGAGTCCCATTCTACCTGTCAATGCCGACAACAATGCAATTTGTTTGTAGTAGAAGGAAAATCCGTCGACTTTAGAAATCGTGAGGGTTCGAGTCCCTCTATCCCCAATAAATGCCTAGTTTACGACCTAAGCCTTTATCCTCTTTTTGCTTTTCGCCTGCGGTTCCAAATTAGATATGATATG

>TWYL_1

GGGGATATGGCGAAATTGGTAGACGCTACGGACTTGATTGAATTGAGCCTTGGTATGGAAACTTACCAAGTGGTAACTTCCAAATTCAGAGAAACCCTGGAATTAAAAATGGGTAATCCTGAGCCAAATCCTGTTTAAAGAAAACAAGGGTTCCTTTCCTAGAAAGCGAGAATCAAAAAAGGATAGGTGCAGAGACTCAATGGAAGCTGTTCCAACGAATGGGGTTGAGGGTTGGTAGAAGAATCTATCCATCGGAACTCCGGGGGGATGATCCCATGTACTGAAATATCAAACGATTAATCACAACCCGAATCCTTTTTTTTATTTTATTATTTATTACTTTTAATATTTGATAATAAATTCTATTAATAATTAGTTCATAACTCTTGTGTTGTGAATCGATCCAATTTGAATGAAGAATCAAATATTCCGTGATGAAATCATTCACTCCAGAATATGAGGCTGGGATAAAAATTACCGATCGAACGAGAATAAAGATAGAGTCCCATTCTACCTGTCAATGCCGACAACAATGCAATTTGTTTGTAGTAGAAGGAAAATCCGTCGACTTTAGAAATCGTGAGGGTTCGAGTCCCTCTATCCCCAATAAATGCCTAGTTTACGACCTAAGCCTTTATCCTCTTTTTGCTTTTCGCCTGCGGTTCCAAATTAGATATGATATG

>QYBSZ_1

GGGGATATGGCGAAATTGGTAGACGCTACGGACTTGATTGAATTGAGCCTTGGTATGGAAACTTACCAAGTGGTAACTTCCAAATTCAGAGAAACCCTGGAATTAAAAATGGGTAATCCTGAGCCAAATCCTGTTTAAAGAAAACAAGGGTTCCTTTCCTAGAAAGCGAGAATCAAAAAAGGATAGGTGCAGAGACTCAATGGAAGCTGTTCCAACGAATGGGGTTGAGGGTTGGTAGAAGAATCTATCCATCGGAACTCCGGGGGGATGATCCCATGTACTGAAATATCAAACGATTAATCACAACCCGAATCCTTTTTTTTATTTTATTATTTATTACTTTTAATATTTGATAATAAATTCTATTAATAATTAGTTCATAACTCTTGTGTTGTGAATCGATCCAATTTGAATGAAGAATCAAATATTCCGTGATGAAATCATTCACTCCAGAATATGAGGCTGGGATAAAAATTACCGATCGAACGAGAATAAAGATAGAGTCCCATTCTACCTGTCAATGCCGACAACAATGCAATTTGTTTGTAGTAGAAGGAAAATCCGTCGACTTTAGAAATCGTGAGGGTTCGAGTCCCTCTATCCCCAATAAATGCCTAGTTTACGACCTAAGCCTTTATCCTCTTTTTGCTTTTCGCCTGCGGTTCCAAATTAGATATGATATG

>YNTC_1

GGGGATATGGCGAAATTGGTAGACGCTACGGACTTGATTGAATTGAGCCTTGGTATGGAAACTTACCAAGTGGTAACTTCCAAATTCAGAGAAACCCTGGAATTAAAAATGGGTAATCCTGAGCCAAATCCTGTTTAAAGAAAACAAGGGTTCCTTTCCTAGAAAGCGAGAATCAAAAAAGGATAGGTGCAGAGACTCAATGGAAGCTGTTCCAACGAATGGGGTTGAGGGTTGGTAGAAGAATCTATCCATCGGAACTCCGGGGGGATGATCCCATGTACTGAAATATCAAACGATTAATCACAACCCGAATCCTTTTTTTTATTTTATTATTTATTACTTTTAATATTTGATAATAAATTCTATTAATAATTAGTTCATAACTCTTGTGTTGTGAATCGATCCAATTTGAATGAAGAATCAAATATTCCGTGATGAAATCATTCACTCCAGAATATGAGGCTGGGATAAAAATTACCGATCGAACGAGAATAAAGATAGAGTCCCATTCTACCTGTCAATGCCGACAACAATGCAATTTGTTTGTAGTAGAAGGAAAATCCGTCGACTTTAGAAATCGTGAGGGTTCGAGTCCCTCTATCCCCAATAAATGCCTAGTTTACGACCTAAGCCTTTATCCTCTTTTTGCTTTTCGCCTGCGGTTCCAAATTAGATATGATATG

*3’rps16-5’trnK* (pairwise identity: 99.9%, one SNP)


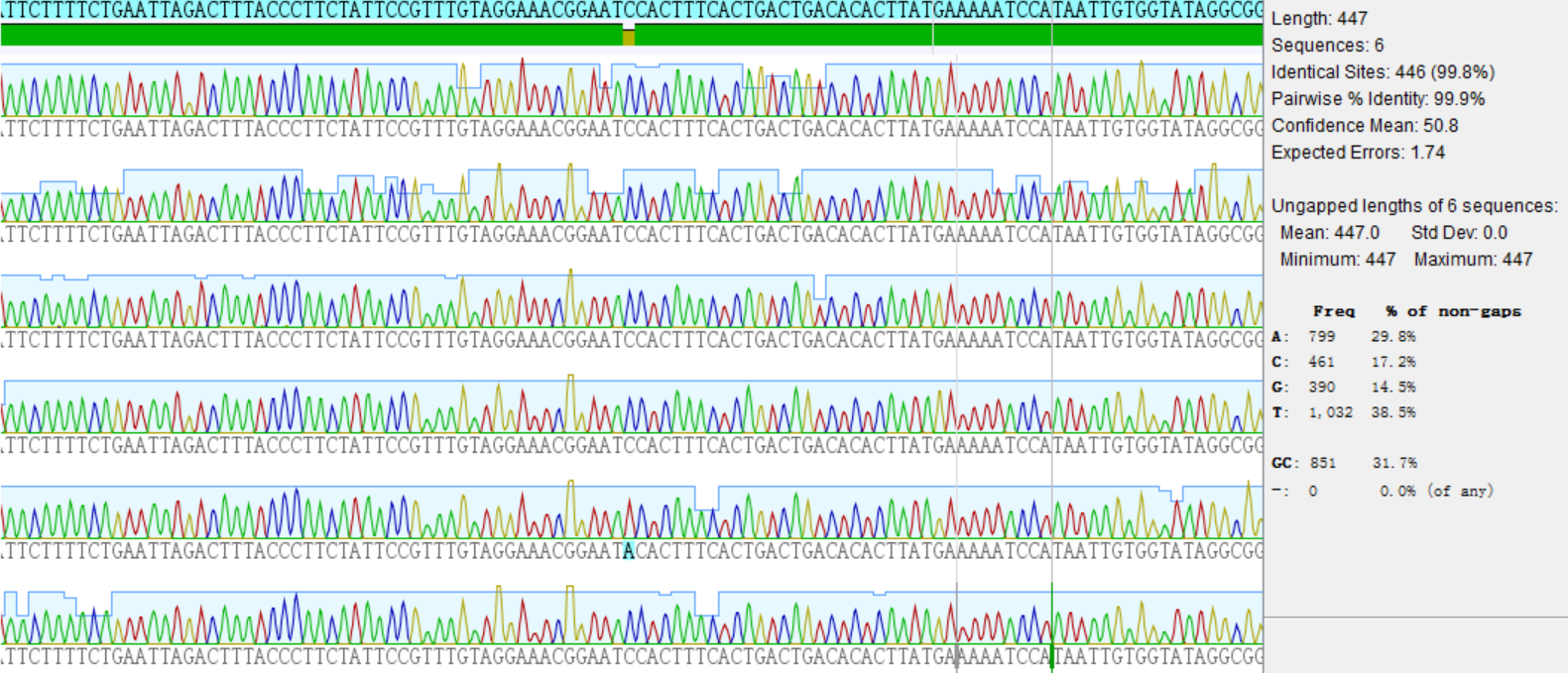


>NXQZS_1

TCCTTCCGTCCCAGATCAATTCCATCTTAACTTAACAAATATTATTTGTTCTCTTTTCAAAATCTTAATAATCTTCTACTTATTTGAAGTTTAAGAGGTTCATGTTGGATACGATGTAATCATAATCCAGCCGTTATTTATAGTTTTTTCTTTCTAGTTTGGTTTTTCATGATTCTTTTCTGAATTAGACTTTACCCTTCTATTCCGTTTGTAGGAAACGGAATCCACTTTCACTGACTGACACACTTATGAAAAATCCATAATTGTGGTATAGGCGGGGCATAAACATGGATCTTTCTTTATGCTTAAACCCATCAAATTCTTGTTCCTGCACGATCCATTCTGAGTCGAAATGTGAAGTTGAAGTGAAAGAAAGAAAACTCTTCTATCTTAGAACTTCTAATTAATGGTAAAGCAGATATGGTAATCGTATTTTCATGTAAAAATTATCCCAATTGCTAATTCC

>GDSYD_1

TCCTTCCGTCCCAGATCAATTCCATCTTAACTTAACAAATATTATTTGTTCTCTTTTCAAAATCTTAATAATCTTCTACTTATTTGAAGTTTAAGAGGTTCATGTTGGATACGATGTAATCATAATCCAGCCGTTATTTATAGTTTTTTCTTTCTAGTTTGGTTTTTCATGATTCTTTTCTGAATTAGACTTTACCCTTCTATTCCGTTTGTAGGAAACGGAATCCACTTTCACTGACTGACACACTTATGAAAAATCCATAATTGTGGTATAGGCGGGGCATAAACATGGATCTTTCTTTATGCTTAAACCCATCAAATTCTTGTTCCTGCACGATCCATTCTGAGTCGAAATGTGAAGTTGAAGTGAAAGAAAGAAAACTCTTCTATCTTAGAACTTCTAATTAATGGTAAAGCAGATATGGTAATCGTATTTTCATGTAAAAATTATCCCAATTGCTAATTCC

>NDXFS_1

TCCTTCCGTCCCAGATCAATTCCATCTTAACTTAACAAATATTATTTGTTCTCTTTTCAAAATCTTAATAATCTTCTACTTATTTGAAGTTTAAGAGGTTCATGTTGGATACGATGTAATCATAATCCAGCCGTTATTTATAGTTTTTTCTTTCTAGTTTGGTTTTTCATGATTCTTTTCTGAATTAGACTTTACCCTTCTATTCCGTTTGTAGGAAACGGAATCCACTTTCACTGACTGACACACTTATGAAAAATCCATAATTGTGGTATAGGCGGGGCATAAACATGGATCTTTCTTTATGCTTAAACCCATCAAATTCTTGTTCCTGCACGATCCATTCTGAGTCGAAATGTGAAGTTGAAGTGAAAGAAAGAAAACTCTTCTATCTTAGAACTTCTAATTAATGGTAAAGCAGATATGGTAATCGTATTTTCATGTAAAAATTATCCCAATTGCTAATTCC

>TWYL_1

TCCTTCCGTCCCAGATCAATTCCATCTTAACTTAACAAATATTATTTGTTCTCTTTTCAAAATCTTAATAATCTTCTACTTATTTGAAGTTTAAGAGGTTCATGTTGGATACGATGTAATCATAATCCAGCCGTTATTTATAGTTTTTTCTTTCTAGTTTGGTTTTTCATGATTCTTTTCTGAATTAGACTTTACCCTTCTATTCCGTTTGTAGGAAACGGAATCCACTTTCACTGACTGACACACTTATGAAAAATCCATAATTGTGGTATAGGCGGGGCATAAACATGGATCTTTCTTTATGCTTAAACCCATCAAATTCTTGTTCCTGCACGATCCATTCTGAGTCGAAATGTGAAGTTGAAGTGAAAGAAAGAAAACTCTTCTATCTTAGAACTTCTAATTAATGGTAAAGCAGATATGGTAATCGTATTTTCATGTAAAAATTATCCCAATTGCTAATTCC

>QYBSZ_1

TCCTTCCGTCCCAGATCAATTCCATCTTAACTTAACAAATATTATTTGTTCTCTTTTCAAAATCTTAATAATCTTCTACTTATTTGAAGTTTAAGAGGTTCATGTTGGATACGATGTAATCATAATCCAGCCGTTATTTATAGTTTTTTCTTTCTAGTTTGGTTTTTCATGATTCTTTTCTGAATTAGACTTTACCCTTCTATTCCGTTTGTAGGAAACGGAATACACTTTCACTGACTGACACACTTATGAAAAATCCATAATTGTGGTATAGGCGGGGCATAAACATGGATCTTTCTTTATGCTTAAACCCATCAAATTCTTGTTCCTGCACGATCCATTCTGAGTCGAAATGTGAAGTTGAAGTGAAAGAAAGAAAACTCTTCTATCTTAGAACTTCTAATTAATGGTAAAGCAGATATGGTAATCGTATTTTCATGTAAAAATTATCCCAATTGCTAATTCC

>YNTC_1

TCCTTCCGTCCCAGATCAATTCCATCTTAACTTAACAAATATTATTTGTTCTCTTTTCAAAATCTTAATAATCTTCTACTTATTTGAAGTTTAAGAGGTTCATGTTGGATACGATGTAATCATAATCCAGCCGTTATTTATAGTTTTTTCTTTCTAGTTTGGTTTTTCATGATTCTTTTCTGAATTAGACTTTACCCTTCTATTCCGTTTGTAGGAAACGGAATCCACTTTCACTGACTGACACACTTATGAAAAATCCATAATTGTGGTATAGGCGGGGCATAAACATGGATCTTTCTTTATGCTTAAACCCATCAAATTCTTGTTCCTGCACGATCCATTCTGAGTCGAAATGTGAAGTTGAAGTGAAAGAAAGAAAACTCTTCTATCTTAGAACTTCTAATTAATGGTAAAGCAGATATGGTAATCGTATTTTCATGTAAAAATTATCCCAATTGCTAATTCC

*matK-trnK* (pairwise identity: 100%)


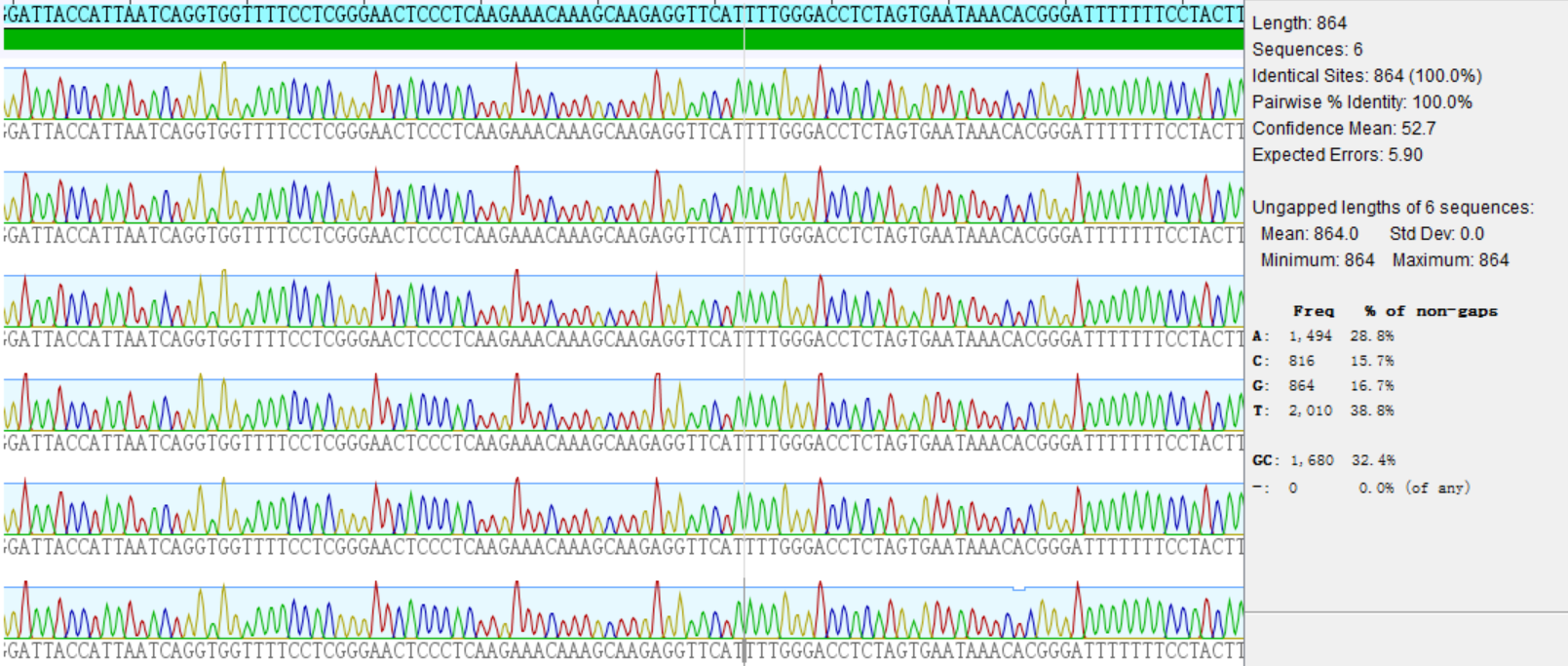


>NXQZS_1

CTATGATCTTTTACACATTTGGATGAAGCGGATTCGTCTATACCATCGGTAGAGTTTGTAAGACCACGACTGATCCTGAAAGGTAAGGGAATGAATGGAAAAAACAGCATGTCGTATCAATGGATAATCCTGAGAATCCTTCTGGTCATATCAGATCGGTAAAAATGTCCTTTTGATTCTTAGTTGGACCAGTTCAAAATTCATTCATAGTTGGGTCAAGTGAATAAATGGATAGAGCTATATGGCTCCAATTATAAGGAAAAACCAAAAGCAACGAGCTTCCGTTCTTATCTTAATTCGAACGAATACCCGATCTAATTAGACGTTAAAAATATATTAGTGCCTGAGGCGGGAAAGGGCTCTCCTACGAGTGAATCATTGATTCCTTAAAAAAAATCCTAACTATTCACTTCACTATTCTCCATTAGTTACTGGAGTGTAACCGAATGTGTATAAGAAACGGTGTACTGATAAACAAAATTAATTAATTCCAAAGTCAGAAGAGCGATCAGGTTGCAAAAATAAAGGATTTCTAATACACAATCTCTTGTAACTATACCCAAACACGAACGAGTTGGATGGAAAAAGAGAGGATGCGTTGATTAGACGTCTTGTCTCCGGGGTATCCGTTTTTACGATATACCTTGATAGGACCATATCGCACTATGTATTATTTGATAACCCAAGAAATCCTACCCCGCATGCGGTTAAAATTTAATTTCAAATGGATAAATTGCAATACGAATTGCAAGGTTATTTACAAATAGATAGATACCGGAAACAGCGCTTCCTATATCCACTTCTTTTTCGGGAATATATCTATGCACTTGCTCATGATCATG

>GDSYD_1

CTATGATCTTTTACACATTTGGATGAAGCGGATTCGTCTATACCATCGGTAGAGTTTGTAAGACCACGACTGATCCTGAAAGGTAAGGGAATGAATGGAAAAAACAGCATGTCGTATCAATGGATAATCCTGAGAATCCTTCTGGTCATATCAGATCGGTAAAAATGTCCTTTTGATTCTTAGTTGGACCAGTTCAAAATTCATTCATAGTTGGGTCAAGTGAATAAATGGATAGAGCTATATGGCTCCAATTATAAGGAAAAACCAAAAGCAACGAGCTTCCGTTCTTATCTTAATTCGAACGAATACCCGATCTAATTAGACGTTAAAAATATATTAGTGCCTGAGGCGGGAAAGGGCTCTCCTACGAGTGAATCATTGATTCCTTAAAAAAAATCCTAACTATTCACTTCACTATTCTCCATTAGTTACTGGAGTGTAACCGAATGTGTATAAGAAACGGTGTACTGATAAACAAAATTAATTAATTCCAAAGTCAGAAGAGCGATCAGGTTGCAAAAATAAAGGATTTCTAATACACAATCTCTTGTAACTATACCCAAACACGAACGAGTTGGATGGAAAAAGAGAGGATGCGTTGATTAGACGTCTTGTCTCCGGGGTATCCGTTTTTACGATATACCTTGATAGGACCATATCGCACTATGTATTATTTGATAACCCAAGAAATCCTACCCCGCATGCGGTTAAAATTTAATTTCAAATGGATAAATTGCAATACGAATTGCAAGGTTATTTACAAATAGATAGATACCGGAAACAGCGCTTCCTATATCCACTTCTTTTTCGGGAATATATCTATGCACTTGCTCATGATCATG

>NDXFS_1

CTATGATCTTTTACACATTTGGATGAAGCGGATTCGTCTATACCATCGGTAGAGTTTGTAAGACCACGACTGATCCTGAAAGGTAAGGGAATGAATGGAAAAAACAGCATGTCGTATCAATGGATAATCCTGAGAATCCTTCTGGTCATATCAGATCGGTAAAAATGTCCTTTTGATTCTTAGTTGGACCAGTTCAAAATTCATTCATAGTTGGGTCAAGTGAATAAATGGATAGAGCTATATGGCTCCAATTATAAGGAAAAACCAAAAGCAACGAGCTTCCGTTCTTATCTTAATTCGAACGAATACCCGATCTAATTAGACGTTAAAAATATATTAGTGCCTGAGGCGGGAAAGGGCTCTCCTACGAGTGAATCATTGATTCCTTAAAAAAAATCCTAACTATTCACTTCACTATTCTCCATTAGTTACTGGAGTGTAACCGAATGTGTATAAGAAACGGTGTACTGATAAACAAAATTAATTAATTCCAAAGTCAGAAGAGCGATCAGGTTGCAAAAATAAAGGATTTCTAATACACAATCTCTTGTAACTATACCCAAACACGAACGAGTTGGATGGAAAAAGAGAGGATGCGTTGATTAGACGTCTTGTCTCCGGGGTATCCGTTTTTACGATATACCTTGATAGGACCATATCGCACTATGTATTATTTGATAACCCAAGAAATCCTACCCCGCATGCGGTTAAAATTTAATTTCAAATGGATAAATTGCAATACGAATTGCAAGGTTATTTACAAATAGATAGATACCGGAAACAGCGCTTCCTATATCCACTTCTTTTTCGGGAATATATCTATGCACTTGCTCATGATCATG

>TWYL_1

CTATGATCTTTTACACATTTGGATGAAGCGGATTCGTCTATACCATCGGTAGAGTTTGTAAGACCACGACTGATCCTGAAAGGTAAGGGAATGAATGGAAAAAACAGCATGTCGTATCAATGGATAATCCTGAGAATCCTTCTGGTCATATCAGATCGGTAAAAATGTCCTTTTGATTCTTAGTTGGACCAGTTCAAAATTCATTCATAGTTGGGTCAAGTGAATAAATGGATAGAGCTATATGGCTCCAATTATAAGGAAAAACCAAAAGCAACGAGCTTCCGTTCTTATCTTAATTCGAACGAATACCCGATCTAATTAGACGTTAAAAATATATTAGTGCCTGAGGCGGGAAAGGGCTCTCCTACGAGTGAATCATTGATTCCTTAAAAAAAATCCTAACTATTCACTTCACTATTCTCCATTAGTTACTGGAGTGTAACCGAATGTGTATAAGAAACGGTGTACTGATAAACAAAATTAATTAATTCCAAAGTCAGAAGAGCGATCAGGTTGCAAAAATAAAGGATTTCTAATACACAATCTCTTGTAACTATACCCAAACACGAACGAGTTGGATGGAAAAAGAGAGGATGCGTTGATTAGACGTCTTGTCTCCGGGGTATCCGTTTTTACGATATACCTTGATAGGACCATATCGCACTATGTATTATTTGATAACCCAAGAAATCCTACCCCGCATGCGGTTAAAATTTAATTTCAAATGGATAAATTGCAATACGAATTGCAAGGTTATTTACAAATAGATAGATACCGGAAACAGCGCTTCCTATATCCACTTCTTTTTCGGGAATATATCTATGCACTTGCTCATGATCATG

>QYBSZ_1

CTATGATCTTTTACACATTTGGATGAAGCGGATTCGTCTATACCATCGGTAGAGTTTGTAAGACCACGACTGATCCTGAAAGGTAAGGGAATGAATGGAAAAAACAGCATGTCGTATCAATGGATAATCCTGAGAATCCTTCTGGTCATATCAGATCGGTAAAAATGTCCTTTTGATTCTTAGTTGGACCAGTTCAAAATTCATTCATAGTTGGGTCAAGTGAATAAATGGATAGAGCTATATGGCTCCAATTATAAGGAAAAACCAAAAGCAACGAGCTTCCGTTCTTATCTTAATTCGAACGAATACCCGATCTAATTAGACGTTAAAAATATATTAGTGCCTGAGGCGGGAAAGGGCTCTCCTACGAGTGAATCATTGATTCCTTAAAAAAAATCCTAACTATTCACTTCACTATTCTCCATTAGTTACTGGAGTGTAACCGAATGTGTATAAGAAACGGTGTACTGATAAACAAAATTAATTAATTCCAAAGTCAGAAGAGCGATCAGGTTGCAAAAATAAAGGATTTCTAATACACAATCTCTTGTAACTATACCCAAACACGAACGAGTTGGATGGAAAAAGAGAGGATGCGTTGATTAGACGTCTTGTCTCCGGGGTATCCGTTTTTACGATATACCTTGATAGGACCATATCGCACTATGTATTATTTGATAACCCAAGAAATCCTACCCCGCATGCGGTTAAAATTTAATTTCAAATGGATAAATTGCAATACGAATTGCAAGGTTATTTACAAATAGATAGATACCGGAAACAGCGCTTCCTATATCCACTTCTTTTTCGGGAATATATCTATGCACTTGCTCATGATCATG

>YNTC_1

CTATGATCTTTTACACATTTGGATGAAGCGGATTCGTCTATACCATCGGTAGAGTTTGTAAGACCACGACTGATCCTGAAAGGTAAGGGAATGAATGGAAAAAACAGCATGTCGTATCAATGGATAATCCTGAGAATCCTTCTGGTCATATCAGATCGGTAAAAATGTCCTTTTGATTCTTAGTTGGACCAGTTCAAAATTCATTCATAGTTGGGTCAAGTGAATAAATGGATAGAGCTATATGGCTCCAATTATAAGGAAAAACCAAAAGCAACGAGCTTCCGTTCTTATCTTAATTCGAACGAATACCCGATCTAATTAGACGTTAAAAATATATTAGTGCCTGAGGCGGGAAAGGGCTCTCCTACGAGTGAATCATTGATTCCTTAAAAAAAATCCTAACTATTCACTTCACTATTCTCCATTAGTTACTGGAGTGTAACCGAATGTGTATAAGAAACGGTGTACTGATAAACAAAATTAATTAATTCCAAAGTCAGAAGAGCGATCAGGTTGCAAAAATAAAGGATTTCTAATACACAATCTCTTGTAACTATACCCAAACACGAACGAGTTGGATGGAAAAAGAGAGGATGCGTTGATTAGACGTCTTGTCTCCGGGGTATCCGTTTTTACGATATACCTTGATAGGACCATATCGCACTATGTATTATTTGATAACCCAAGAAATCCTACCCCGCATGCGGTTAAAATTTAATTTCAAATGGATAAATTGCAATACGAATTGCAAGGTTATTTACAAATAGATAGATACCGGAAACAGCGCTTCCTATATCCACTTCTTTTTCGGGAATATATCTATGCACTTGCTCATGATCATG

*trnS-trnG* (pairwise identity: 100%)


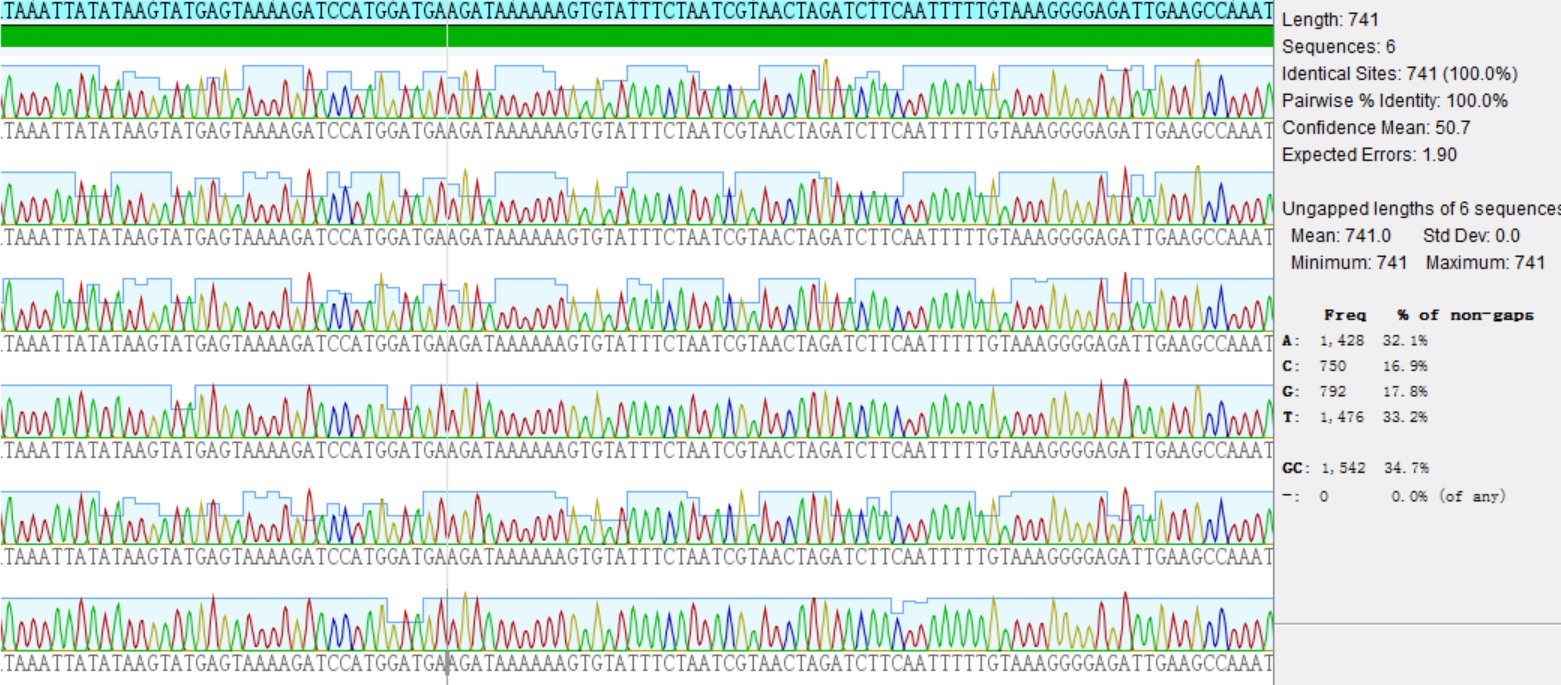


>NXQZS_1

TTCTTTCGGTTTGATACATTCATATTCCGATCAAAAACTTTATTTCTTACAAGGGTTTAATCCTTTCCTATCAATGCCACAATTGGGAATAATATAAATTTTCGTGATTTGAATCCAAAAACTCGGATTATGGAATCGCGAAGCATAATTTGTTTTCCAATTATAAATTATATAAGTATGAGTAAAAGATCCATGGATGAAGATAAAAAAGTGTATTTCTAATCGTAACTAGATCTTCAATTTTTGTAAAGGGGAGATTGAAGCCAAATAGCTATTAAACGATGACTTCGGTTTACTGGGGCCATCGGCATGTATATATTGTTTCAGCTCGGTAGAAATAAGATTCTTTTCCTAAGGATTCATGCCAGTAGAAATAGGGAACGAAGTAACTAGAAGGATTTTTATAATACCCCTCCTCTAGGAGGATCATCTAGAAAGCGAGTAGCGTTGGATACATTGAGACAGAAAAGCTGACATAGGTGTTATGGATCGAATTTTTCTTATTCCGATTTGGTTTGCTATGACTTCCTTCTTTTCCTTACATACCTTTCCATACTAATATGATACATCGTATCATACATCGTGAATTTTTTCGTTTAGGTAAATTTACACCCTAAATACCGGAATCTATCCATATTCCATAAAGGAGCCGAATGAAACAAAAGTTTCATGTTCGGTTTTGAATTAGAGACGTTCAAAATGATGAATCGACGTCGACTATAACCCCTAGCCTTCCAAGCTAACGATG

>GDSYD_1

TTCTTTCGGTTTGATACATTCATATTCCGATCAAAAACTTTATTTCTTACAAGGGTTTAATCCTTTCCTATCAATGCCACAATTGGGAATAATATAAATTTTCGTGATTTGAATCCAAAAACTCGGATTATGGAATCGCGAAGCATAATTTGTTTTCCAATTATAAATTATATAAGTATGAGTAAAAGATCCATGGATGAAGATAAAAAAGTGTATTTCTAATCGTAACTAGATCTTCAATTTTTGTAAAGGGGAGATTGAAGCCAAATAGCTATTAAACGATGACTTCGGTTTACTGGGGCCATCGGCATGTATATATTGTTTCAGCTCGGTAGAAATAAGATTCTTTTCCTAAGGATTCATGCCAGTAGAAATAGGGAACGAAGTAACTAGAAGGATTTTTATAATACCCCTCCTCTAGGAGGATCATCTAGAAAGCGAGTAGCGTTGGATACATTGAGACAGAAAAGCTGACATAGGTGTTATGGATCGAATTTTTCTTATTCCGATTTGGTTTGCTATGACTTCCTTCTTTTCCTTACATACCTTTCCATACTAATATGATACATCGTATCATACATCGTGAATTTTTTCGTTTAGGTAAATTTACACCCTAAATACCGGAATCTATCCATATTCCATAAAGGAGCCGAATGAAACAAAAGTTTCATGTTCGGTTTTGAATTAGAGACGTTCAAAATGATGAATCGACGTCGACTATAACCCCTAGCCTTCCAAGCTAACGATG

>NDXFS_1

TTCTTTCGGTTTGATACATTCATATTCCGATCAAAAACTTTATTTCTTACAAGGGTTTAATCCTTTCCTATCAATGCCACAATTGGGAATAATATAAATTTTCGTGATTTGAATCCAAAAACTCGGATTATGGAATCGCGAAGCATAATTTGTTTTCCAATTATAAATTATATAAGTATGAGTAAAAGATCCATGGATGAAGATAAAAAAGTGTATTTCTAATCGTAACTAGATCTTCAATTTTTGTAAAGGGGAGATTGAAGCCAAATAGCTATTAAACGATGACTTCGGTTTACTGGGGCCATCGGCATGTATATATTGTTTCAGCTCGGTAGAAATAAGATTCTTTTCCTAAGGATTCATGCCAGTAGAAATAGGGAACGAAGTAACTAGAAGGATTTTTATAATACCCCTCCTCTAGGAGGATCATCTAGAAAGCGAGTAGCGTTGGATACATTGAGACAGAAAAGCTGACATAGGTGTTATGGATCGAATTTTTCTTATTCCGATTTGGTTTGCTATGACTTCCTTCTTTTCCTTACATACCTTTCCATACTAATATGATACATCGTATCATACATCGTGAATTTTTTCGTTTAGGTAAATTTACACCCTAAATACCGGAATCTATCCATATTCCATAAAGGAGCCGAATGAAACAAAAGTTTCATGTTCGGTTTTGAATTAGAGACGTTCAAAATGATGAATCGACGTCGACTATAACCCCTAGCCTTCCAAGCTAACGATG

>TWYL_1

TTCTTTCGGTTTGATACATTCATATTCCGATCAAAAACTTTATTTCTTACAAGGGTTTAATCCTTTCCTATCAATGCCACAATTGGGAATAATATAAATTTTCGTGATTTGAATCCAAAAACTCGGATTATGGAATCGCGAAGCATAATTTGTTTTCCAATTATAAATTATATAAGTATGAGTAAAAGATCCATGGATGAAGATAAAAAAGTGTATTTCTAATCGTAACTAGATCTTCAATTTTTGTAAAGGGGAGATTGAAGCCAAATAGCTATTAAACGATGACTTCGGTTTACTGGGGCCATCGGCATGTATATATTGTTTCAGCTCGGTAGAAATAAGATTCTTTTCCTAAGGATTCATGCCAGTAGAAATAGGGAACGAAGTAACTAGAAGGATTTTTATAATACCCCTCCTCTAGGAGGATCATCTAGAAAGCGAGTAGCGTTGGATACATTGAGACAGAAAAGCTGACATAGGTGTTATGGATCGAATTTTTCTTATTCCGATTTGGTTTGCTATGACTTCCTTCTTTTCCTTACATACCTTTCCATACTAATATGATACATCGTATCATACATCGTGAATTTTTTCGTTTAGGTAAATTTACACCCTAAATACCGGAATCTATCCATATTCCATAAAGGAGCCGAATGAAACAAAAGTTTCATGTTCGGTTTTGAATTAGAGACGTTCAAAATGATGAATCGACGTCGACTATAACCCCTAGCCTTCCAAGCTAACGATG

>QYBSZ_1

TTCTTTCGGTTTGATACATTCATATTCCGATCAAAAACTTTATTTCTTACAAGGGTTTAATCCTTTCCTATCAATGCCACAATTGGGAATAATATAAATTTTCGTGATTTGAATCCAAAAACTCGGATTATGGAATCGCGAAGCATAATTTGTTTTCCAATTATAAATTATATAAGTATGAGTAAAAGATCCATGGATGAAGATAAAAAAGTGTATTTCTAATCGTAACTAGATCTTCAATTTTTGTAAAGGGGAGATTGAAGCCAAATAGCTATTAAACGATGACTTCGGTTTACTGGGGCCATCGGCATGTATATATTGTTTCAGCTCGGTAGAAATAAGATTCTTTTCCTAAGGATTCATGCCAGTAGAAATAGGGAACGAAGTAACTAGAAGGATTTTTATAATACCCCTCCTCTAGGAGGATCATCTAGAAAGCGAGTAGCGTTGGATACATTGAGACAGAAAAGCTGACATAGGTGTTATGGATCGAATTTTTCTTATTCCGATTTGGTTTGCTATGACTTCCTTCTTTTCCTTACATACCTTTCCATACTAATATGATACATCGTATCATACATCGTGAATTTTTTCGTTTAGGTAAATTTACACCCTAAATACCGGAATCTATCCATATTCCATAAAGGAGCCGAATGAAACAAAAGTTTCATGTTCGGTTTTGAATTAGAGACGTTCAAAATGATGAATCGACGTCGACTATAACCCCTAGCCTTCCAAGCTAACGATG

>YNTC_1

TTCTTTCGGTTTGATACATTCATATTCCGATCAAAAACTTTATTTCTTACAAGGGTTTAATCCTTTCCTATCAATGCCACAATTGGGAATAATATAAATTTTCGTGATTTGAATCCAAAAACTCGGATTATGGAATCGCGAAGCATAATTTGTTTTCCAATTATAAATTATATAAGTATGAGTAAAAGATCCATGGATGAAGATAAAAAAGTGTATTTCTAATCGTAACTAGATCTTCAATTTTTGTAAAGGGGAGATTGAAGCCAAATAGCTATTAAACGATGACTTCGGTTTACTGGGGCCATCGGCATGTATATATTGTTTCAGCTCGGTAGAAATAAGATTCTTTTCCTAAGGATTCATGCCAGTAGAAATAGGGAACGAAGTAACTAGAAGGATTTTTATAATACCCCTCCTCTAGGAGGATCATCTAGAAAGCGAGTAGCGTTGGATACATTGAGACAGAAAAGCTGACATAGGTGTTATGGATCGAATTTTTCTTATTCCGATTTGGTTTGCTATGACTTCCTTCTTTTCCTTACATACCTTTCCATACTAATATGATACATCGTATCATACATCGTGAATTTTTTCGTTTAGGTAAATTTACACCCTAAATACCGGAATCTATCCATATTCCATAAAGGAGCCGAATGAAACAAAAGTTTCATGTTCGGTTTTGAATTAGAGACGTTCAAAATGATGAATCGACGTCGACTATAACCCCTAGCCTTCCAAGCTAACGATG

*rpl14-rpl16* (pairwise identity: 100%)


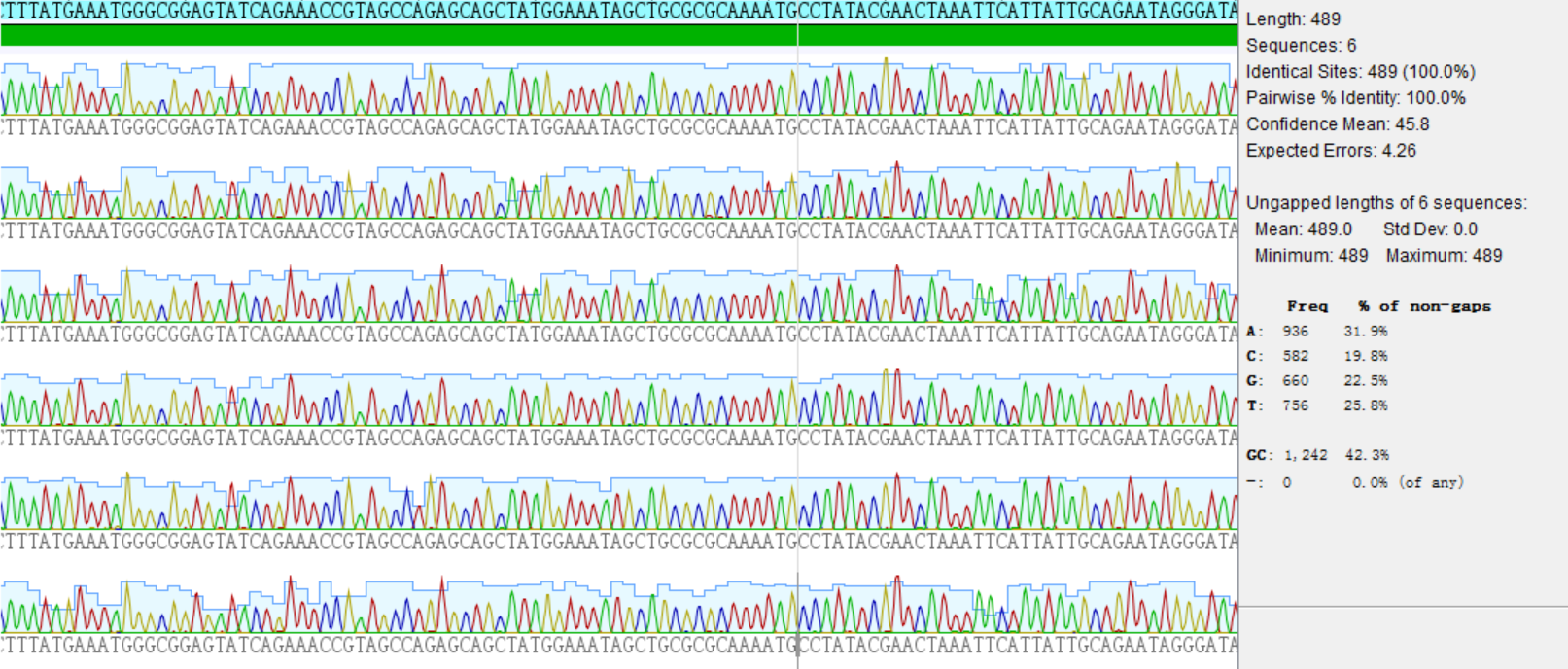


>NXQZS_1

CATTATCATCATACCGTATTATCATACCGTTGTCGCGTTTGAGCTCTTTACATGTACGTACAATTACAGCTCTTATTACTTCTGATCTTTCCAGGGGCATATTTGGCACTGCTTCTTTGATTACAGCAACAATAACGTCACCGATATGAGCATATCGGTAATTACTACCTCCTATGATTCGAATACACATCAGTTTTCGAGCTCCGCTGTTGTCCGCTACATTCAAATGAGTTTGAGGTTGAATCATCTTTTTTTTAAGTTCTTTCAATGCAAAGGCGAAGGAGAAAAAAAATAATTTTCTGTCCAGAAATAAGTAAACCATCGATTATAGATTATATTTTTCATCATTCATAAATATCCCCCTGGGCCGATATCCCTATTCTGCAATAATGAATTTAGTTCGTATAGGCATTTTGCGCGCAGCTATTTCCATAGCTGCTCTGGCTACGGTTTCTGATACTCCGCCCATTTCATAAAGTATTCGATCCGGTTTAACGACGGATACCCAATATTCAGGAGATCCCTTCCCCGAACCCATACGTGTTTCTGCGGGTCTTACTGTAACGGGTTTGTCGGGGAATATACGTACCCATATTTTTCCGCCACGACGGGCATATCGTGTCATGGCGCGTCGCCCTGCTTCTATTTGTCTAGATGTGATCCAAGCGGGTTCAAGTGCCTGAAGCGCATATCTCCCGAAACTTATATGATTGCCACGATAAGATACTCCTTTCATTCTTCCTCTATGTTGTTTACGAAACCTGGTTCTTTTGGGGTTATAGTTGATGGTAGTGCCTCAATCCCATCTCTACTACAGAACCGGACATGAGAGTTTCTTCTCATCCAGCTCCTCGCGAATGAAAGAATAAATAATAGTAAAATTATTTACTGAATGAAATTTCGCGGGCGAATATTTACTCTTTTATTTTTTTTTGCTTTATTCGTAGGGTCGTTCCATAACCTCTTCGAAGAAATAAGTTCGATCT

>GDSYD_1

CATTATCATCATACCGTATTATCATACCGTTGTCGCGTTTGAGCTCTTTACATGTACGTACAATTACAGCTCTTATTACTTCTGATCTTTCCAGGGGCATATTTGGCACTGCTTCTTTGATTACAGCAACAATAACGTCACCGATATGAGCATATCGGTAATTACTACCTCCTATGATTCGAATACACATCAGTTTTCGAGCTCCGCTGTTGTCCGCTACATTCAAATGAGTTTGAGGTTGAATCATCTTTTTTTTAAGTTCTTTCAATGCAAAGGCGAAGGAGAAAAAAAATAATTTTCTGTCCAGAAATAAGTAAACCATCGATTATAGATTATATTTTTCATCATTCATAAATATCCCCCTGGGCCGATATCCCTATTCTGCAATAATGAATTTAGTTCGTATAGGCATTTTGCGCGCAGCTATTTCCATAGCTGCTCTGGCTACGGTTTCTGATACTCCGCCCATTTCATAAAGTATTCGATCCGGTTTAACGACGGATACCCAATATTCAGGAGATCCCTTCCCCGAACCCATACGTGTTTCTGCGGGTCTTACTGTAACGGGTTTGTCGGGGAATATACGTACCCATATTTTTCCGCCACGACGGGCATATCGTGTCATGGCGCGTCGCCCTGCTTCTATTTGTCTAGATGTGATCCAAGCGGGTTCAAGTGCCTGAAGCGCATATCTCCCGAAACTTATATGATTGCCACGATAAGATACTCCTTTCATTCTTCCTCTATGTTGTTTACGAAACCTGGTTCTTTTGGGGTTATAGTTGATGGTAGTGCCTCAATCCCATCTCTACTACAGAACCGGACATGAGAGTTTCTTCTCATCCAGCTCCTCGCGAATGAAAGAATAAATAATAGTAAAATTATTTACTGAATGAAATTTCGCGGGCGAATATTTACTCTTTTATTTTTTTTTGCTTTATTCGTAGGGTCGTTCCATAACCTCTTCGAAGAAATAAGTTCGATCT

>NDXFS_1

CATTATCATCATACCGTATTATCATACCGTTGTCGCGTTTGAGCTCTTTACATGTACGTACAATTACAGCTCTTATTACTTCTGATCTTTCCAGGGGCATATTTGGCACTGCTTCTTTGATTACAGCAACAATAACGTCACCGATATGAGCATATCGGTAATTACTACCTCCTATGATTCGAATACACATCAGTTTTCGAGCTCCGCTGTTGTCCGCTACATTCAAATGAGTTTGAGGTTGAATCATCTTTTTTTTAAGTTCTTTCAATGCAAAGGCGAAGGAGAAAAAAAATAATTTTCTGTCCAGAAATAAGTAAACCATCGATTATAGATTATATTTTTCATCATTCATAAATATCCCCCTGGGCCGATATCCCTATTCTGCAATAATGAATTTAGTTCGTATAGGCATTTTGCGCGCAGCTATTTCCATAGCTGCTCTGGCTACGGTTTCTGATACTCCGCCCATTTCATAAAGTATTCGATCCGGTTTAACGACGGATACCCAATATTCAGGAGATCCCTTCCCCGAACCCATACGTGTTTCTGCGGGTCTTACTGTAACGGGTTTGTCGGGGAATATACGTACCCATATTTTTCCGCCACGACGGGCATATCGTGTCATGGCGCGTCGCCCTGCTTCTATTTGTCTAGATGTGATCCAAGCGGGTTCAAGTGCCTGAAGCGCATATCTCCCGAAACTTATATGATTGCCACGATAAGATACTCCTTTCATTCTTCCTCTATGTTGTTTACGAAACCTGGTTCTTTTGGGGTTATAGTTGATGGTAGTGCCTCAATCCCATCTCTACTACAGAACCGGACATGAGAGTTTCTTCTCATCCAGCTCCTCGCGAATGAAAGAATAAATAATAGTAAAATTATTTACTGAATGAAATTTCGCGGGCGAATATTTACTCTTTTATTTTTTTTTGCTTTATTCGTAGGGTCGTTCCATAACCTCTTCGAAGAAATAAGTTCGATCT

>TWYL_1

CATTATCATCATACCGTATTATCATACCGTTGTCGCGTTTGAGCTCTTTACATGTACGTACAATTACAGCTCTTATTACTTCTGATCTTTCCAGGGGCATATTTGGCACTGCTTCTTTGATTACAGCAACAATAACGTCACCGATATGAGCATATCGGTAATTACTACCTCCTATGATTCGAATACACATCAGTTTTCGAGCTCCGCTGTTGTCCGCTACATTCAAATGAGTTTGAGGTTGAATCATCTTTTTTTTAAGTTCTTTCAATGCAAAGGCGAAGGAGAAAAAAAATAATTTTCTGTCCAGAAATAAGTAAACCATCGATTATAGATTATATTTTTCATCATTCATAAATATCCCCCTGGGCCGATATCCCTATTCTGCAATAATGAATTTAGTTCGTATAGGCATTTTGCGCGCAGCTATTTCCATAGCTGCTCTGGCTACGGTTTCTGATACTCCGCCCATTTCATAAAGTATTCGATCCGGTTTAACGACGGATACCCAATATTCAGGAGATCCCTTCCCCGAACCCATACGTGTTTCTGCGGGTCTTACTGTAACGGGTTTGTCGGGGAATATACGTACCCATATTTTTCCGCCACGACGGGCATATCGTGTCATGGCGCGTCGCCCTGCTTCTATTTGTCTAGATGTGATCCAAGCGGGTTCAAGTGCCTGAAGCGCATATCTCCCGAAACTTATATGATTGCCACGATAAGATACTCCTTTCATTCTTCCTCTATGTTGTTTACGAAACCTGGTTCTTTTGGGGTTATAGTTGATGGTAGTGCCTCAATCCCATCTCTACTACAGAACCGGACATGAGAGTTTCTTCTCATCCAGCTCCTCGCGAATGAAAGAATAAATAATAGTAAAATTATTTACTGAATGAAATTTCGCGGGCGAATATTTACTCTTTTATTTTTTTTTGCTTTATTCGTAGGGTCGTTCCATAACCTCTTCGAAGAAATAAGTTCGATCT

>QYBSZ_1

CATTATCATCATACCGTATTATCATACCGTTGTCGCGTTTGAGCTCTTTACATGTACGTACAATTACAGCTCTTATTACTTCTGATCTTTCCAGGGGCATATTTGGCACTGCTTCTTTGATTACAGCAACAATAACGTCACCGATATGAGCATATCGGTAATTACTACCTCCTATGATTCGAATACACATCAGTTTTCGAGCTCCGCTGTTGTCCGCTACATTCAAATGAGTTTGAGGTTGAATCATCTTTTTTTTAAGTTCTTTCAATGCAAAGGCGAAGGAGAAAAAAAATAATTTTCTGTCCAGAAATAAGTAAACCATCGATTATAGATTATATTTTTCATCATTCATAAATATCCCCCTGGGCCGATATCCCTATTCTGCAATAATGAATTTAGTTCGTATAGGCATTTTGCGCGCAGCTATTTCCATAGCTGCTCTGGCTACGGTTTCTGATACTCCGCCCATTTCATAAAGTATTCGATCCGGTTTAACGACGGATACCCAATATTCAGGAGATCCCTTCCCCGAACCCATACGTGTTTCTGCGGGTCTTACTGTAACGGGTTTGTCGGGGAATATACGTACCCATATTTTTCCGCCACGACGGGCATATCGTGTCATGGCGCGTCGCCCTGCTTCTATTTGTCTAGATGTGATCCAAGCGGGTTCAAGTGCCTGAAGCGCATATCTCCCGAAACTTATATGATTGCCACGATAAGATACTCCTTTCATTCTTCCTCTATGTTGTTTACGAAACCTGGTTCTTTTGGGGTTATAGTTGATGGTAGTGCCTCAATCCCATCTCTACTACAGAACCGGACATGAGAGTTTCTTCTCATCCAGCTCCTCGCGAATGAAAGAATAAATAATAGTAAAATTATTTACTGAATGAAATTTCGCGGGCGAATATTTACTCTTTTATTTTTTTTTGCTTTATTCGTAGGGTCGTTCCATAACCTCTTCGAAGAAATAAGTTCGATCT

>YNTC_1

CATTATCATCATACCGTATTATCATACCGTTGTCGCGTTTGAGCTCTTTACATGTACGTACAATTACAGCTCTTATTACTTCTGATCTTTCCAGGGGCATATTTGGCACTGCTTCTTTGATTACAGCAACAATAACGTCACCGATATGAGCATATCGGTAATTACTACCTCCTATGATTCGAATACACATCAGTTTTCGAGCTCCGCTGTTGTCCGCTACATTCAAATGAGTTTGAGGTTGAATCATCTTTTTTTTAAGTTCTTTCAATGCAAAGGCGAAGGAGAAAAAAAATAATTTTCTGTCCAGAAATAAGTAAACCATCGATTATAGATTATATTTTTCATCATTCATAAATATCCCCCTGGGCCGATATCCCTATTCTGCAATAATGAATTTAGTTCGTATAGGCATTTTGCGCGCAGCTATTTCCATAGCTGCTCTGGCTACGGTTTCTGATACTCCGCCCATTTCATAAAGTATTCGATCCGGTTTAACGACGGATACCCAATATTCAGGAGATCCCTTCCCCGAACCCATACGTGTTTCTGCGGGTCTTACTGTAACGGGTTTGTCGGGGAATATACGTACCCATATTTTTCCGCCACGACGGGCATATCGTGTCATGGCGCGTCGCCCTGCTTCTATTTGTCTAGATGTGATCCAAGCGGGTTCAAGTGCCTGAAGCGCATATCTCCCGAAACTTATATGATTGCCACGATAAGATACTCCTTTCATTCTTCCTCTATGTTGTTTACGAAACCTGGTTCTTTTGGGGTTATAGTTGATGGTAGTGCCTCAATCCCATCTCTACTACAGAACCGGACATGAGAGTTTCTTCTCATCCAGCTCCTCGCGAATGAAAGAATAAATAATAGTAAAATTATTTACTGAATGAAATTTCGCGGGCGAATATTTACTCTTTTATTTTTTTTTGCTTTATTCGTAGGGTCGTTCCATAACCTCTTCGAAGAAATAAGTTCGATCT

*psbZ-trnG* (pairwise identity: 100%)


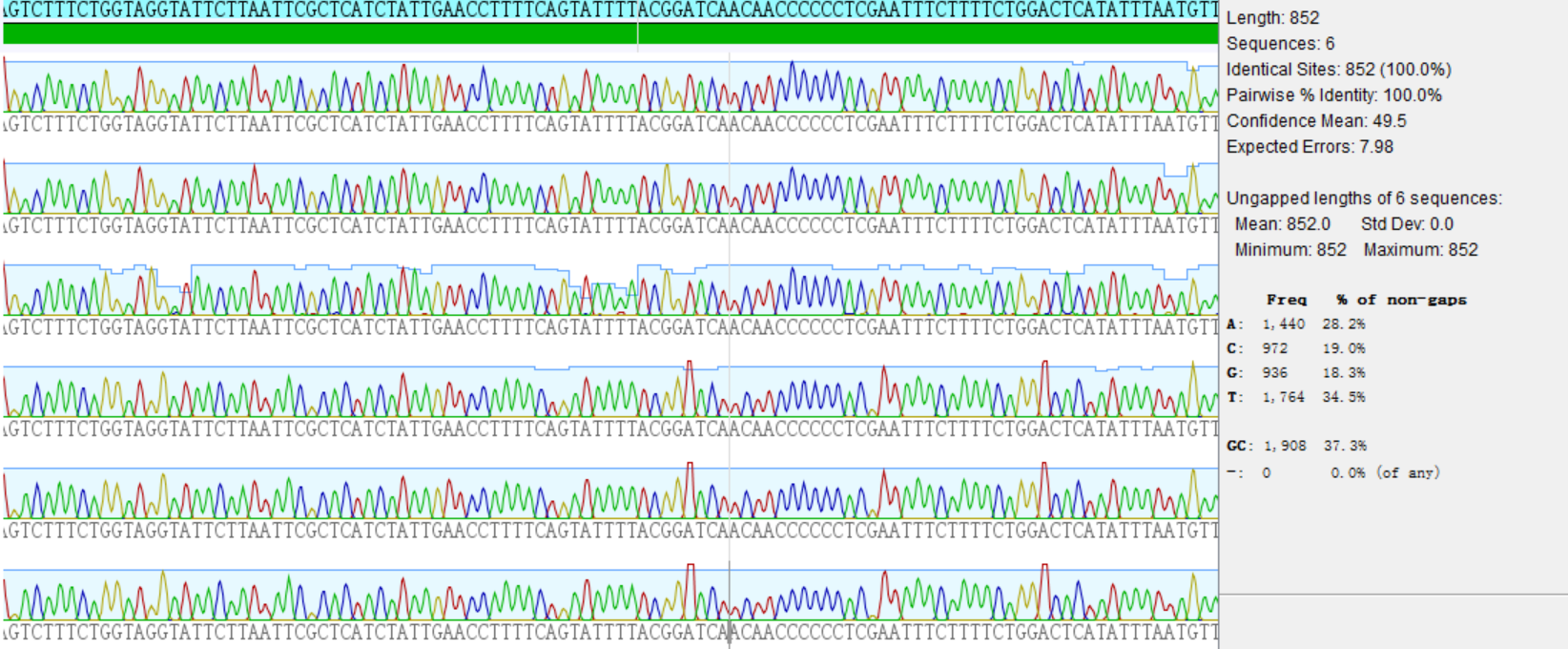


>NXQZS_1

TGTTTGTAAAAAAAATGTATATCTATTTCTTTTTAAGTGTGATTCTTTCACCTGAAAATTAATGCACTTTTTTTATTTGGGAGTCTGAGTAAAAGAAGAGGGAAAATACAACCACTACACTAGGTGTAGGTATATCAACTATACCGGATAATTCATCATATTACATTACTCCACCATGGGCGGATAGCGGGAATCGAACCCGCATCTTCTCCTTGGCAAAGAGAAATTTTACCATTCGACCATATCCGCATTTTATTCGTTCTTGATACACGCGTATGTGTCAACAAACACCTATGTGTTGATATATATCTATATATATTTATATCATATTTGTACAGAGTCAGATACTATCTTCAGGAAAATTCCAACAATTTTGCATTCAATTCATTCAAGAATTCACTTTTGACCCCCCCGAAGAAATTCTGGTTGTGGGACACATTAACATTAAATATGAGTCCAGAAAAGAAATTCGAGGGGGGTTGTTGATCCGTAAAATACTGAAAAGGTTCAATAGATGAGCGAATTAAGAATACCTACCAGAAAGACTAATCCAATCCATAATGACGTACCAGAAAATACAACATTTTTATTACTTGACCAACCCTCGGGAGAAGCAAATACAATGGGTACACTAATCAATAAGATTGATGAAGTAGCAATTAATGCAAAAACAGCCAATTGGAAAGCAATAGTCATGGTTGTAATCCTCCAAGCTACCGGCAAATGAATGAACTATACCATTTGATCCCTCTCTCGCACGAGATTGTAAGATGTAAGAAAATGCATCATAGAGGGATTTGGCCGTGAATCCATTGATCCTTTAGAACTCATTACTTATTCGGATCGTACATGGAGTCGAGGGTCCAGTTTTATTTACTTCAGAAACGACGGGCATGATGGATCATGCGCGATCCATATATACGGAACAG

>GDSYD_1

TGTTTGTAAAAAAAATGTATATCTATTTCTTTTTAAGTGTGATTCTTTCACCTGAAAATTAATGCACTTTTTTTATTTGGGAGTCTGAGTAAAAGAAGAGGGAAAATACAACCACTACACTAGGTGTAGGTATATCAACTATACCGGATAATTCATCATATTACATTACTCCACCATGGGCGGATAGCGGGAATCGAACCCGCATCTTCTCCTTGGCAAAGAGAAATTTTACCATTCGACCATATCCGCATTTTATTCGTTCTTGATACACGCGTATGTGTCAACAAACACCTATGTGTTGATATATATCTATATATATTTATATCATATTTGTACAGAGTCAGATACTATCTTCAGGAAAATTCCAACAATTTTGCATTCAATTCATTCAAGAATTCACTTTTGACCCCCCCGAAGAAATTCTGGTTGTGGGACACATTAACATTAAATATGAGTCCAGAAAAGAAATTCGAGGGGGGTTGTTGATCCGTAAAATACTGAAAAGGTTCAATAGATGAGCGAATTAAGAATACCTACCAGAAAGACTAATCCAATCCATAATGACGTACCAGAAAATACAACATTTTTATTACTTGACCAACCCTCGGGAGAAGCAAATACAATGGGTACACTAATCAATAAGATTGATGAAGTAGCAATTAATGCAAAAACAGCCAATTGGAAAGCAATAGTCATGGTTGTAATCCTCCAAGCTACCGGCAAATGAATGAACTATACCATTTGATCCCTCTCTCGCACGAGATTGTAAGATGTAAGAAAATGCATCATAGAGGGATTTGGCCGTGAATCCATTGATCCTTTAGAACTCATTACTTATTCGGATCGTACATGGAGTCGAGGGTCCAGTTTTATTTACTTCAGAAACGACGGGCATGATGGATCATGCGCGATCCATATATACGGAACAG

>NDXFS_1

TGTTTGTAAAAAAAATGTATATCTATTTCTTTTTAAGTGTGATTCTTTCACCTGAAAATTAATGCACTTTTTTTATTTGGGAGTCTGAGTAAAAGAAGAGGGAAAATACAACCACTACACTAGGTGTAGGTATATCAACTATACCGGATAATTCATCATATTACATTACTCCACCATGGGCGGATAGCGGGAATCGAACCCGCATCTTCTCCTTGGCAAAGAGAAATTTTACCATTCGACCATATCCGCATTTTATTCGTTCTTGATACACGCGTATGTGTCAACAAACACCTATGTGTTGATATATATCTATATATATTTATATCATATTTGTACAGAGTCAGATACTATCTTCAGGAAAATTCCAACAATTTTGCATTCAATTCATTCAAGAATTCACTTTTGACCCCCCCGAAGAAATTCTGGTTGTGGGACACATTAACATTAAATATGAGTCCAGAAAAGAAATTCGAGGGGGGTTGTTGATCCGTAAAATACTGAAAAGGTTCAATAGATGAGCGAATTAAGAATACCTACCAGAAAGACTAATCCAATCCATAATGACGTACCAGAAAATACAACATTTTTATTACTTGACCAACCCTCGGGAGAAGCAAATACAATGGGTACACTAATCAATAAGATTGATGAAGTAGCAATTAATGCAAAAACAGCCAATTGGAAAGCAATAGTCATGGTTGTAATCCTCCAAGCTACCGGCAAATGAATGAACTATACCATTTGATCCCTCTCTCGCACGAGATTGTAAGATGTAAGAAAATGCATCATAGAGGGATTTGGCCGTGAATCCATTGATCCTTTAGAACTCATTACTTATTCGGATCGTACATGGAGTCGAGGGTCCAGTTTTATTTACTTCAGAAACGACGGGCATGATGGATCATGCGCGATCCATATATACGGAACAG

>TWYL_1

TGTTTGTAAAAAAAATGTATATCTATTTCTTTTTAAGTGTGATTCTTTCACCTGAAAATTAATGCACTTTTTTTATTTGGGAGTCTGAGTAAAAGAAGAGGGAAAATACAACCACTACACTAGGTGTAGGTATATCAACTATACCGGATAATTCATCATATTACATTACTCCACCATGGGCGGATAGCGGGAATCGAACCCGCATCTTCTCCTTGGCAAAGAGAAATTTTACCATTCGACCATATCCGCATTTTATTCGTTCTTGATACACGCGTATGTGTCAACAAACACCTATGTGTTGATATATATCTATATATATTTATATCATATTTGTACAGAGTCAGATACTATCTTCAGGAAAATTCCAACAATTTTGCATTCAATTCATTCAAGAATTCACTTTTGACCCCCCCGAAGAAATTCTGGTTGTGGGACACATTAACATTAAATATGAGTCCAGAAAAGAAATTCGAGGGGGGTTGTTGATCCGTAAAATACTGAAAAGGTTCAATAGATGAGCGAATTAAGAATACCTACCAGAAAGACTAATCCAATCCATAATGACGTACCAGAAAATACAACATTTTTATTACTTGACCAACCCTCGGGAGAAGCAAATACAATGGGTACACTAATCAATAAGATTGATGAAGTAGCAATTAATGCAAAAACAGCCAATTGGAAAGCAATAGTCATGGTTGTAATCCTCCAAGCTACCGGCAAATGAATGAACTATACCATTTGATCCCTCTCTCGCACGAGATTGTAAGATGTAAGAAAATGCATCATAGAGGGATTTGGCCGTGAATCCATTGATCCTTTAGAACTCATTACTTATTCGGATCGTACATGGAGTCGAGGGTCCAGTTTTATTTACTTCAGAAACGACGGGCATGATGGATCATGCGCGATCCATATATACGGAACAG

>QYBSZ_1

TGTTTGTAAAAAAAATGTATATCTATTTCTTTTTAAGTGTGATTCTTTCACCTGAAAATTAATGCACTTTTTTTATTTGGGAGTCTGAGTAAAAGAAGAGGGAAAATACAACCACTACACTAGGTGTAGGTATATCAACTATACCGGATAATTCATCATATTACATTACTCCACCATGGGCGGATAGCGGGAATCGAACCCGCATCTTCTCCTTGGCAAAGAGAAATTTTACCATTCGACCATATCCGCATTTTATTCGTTCTTGATACACGCGTATGTGTCAACAAACACCTATGTGTTGATATATATCTATATATATTTATATCATATTTGTACAGAGTCAGATACTATCTTCAGGAAAATTCCAACAATTTTGCATTCAATTCATTCAAGAATTCACTTTTGACCCCCCCGAAGAAATTCTGGTTGTGGGACACATTAACATTAAATATGAGTCCAGAAAAGAAATTCGAGGGGGGTTGTTGATCCGTAAAATACTGAAAAGGTTCAATAGATGAGCGAATTAAGAATACCTACCAGAAAGACTAATCCAATCCATAATGACGTACCAGAAAATACAACATTTTTATTACTTGACCAACCCTCGGGAGAAGCAAATACAATGGGTACACTAATCAATAAGATTGATGAAGTAGCAATTAATGCAAAAACAGCCAATTGGAAAGCAATAGTCATGGTTGTAATCCTCCAAGCTACCGGCAAATGAATGAACTATACCATTTGATCCCTCTCTCGCACGAGATTGTAAGATGTAAGAAAATGCATCATAGAGGGATTTGGCCGTGAATCCATTGATCCTTTAGAACTCATTACTTATTCGGATCGTACATGGAGTCGAGGGTCCAGTTTTATTTACTTCAGAAACGACGGGCATGATGGATCATGCGCGATCCATATATACGGAACAG

>YNTC_1

TGTTTGTAAAAAAAATGTATATCTATTTCTTTTTAAGTGTGATTCTTTCACCTGAAAATTAATGCACTTTTTTTATTTGGGAGTCTGAGTAAAAGAAGAGGGAAAATACAACCACTACACTAGGTGTAGGTATATCAACTATACCGGATAATTCATCATATTACATTACTCCACCATGGGCGGATAGCGGGAATCGAACCCGCATCTTCTCCTTGGCAAAGAGAAATTTTACCATTCGACCATATCCGCATTTTATTCGTTCTTGATACACGCGTATGTGTCAACAAACACCTATGTGTTGATATATATCTATATATATTTATATCATATTTGTACAGAGTCAGATACTATCTTCAGGAAAATTCCAACAATTTTGCATTCAATTCATTCAAGAATTCACTTTTGACCCCCCCGAAGAAATTCTGGTTGTGGGACACATTAACATTAAATATGAGTCCAGAAAAGAAATTCGAGGGGGGTTGTTGATCCGTAAAATACTGAAAAGGTTCAATAGATGAGCGAATTAAGAATACCTACCAGAAAGACTAATCCAATCCATAATGACGTACCAGAAAATACAACATTTTTATTACTTGACCAACCCTCGGGAGAAGCAAATACAATGGGTACACTAATCAATAAGATTGATGAAGTAGCAATTAATGCAAAAACAGCCAATTGGAAAGCAATAGTCATGGTTGTAATCCTCCAAGCTACCGGCAAATGAATGAACTATACCATTTGATCCCTCTCTCGCACGAGATTGTAAGATGTAAGAAAATGCATCATAGAGGGATTTGGCCGTGAATCCATTGATCCTTTAGAACTCATTACTTATTCGGATCGTACATGGAGTCGAGGGTCCAGTTTTATTTACTTCAGAAACGACGGGCATGATGGATCATGCGCGATCCATATATACGGAACAG

*trnC-rpoB* (pairwise identity: 100%)


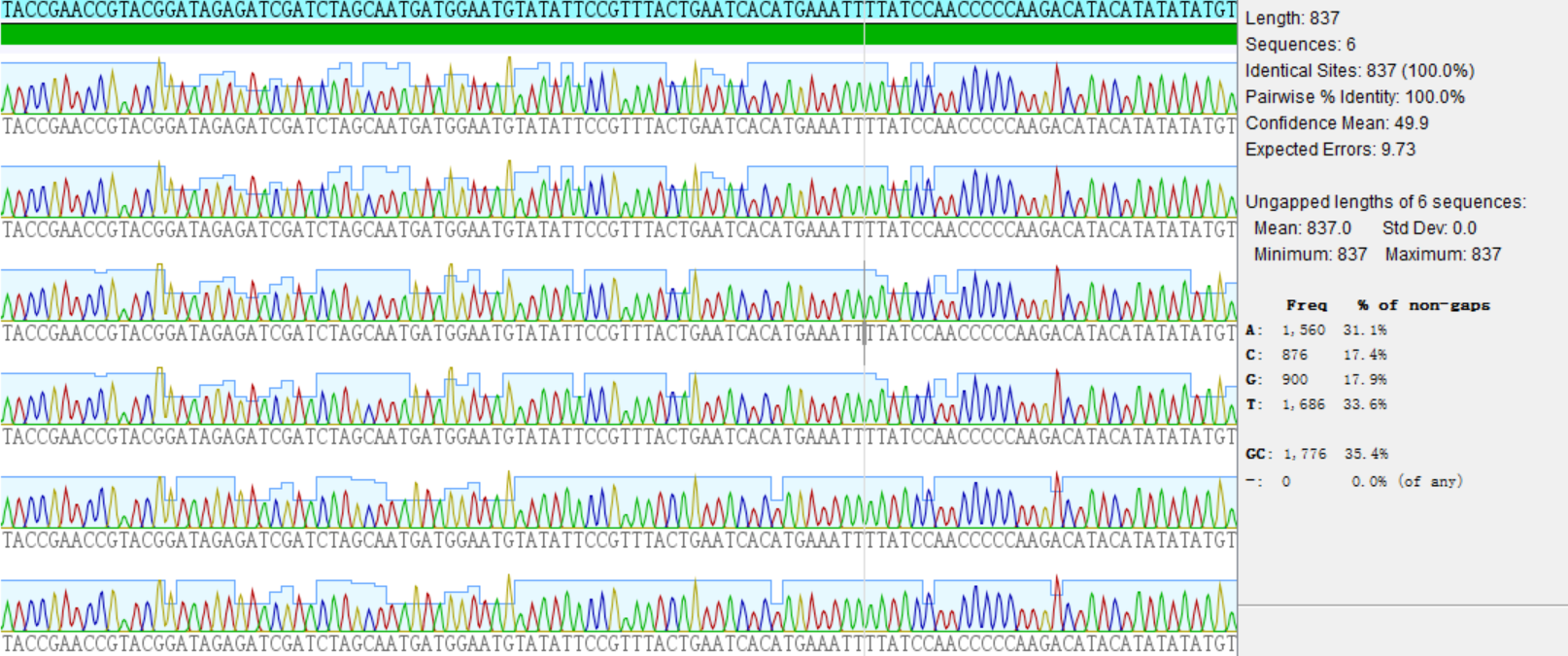


>NXQZS_1

TTCGCTGATATAACTCGCCCGATTTTTGACCGGCGGACGAAAAGAGCTGTCCATACTTTATTTATCTTCAAAATAAGCTTCAAAATACGCAGGTTCTATGAAAGAAATAGAACTCTTTGCTCCGAGGATCCCATCCAGGGGGCGTGACTTATTGTAAAGACTATAACTATCAATTTTTACTAATTACTTTACCCTACCAGTAATCCACTAATATCCACTGGAGTGTCTCCTGATTCCGTCGTGAATTAGATCTTGCTTGGATTCGCCTTGGGGGAATCCAATTAGGTATTGTGGAGGGGGCTGAAGAAACTTTGTCTTTGTGTATATACCATACTCGCGATAGGATTTCAGTGCTCAGCCAGTCATTCAATAGTGAATGGTTGACTGGTCCCTATAACTATAAAGTAAAAAAGAACTATGACACCCGTAATAGGGTGTCTAACGATTGTTTGACAGAAACAGAGATAGTAAGGTTTAGCTTAGATCAAGGGGAGATACAGTTATCCATCTTGATGGTATATATGTATATATAGATTTTCCCTATGAAAAGCAACAAAACAAAGAATAGGTCTTACTATTAGGACATGTTCTATTCCCTTAGTAGCATCCCCTTGCTACTTCCAATGGGTA

>GDSYD_1

TTCGCTGATATAACTCGCCCGATTTTTGACCGGCGGACGAAAAGAGCTGTCCATACTTTATTTATCTTCAAAATAAGCTTCAAAATACGCAGGTTCTATGAAAGAAATAGAACTCTTTGCTCCGAGGATCCCATCCAGGGGGCGTGACTTATTGTAAAGACTATAACTATCAATTTTTACTAATTACTTTACCCTACCAGTAATCCACTAATATCCACTGGAGTGTCTCCTGATTCCGTCGTGAATTAGATCTTGCTTGGATTCGCCTTGGGGGAATCCAATTAGGTATTGTGGAGGGGGCTGAAGAAACTTTGTCTTTGTGTATATACCATACTCGCGATAGGATTTCAGTGCTCAGCCAGTCATTCAATAGTGAATGGTTGACTGGTCCCTATAACTATAAAGTAAAAAAGAACTATGACACCCGTAATAGGGTGTCTAACGATTGTTTGACAGAAACAGAGATAGTAAGGTTTAGCTTAGATCAAGGGGAGATACAGTTATCCATCTTGATGGTATATATGTATATATAGATTTTCCCTATGAAAAGCAACAAAACAAAGAATAGGTCTTACTATTAGGACATGTTCTATTCCCTTAGTAGCATCCCCTTGCTACTTCCAATGGGTA

>NDXFS_1

TTCGCTGATATAACTCGCCCGATTTTTGACCGGCGGACGAAAAGAGCTGTCCATACTTTATTTATCTTCAAAATAAGCTTCAAAATACGCAGGTTCTATGAAAGAAATAGAACTCTTTGCTCCGAGGATCCCATCCAGGGGGCGTGACTTATTGTAAAGACTATAACTATCAATTTTTACTAATTACTTTACCCTACCAGTAATCCACTAATATCCACTGGAGTGTCTCCTGATTCCGTCGTGAATTAGATCTTGCTTGGATTCGCCTTGGGGGAATCCAATTAGGTATTGTGGAGGGGGCTGAAGAAACTTTGTCTTTGTGTATATACCATACTCGCGATAGGATTTCAGTGCTCAGCCAGTCATTCAATAGTGAATGGTTGACTGGTCCCTATAACTATAAAGTAAAAAAGAACTATGACACCCGTAATAGGGTGTCTAACGATTGTTTGACAGAAACAGAGATAGTAAGGTTTAGCTTAGATCAAGGGGAGATACAGTTATCCATCTTGATGGTATATATGTATATATAGATTTTCCCTATGAAAAGCAACAAAACAAAGAATAGGTCTTACTATTAGGACATGTTCTATTCCCTTAGTAGCATCCCCTTGCTACTTCCAATGGGTA

>TWYL_1

TTCGCTGATATAACTCGCCCGATTTTTGACCGGCGGACGAAAAGAGCTGTCCATACTTTATTTATCTTCAAAATAAGCTTCAAAATACGCAGGTTCTATGAAAGAAATAGAACTCTTTGCTCCGAGGATCCCATCCAGGGGGCGTGACTTATTGTAAAGACTATAACTATCAATTTTTACTAATTACTTTACCCTACCAGTAATCCACTAATATCCACTGGAGTGTCTCCTGATTCCGTCGTGAATTAGATCTTGCTTGGATTCGCCTTGGGGGAATCCAATTAGGTATTGTGGAGGGGGCTGAAGAAACTTTGTCTTTGTGTATATACCATACTCGCGATAGGATTTCAGTGCTCAGCCAGTCATTCAATAGTGAATGGTTGACTGGTCCCTATAACTATAAAGTAAAAAAGAACTATGACACCCGTAATAGGGTGTCTAACGATTGTTTGACAGAAACAGAGATAGTAAGGTTTAGCTTAGATCAAGGGGAGATACAGTTATCCATCTTGATGGTATATATGTATATATAGATTTTCCCTATGAAAAGCAACAAAACAAAGAATAGGTCTTACTATTAGGACATGTTCTATTCCCTTAGTAGCATCCCCTTGCTACTTCCAATGGGTA

>QYBSZ_1

TTCGCTGATATAACTCGCCCGATTTTTGACCGGCGGACGAAAAGAGCTGTCCATACTTTATTTATCTTCAAAATAAGCTTCAAAATACGCAGGTTCTATGAAAGAAATAGAACTCTTTGCTCCGAGGATCCCATCCAGGGGGCGTGACTTATTGTAAAGACTATAACTATCAATTTTTACTAATTACTTTACCCTACCAGTAATCCACTAATATCCACTGGAGTGTCTCCTGATTCCGTCGTGAATTAGATCTTGCTTGGATTCGCCTTGGGGGAATCCAATTAGGTATTGTGGAGGGGGCTGAAGAAACTTTGTCTTTGTGTATATACCATACTCGCGATAGGATTTCAGTGCTCAGCCAGTCATTCAATAGTGAATGGTTGACTGGTCCCTATAACTATAAAGTAAAAAAGAACTATGACACCCGTAATAGGGTGTCTAACGATTGTTTGACAGAAACAGAGATAGTAAGGTTTAGCTTAGATCAAGGGGAGATACAGTTATCCATCTTGATGGTATATATGTATATATAGATTTTCCCTATGAAAAGCAACAAAACAAAGAATAGGTCTTACTATTAGGACATGTTCTATTCCCTTAGTAGCATCCCCTTGCTACTTCCAATGGGTA

>YNTC_1

TTCGCTGATATAACTCGCCCGATTTTTGACCGGCGGACGAAAAGAGCTGTCCATACTTTATTTATCTTCAAAATAAGCTTCAAAATACGCAGGTTCTATGAAAGAAATAGAACTCTTTGCTCCGAGGATCCCATCCAGGGGGCGTGACTTATTGTAAAGACTATAACTATCAATTTTTACTAATTACTTTACCCTACCAGTAATCCACTAATATCCACTGGAGTGTCTCCTGATTCCGTCGTGAATTAGATCTTGCTTGGATTCGCCTTGGGGGAATCCAATTAGGTATTGTGGAGGGGGCTGAAGAAACTTTGTCTTTGTGTATATACCATACTCGCGATAGGATTTCAGTGCTCAGCCAGTCATTCAATAGTGAATGGTTGACTGGTCCCTATAACTATAAAGTAAAAAAGAACTATGACACCCGTAATAGGGTGTCTAACGATTGTTTGACAGAAACAGAGATAGTAAGGTTTAGCTTAGATCAAGGGGAGATACAGTTATCCATCTTGATGGTATATATGTATATATAGATTTTCCCTATGAAAAGCAACAAAACAAAGAATAGGTCTTACTATTAGGACATGTTCTATTCCCTTAGTAGCATCCCCTTGCTACTTCCAATGGGTA

*psbZ-trnfM* (pairwise identity: 100%)


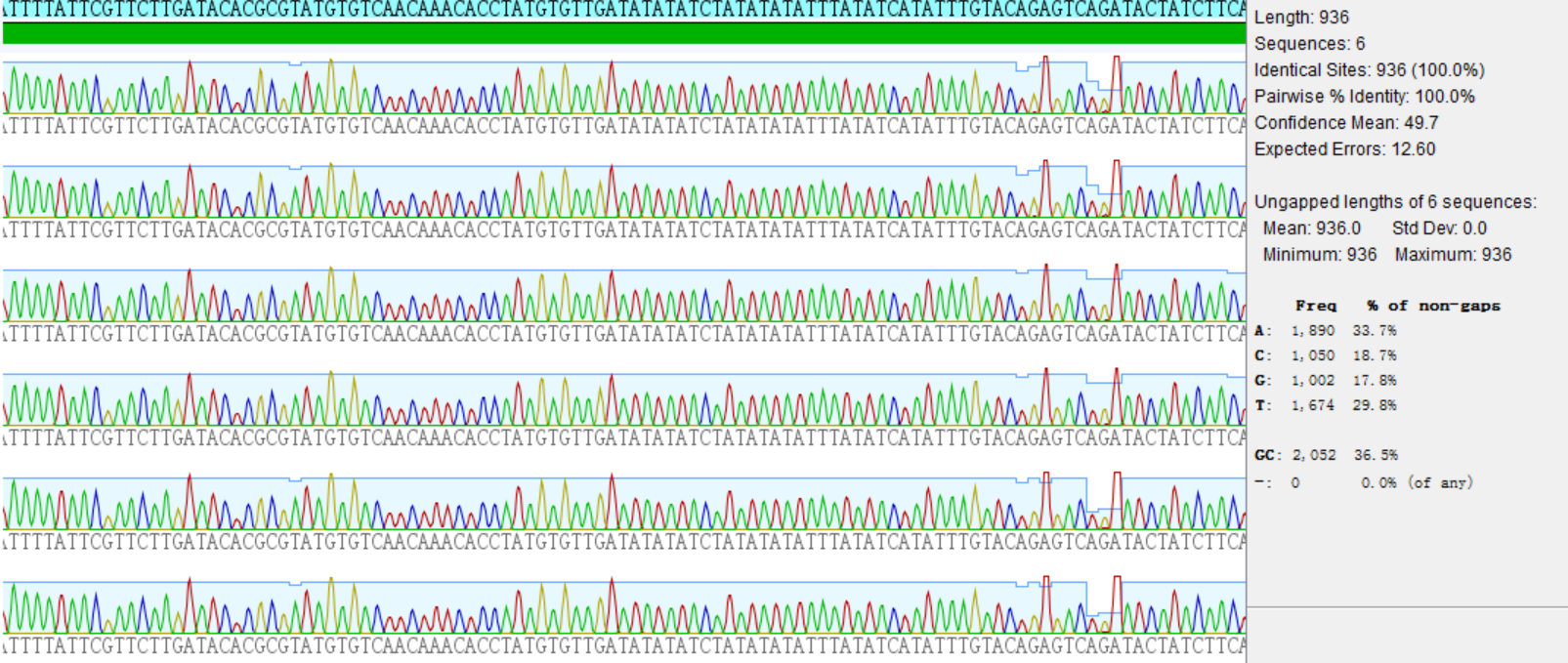


>NXQZS_1

TAATGAGTTCTAAAGGATCAATGGATTCACGGCCAAATCCCTCTATGATGCATTTTCTTACATCTTACAATCTCGTGCGAGAGAGGGATCAAATGGTATAGTTCATTCATTTGCCGGTAGCTTGGAGGATTACAACCATGACTATTGCTTTCCAATTGGCTGTTTTTGCATTAATTGCTACTTCATCAATCTTATTGATTAGTGTACCCATTGTATTTGCTTCTCCCGAGGGTTGGTCAAGTAATAAAAATGTTGTATTTTCTGGTACGTCATTATGGATTGGATTAGTCTTTCTGGTAGGTATTCTTAATTCGCTCATCTATTGAACCTTTTCAGTATTTTACGGATCAACAACCCCCCTCGAATTTCTTTTCTGGACTCATATTTAATGTTAATGTGTCCCACAACCAGAATTTCTTCGGGGGGGTCAAAAGTGAATTCTTGAATGAATTGAATGCAAAATTGTTGGAATTTTCCTGAAGATAGTATCTGACTCTGTACAAATATGATATAAATATATATAGATATATATCAACACATAGGTGTTTGTTGACACATACGCGTGTATCAAGAACGAATAAAATGCGGATATGGTCGAATGGTAAAATTTCTCTT

>GDSYD_1

TAATGAGTTCTAAAGGATCAATGGATTCACGGCCAAATCCCTCTATGATGCATTTTCTTACATCTTACAATCTCGTGCGAGAGAGGGATCAAATGGTATAGTTCATTCATTTGCCGGTAGCTTGGAGGATTACAACCATGACTATTGCTTTCCAATTGGCTGTTTTTGCATTAATTGCTACTTCATCAATCTTATTGATTAGTGTACCCATTGTATTTGCTTCTCCCGAGGGTTGGTCAAGTAATAAAAATGTTGTATTTTCTGGTACGTCATTATGGATTGGATTAGTCTTTCTGGTAGGTATTCTTAATTCGCTCATCTATTGAACCTTTTCAGTATTTTACGGATCAACAACCCCCCTCGAATTTCTTTTCTGGACTCATATTTAATGTTAATGTGTCCCACAACCAGAATTTCTTCGGGGGGGTCAAAAGTGAATTCTTGAATGAATTGAATGCAAAATTGTTGGAATTTTCCTGAAGATAGTATCTGACTCTGTACAAATATGATATAAATATATATAGATATATATCAACACATAGGTGTTTGTTGACACATACGCGTGTATCAAGAACGAATAAAATGCGGATATGGTCGAATGGTAAAATTTCTCTT

>NDXFS_1

TAATGAGTTCTAAAGGATCAATGGATTCACGGCCAAATCCCTCTATGATGCATTTTCTTACATCTTACAATCTCGTGCGAGAGAGGGATCAAATGGTATAGTTCATTCATTTGCCGGTAGCTTGGAGGATTACAACCATGACTATTGCTTTCCAATTGGCTGTTTTTGCATTAATTGCTACTTCATCAATCTTATTGATTAGTGTACCCATTGTATTTGCTTCTCCCGAGGGTTGGTCAAGTAATAAAAATGTTGTATTTTCTGGTACGTCATTATGGATTGGATTAGTCTTTCTGGTAGGTATTCTTAATTCGCTCATCTATTGAACCTTTTCAGTATTTTACGGATCAACAACCCCCCTCGAATTTCTTTTCTGGACTCATATTTAATGTTAATGTGTCCCACAACCAGAATTTCTTCGGGGGGGTCAAAAGTGAATTCTTGAATGAATTGAATGCAAAATTGTTGGAATTTTCCTGAAGATAGTATCTGACTCTGTACAAATATGATATAAATATATATAGATATATATCAACACATAGGTGTTTGTTGACACATACGCGTGTATCAAGAACGAATAAAATGCGGATATGGTCGAATGGTAAAATTTCTCTT

>TWYL_1

TAATGAGTTCTAAAGGATCAATGGATTCACGGCCAAATCCCTCTATGATGCATTTTCTTACATCTTACAATCTCGTGCGAGAGAGGGATCAAATGGTATAGTTCATTCATTTGCCGGTAGCTTGGAGGATTACAACCATGACTATTGCTTTCCAATTGGCTGTTTTTGCATTAATTGCTACTTCATCAATCTTATTGATTAGTGTACCCATTGTATTTGCTTCTCCCGAGGGTTGGTCAAGTAATAAAAATGTTGTATTTTCTGGTACGTCATTATGGATTGGATTAGTCTTTCTGGTAGGTATTCTTAATTCGCTCATCTATTGAACCTTTTCAGTATTTTACGGATCAACAACCCCCCTCGAATTTCTTTTCTGGACTCATATTTAATGTTAATGTGTCCCACAACCAGAATTTCTTCGGGGGGGTCAAAAGTGAATTCTTGAATGAATTGAATGCAAAATTGTTGGAATTTTCCTGAAGATAGTATCTGACTCTGTACAAATATGATATAAATATATATAGATATATATCAACACATAGGTGTTTGTTGACACATACGCGTGTATCAAGAACGAATAAAATGCGGATATGGTCGAATGGTAAAATTTCTCTT

>QYBSZ_1

TAATGAGTTCTAAAGGATCAATGGATTCACGGCCAAATCCCTCTATGATGCATTTTCTTACATCTTACAATCTCGTGCGAGAGAGGGATCAAATGGTATAGTTCATTCATTTGCCGGTAGCTTGGAGGATTACAACCATGACTATTGCTTTCCAATTGGCTGTTTTTGCATTAATTGCTACTTCATCAATCTTATTGATTAGTGTACCCATTGTATTTGCTTCTCCCGAGGGTTGGTCAAGTAATAAAAATGTTGTATTTTCTGGTACGTCATTATGGATTGGATTAGTCTTTCTGGTAGGTATTCTTAATTCGCTCATCTATTGAACCTTTTCAGTATTTTACGGATCAACAACCCCCCTCGAATTTCTTTTCTGGACTCATATTTAATGTTAATGTGTCCCACAACCAGAATTTCTTCGGGGGGGTCAAAAGTGAATTCTTGAATGAATTGAATGCAAAATTGTTGGAATTTTCCTGAAGATAGTATCTGACTCTGTACAAATATGATATAAATATATATAGATATATATCAACACATAGGTGTTTGTTGACACATACGCGTGTATCAAGAACGAATAAAATGCGGATATGGTCGAATGGTAAAATTTCTCTT

>YNTC_1

TAATGAGTTCTAAAGGATCAATGGATTCACGGCCAAATCCCTCTATGATGCATTTTCTTACATCTTACAATCTCGTGCGAGAGAGGGATCAAATGGTATAGTTCATTCATTTGCCGGTAGCTTGGAGGATTACAACCATGACTATTGCTTTCCAATTGGCTGTTTTTGCATTAATTGCTACTTCATCAATCTTATTGATTAGTGTACCCATTGTATTTGCTTCTCCCGAGGGTTGGTCAAGTAATAAAAATGTTGTATTTTCTGGTACGTCATTATGGATTGGATTAGTCTTTCTGGTAGGTATTCTTAATTCGCTCATCTATTGAACCTTTTCAGTATTTTACGGATCAACAACCCCCCTCGAATTTCTTTTCTGGACTCATATTTAATGTTAATGTGTCCCACAACCAGAATTTCTTCGGGGGGGTCAAAAGTGAATTCTTGAATGAATTGAATGCAAAATTGTTGGAATTTTCCTGAAGATAGTATCTGACTCTGTACAAATATGATATAAATATATATAGATATATATCAACACATAGGTGTTTGTTGACACATACGCGTGTATCAAGAACGAATAAAATGCGGATATGGTCGAATGGTAAAATTTCTCTT

*matK-psbA* (pairwise identity: 100%)


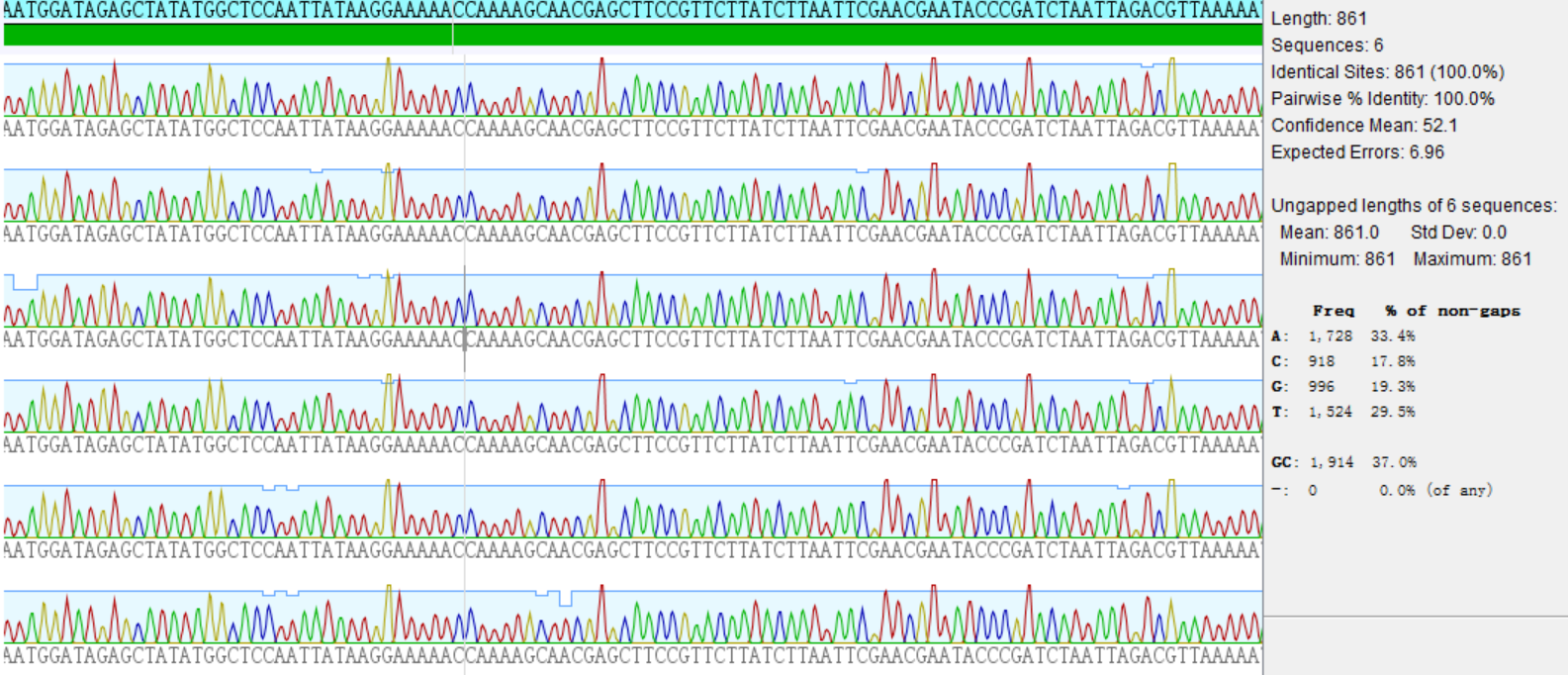


>NXQZS_1

GAGCGACCCCATAGGCTTGTGCTTTCGCGTCTCTCTAAAATTGCAGTCATGTTAAAATCTTGGTTTATTCAATCATCAGGAGCTCCCAAGCACACAGATTATCTATAAATGGATAATAGAAGGCTTGTTATTCAACAGTATAACATGCCTTATATGCCCGTGTCAACCAAAGCAATATCAACAGACTTCTTTCTATTCTTTTCTTTCCATACGGAAAGATCCATCCGAGCGATCTGTGAATGAAGTGAGTAGAATACATATTCTAGATGGTTTAGTTTAATTAAATTTGATAAAATTACGTATTTAATGTATGGTAGTGGGTTGCCCGGGACTCGAACCCGGAACTAGTCGGATGGAGTAGATAATCTCTTTGTTAATAGGAAAAAAAGAATCTCTCCCCAAACCGTGCTTGCATTTTTCATTGCACACGGCTTTCTCTGTGTATACATCTAAAACGAAGTTCCCTAGAAAATAGAACTCTAATAAGAAACTTGAATACTCAGTCGATTCAACCACTAGTACATGAAATGAGCATTTCATTAATAGATGAAATTTGGAATTTTTATATTTACGAATTAAATCTCGTCATTAGCATTAGTTGTTATGATTCCCATTTACTCCACACCCAATTATGAATTGGGTACCAGACCATTGATACGGATAATATCCAAATACCAAATCCGTTCGCTATGTAAACGGGTTCTTCGGAAGATGAAAGAAACAATTTCTTGTTCCTCTGTAAGGAACTCTTCCAATAGTTTCGAACCTAATCTCTTACAGATTGCGCGTACCGTACTTTT

>GDSYD_1

GAGCGACCCCATAGGCTTGTGCTTTCGCGTCTCTCTAAAATTGCAGTCATGTTAAAATCTTGGTTTATTCAATCATCAGGAGCTCCCAAGCACACAGATTATCTATAAATGGATAATAGAAGGCTTGTTATTCAACAGTATAACATGCCTTATATGCCCGTGTCAACCAAAGCAATATCAACAGACTTCTTTCTATTCTTTTCTTTCCATACGGAAAGATCCATCCGAGCGATCTGTGAATGAAGTGAGTAGAATACATATTCTAGATGGTTTAGTTTAATTAAATTTGATAAAATTACGTATTTAATGTATGGTAGTGGGTTGCCCGGGACTCGAACCCGGAACTAGTCGGATGGAGTAGATAATCTCTTTGTTAATAGGAAAAAAAGAATCTCTCCCCAAACCGTGCTTGCATTTTTCATTGCACACGGCTTTCTCTGTGTATACATCTAAAACGAAGTTCCCTAGAAAATAGAACTCTAATAAGAAACTTGAATACTCAGTCGATTCAACCACTAGTACATGAAATGAGCATTTCATTAATAGATGAAATTTGGAATTTTTATATTTACGAATTAAATCTCGTCATTAGCATTAGTTGTTATGATTCCCATTTACTCCACACCCAATTATGAATTGGGTACCAGACCATTGATACGGATAATATCCAAATACCAAATCCGTTCGCTATGTAAACGGGTTCTTCGGAAGATGAAAGAAACAATTTCTTGTTCCTCTGTAAGGAACTCTTCCAATAGTTTCGAACCTAATCTCTTACAGATTGCGCGTACCGTACTTTT

>NDXFS_1

GAGCGACCCCATAGGCTTGTGCTTTCGCGTCTCTCTAAAATTGCAGTCATGTTAAAATCTTGGTTTATTCAATCATCAGGAGCTCCCAAGCACACAGATTATCTATAAATGGATAATAGAAGGCTTGTTATTCAACAGTATAACATGCCTTATATGCCCGTGTCAACCAAAGCAATATCAACAGACTTCTTTCTATTCTTTTCTTTCCATACGGAAAGATCCATCCGAGCGATCTGTGAATGAAGTGAGTAGAATACATATTCTAGATGGTTTAGTTTAATTAAATTTGATAAAATTACGTATTTAATGTATGGTAGTGGGTTGCCCGGGACTCGAACCCGGAACTAGTCGGATGGAGTAGATAATCTCTTTGTTAATAGGAAAAAAAGAATCTCTCCCCAAACCGTGCTTGCATTTTTCATTGCACACGGCTTTCTCTGTGTATACATCTAAAACGAAGTTCCCTAGAAAATAGAACTCTAATAAGAAACTTGAATACTCAGTCGATTCAACCACTAGTACATGAAATGAGCATTTCATTAATAGATGAAATTTGGAATTTTTATATTTACGAATTAAATCTCGTCATTAGCATTAGTTGTTATGATTCCCATTTACTCCACACCCAATTATGAATTGGGTACCAGACCATTGATACGGATAATATCCAAATACCAAATCCGTTCGCTATGTAAACGGGTTCTTCGGAAGATGAAAGAAACAATTTCTTGTTCCTCTGTAAGGAACTCTTCCAATAGTTTCGAACCTAATCTCTTACAGATTGCGCGTACCGTACTTTT

>TWYL_1

GAGCGACCCCATAGGCTTGTGCTTTCGCGTCTCTCTAAAATTGCAGTCATGTTAAAATCTTGGTTTATTCAATCATCAGGAGCTCCCAAGCACACAGATTATCTATAAATGGATAATAGAAGGCTTGTTATTCAACAGTATAACATGCCTTATATGCCCGTGTCAACCAAAGCAATATCAACAGACTTCTTTCTATTCTTTTCTTTCCATACGGAAAGATCCATCCGAGCGATCTGTGAATGAAGTGAGTAGAATACATATTCTAGATGGTTTAGTTTAATTAAATTTGATAAAATTACGTATTTAATGTATGGTAGTGGGTTGCCCGGGACTCGAACCCGGAACTAGTCGGATGGAGTAGATAATCTCTTTGTTAATAGGAAAAAAAGAATCTCTCCCCAAACCGTGCTTGCATTTTTCATTGCACACGGCTTTCTCTGTGTATACATCTAAAACGAAGTTCCCTAGAAAATAGAACTCTAATAAGAAACTTGAATACTCAGTCGATTCAACCACTAGTACATGAAATGAGCATTTCATTAATAGATGAAATTTGGAATTTTTATATTTACGAATTAAATCTCGTCATTAGCATTAGTTGTTATGATTCCCATTTACTCCACACCCAATTATGAATTGGGTACCAGACCATTGATACGGATAATATCCAAATACCAAATCCGTTCGCTATGTAAACGGGTTCTTCGGAAGATGAAAGAAACAATTTCTTGTTCCTCTGTAAGGAACTCTTCCAATAGTTTCGAACCTAATCTCTTACAGATTGCGCGTACCGTACTTTT

>QYBSZ_1

GAGCGACCCCATAGGCTTGTGCTTTCGCGTCTCTCTAAAATTGCAGTCATGTTAAAATCTTGGTTTATTCAATCATCAGGAGCTCCCAAGCACACAGATTATCTATAAATGGATAATAGAAGGCTTGTTATTCAACAGTATAACATGCCTTATATGCCCGTGTCAACCAAAGCAATATCAACAGACTTCTTTCTATTCTTTTCTTTCCATACGGAAAGATCCATCCGAGCGATCTGTGAATGAAGTGAGTAGAATACATATTCTAGATGGTTTAGTTTAATTAAATTTGATAAAATTACGTATTTAATGTATGGTAGTGGGTTGCCCGGGACTCGAACCCGGAACTAGTCGGATGGAGTAGATAATCTCTTTGTTAATAGGAAAAAAAGAATCTCTCCCCAAACCGTGCTTGCATTTTTCATTGCACACGGCTTTCTCTGTGTATACATCTAAAACGAAGTTCCCTAGAAAATAGAACTCTAATAAGAAACTTGAATACTCAGTCGATTCAACCACTAGTACATGAAATGAGCATTTCATTAATAGATGAAATTTGGAATTTTTATATTTACGAATTAAATCTCGTCATTAGCATTAGTTGTTATGATTCCCATTTACTCCACACCCAATTATGAATTGGGTACCAGACCATTGATACGGATAATATCCAAATACCAAATCCGTTCGCTATGTAAACGGGTTCTTCGGAAGATGAAAGAAACAATTTCTTGTTCCTCTGTAAGGAACTCTTCCAATAGTTTCGAACCTAATCTCTTACAGATTGCGCGTACCGTACTTTT

>YNTC_1

GAGCGACCCCATAGGCTTGTGCTTTCGCGTCTCTCTAAAATTGCAGTCATGTTAAAATCTTGGTTTATTCAATCATCAGGAGCTCCCAAGCACACAGATTATCTATAAATGGATAATAGAAGGCTTGTTATTCAACAGTATAACATGCCTTATATGCCCGTGTCAACCAAAGCAATATCAACAGACTTCTTTCTATTCTTTTCTTTCCATACGGAAAGATCCATCCGAGCGATCTGTGAATGAAGTGAGTAGAATACATATTCTAGATGGTTTAGTTTAATTAAATTTGATAAAATTACGTATTTAATGTATGGTAGTGGGTTGCCCGGGACTCGAACCCGGAACTAGTCGGATGGAGTAGATAATCTCTTTGTTAATAGGAAAAAAAGAATCTCTCCCCAAACCGTGCTTGCATTTTTCATTGCACACGGCTTTCTCTGTGTATACATCTAAAACGAAGTTCCCTAGAAAATAGAACTCTAATAAGAAACTTGAATACTCAGTCGATTCAACCACTAGTACATGAAATGAGCATTTCATTAATAGATGAAATTTGGAATTTTTATATTTACGAATTAAATCTCGTCATTAGCATTAGTTGTTATGATTCCCATTTACTCCACACCCAATTATGAATTGGGTACCAGACCATTGATACGGATAATATCCAAATACCAAATCCGTTCGCTATGTAAACGGGTTCTTCGGAAGATGAAAGAAACAATTTCTTGTTCCTCTGTAAGGAACTCTTCCAATAGTTTCGAACCTAATCTCTTACAGATTGCGCGTACCGTACTTTT

*psbA-trnH* (pairwise identity: 100%)


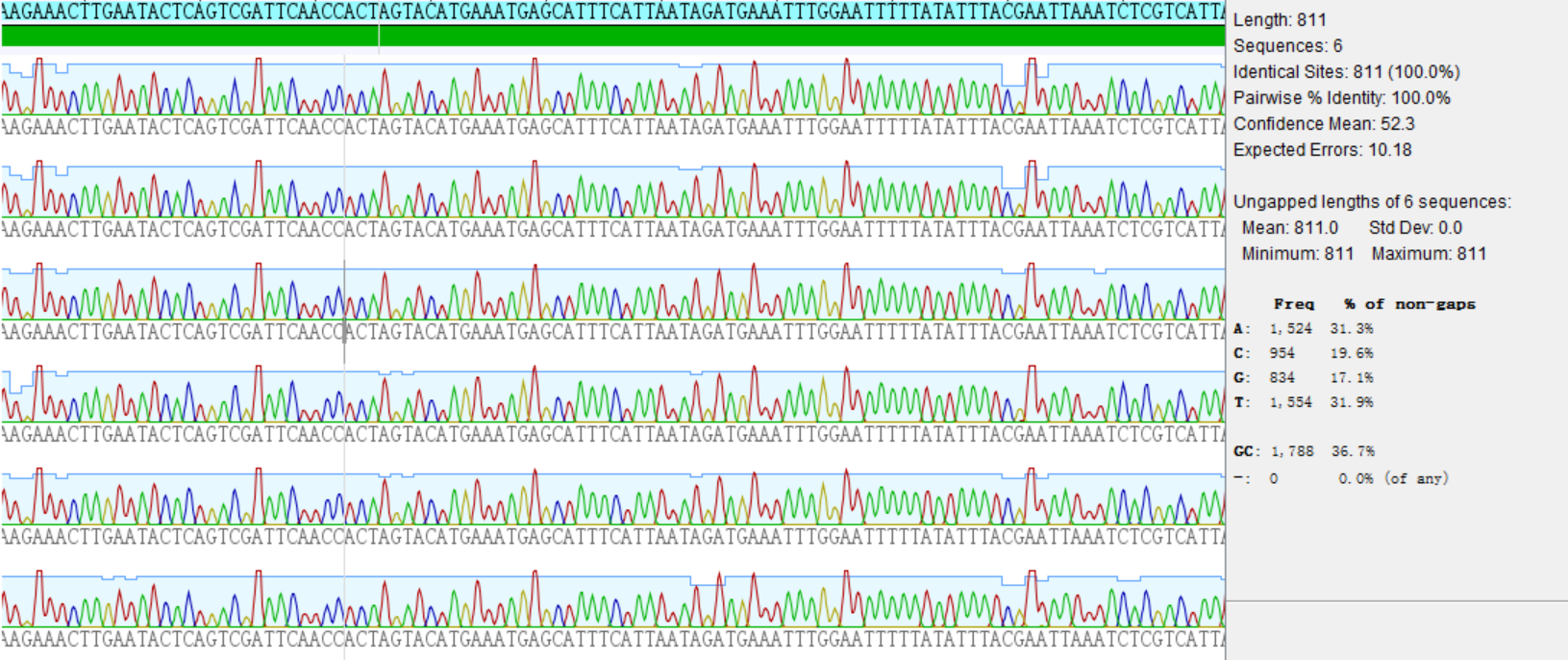


>NXQZS_1

TCCACTTGGCTACATCCGCCCCTAGTCTGACTCAATTAACAGAAGAGTCATGTCATATTTCGTTTTTAGCCTTCATCACATTCTTTATCACTCTCCGTTTCTTCTGAAACGATATTTCATTATTTAATGTTATCTATGCCTTGGCATAGGAGGTAAACCCCGTAAAAAAGTAAAACGATTCCGTAGAACTTTTCTCGTTATAAAAAATTTTCTTTATATTATTTATATATATATTATAAATTTATAATAGTCGATATACAATAACTATCTTAGAAAATAGTTATATCTTATAGGTCACTCAGCGATGAACCGCGCAGCAAACATGCTGCTTGCTGTATTTCTTTTTTTTTTTTTAAGTAAAGATGGAAGAACGGGCCAATACCCCACCTTTTTGGTTGGGTATTGGTCCTTCAACGACTCGTATACACTGACATCAAATATTATCCATTGGTAGATGGAGCTTCG

>GDSYD_1

TCCACTTGGCTACATCCGCCCCTAGTCTGACTCAATTAACAGAAGAGTCATGTCATATTTCGTTTTTAGCCTTCATCACATTCTTTATCACTCTCCGTTTCTTCTGAAACGATATTTCATTATTTAATGTTATCTATGCCTTGGCATAGGAGGTAAACCCCGTAAAAAAGTAAAACGATTCCGTAGAACTTTTCTCGTTATAAAAAATTTTCTTTATATTATTTATATATATATTATAAATTTATAATAGTCGATATACAATAACTATCTTAGAAAATAGTTATATCTTATAGGTCACTCAGCGATGAACCGCGCAGCAAACATGCTGCTTGCTGTATTTCTTTTTTTTTTTTTAAGTAAAGATGGAAGAACGGGCCAATACCCCACCTTTTTGGTTGGGTATTGGTCCTTCAACGACTCGTATACACTGACATCAAATATTATCCATTGGTAGATGGAGCTTCG

>NDXFS_1

TCCACTTGGCTACATCCGCCCCTAGTCTGACTCAATTAACAGAAGAGTCATGTCATATTTCGTTTTTAGCCTTCATCACATTCTTTATCACTCTCCGTTTCTTCTGAAACGATATTTCATTATTTAATGTTATCTATGCCTTGGCATAGGAGGTAAACCCCGTAAAAAAGTAAAACGATTCCGTAGAACTTTTCTCGTTATAAAAAATTTTCTTTATATTATTTATATATATATTATAAATTTATAATAGTCGATATACAATAACTATCTTAGAAAATAGTTATATCTTATAGGTCACTCAGCGATGAACCGCGCAGCAAACATGCTGCTTGCTGTATTTCTTTTTTTTTTTTTAAGTAAAGATGGAAGAACGGGCCAATACCCCACCTTTTTGGTTGGGTATTGGTCCTTCAACGACTCGTATACACTGACATCAAATATTATCCATTGGTAGATGGAGCTTCG

>TWYL_1

TCCACTTGGCTACATCCGCCCCTAGTCTGACTCAATTAACAGAAGAGTCATGTCATATTTCGTTTTTAGCCTTCATCACATTCTTTATCACTCTCCGTTTCTTCTGAAACGATATTTCATTATTTAATGTTATCTATGCCTTGGCATAGGAGGTAAACCCCGTAAAAAAGTAAAACGATTCCGTAGAACTTTTCTCGTTATAAAAAATTTTCTTTATATTATTTATATATATATTATAAATTTATAATAGTCGATATACAATAACTATCTTAGAAAATAGTTATATCTTATAGGTCACTCAGCGATGAACCGCGCAGCAAACATGCTGCTTGCTGTATTTCTTTTTTTTTTTTTAAGTAAAGATGGAAGAACGGGCCAATACCCCACCTTTTTGGTTGGGTATTGGTCCTTCAACGACTCGTATACACTGACATCAAATATTATCCATTGGTAGATGGAGCTTCG

>QYBSZ_1

TCCACTTGGCTACATCCGCCCCTAGTCTGACTCAATTAACAGAAGAGTCATGTCATATTTCGTTTTTAGCCTTCATCACATTCTTTATCACTCTCCGTTTCTTCTGAAACGATATTTCATTATTTAATGTTATCTATGCCTTGGCATAGGAGGTAAACCCCGTAAAAAAGTAAAACGATTCCGTAGAACTTTTCTCGTTATAAAAAATTTTCTTTATATTATTTATATATATATTATAAATTTATAATAGTCGATATACAATAACTATCTTAGAAAATAGTTATATCTTATAGGTCACTCAGCGATGAACCGCGCAGCAAACATGCTGCTTGCTGTATTTCTTTTTTTTTTTTTAAGTAAAGATGGAAGAACGGGCCAATACCCCACCTTTTTGGTTGGGTATTGGTCCTTCAACGACTCGTATACACTGACATCAAATATTATCCATTGGTAGATGGAGCTTCG

>YNTC_1

TCCACTTGGCTACATCCGCCCCTAGTCTGACTCAATTAACAGAAGAGTCATGTCATATTTCGTTTTTAGCCTTCATCACATTCTTTATCACTCTCCGTTTCTTCTGAAACGATATTTCATTATTTAATGTTATCTATGCCTTGGCATAGGAGGTAAACCCCGTAAAAAAGTAAAACGATTCCGTAGAACTTTTCTCGTTATAAAAAATTTTCTTTATATTATTTATATATATATTATAAATTTATAATAGTCGATATACAATAACTATCTTAGAAAATAGTTATATCTTATAGGTCACTCAGCGATGAACCGCGCAGCAAACATGCTGCTTGCTGTATTTCTTTTTTTTTTTTTAAGTAAAGATGGAAGAACGGGCCAATACCCCACCTTTTTGGTTGGGTATTGGTCCTTCAACGACTCGTATACACTGACATCAAATATTATCCATTGGTAGATGGAGCTTCG

*3’trnG-5’trnG* (pairwise identity: 100%)


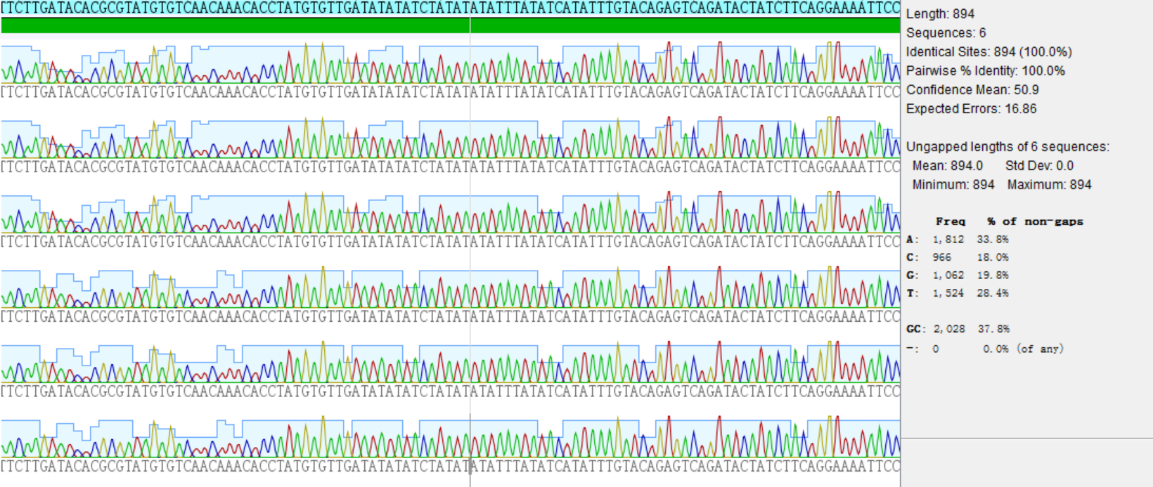


>NXQZS_1

GAAAAATAAAGATTTTACCATTTTCATTTTTAATATAAAAAGAAAGATGAGATAAGTAGTGCATTAAGACAAGAACCCTCGATTCAAACATTGAACTGAACATTCTTTGATAGACTCCGTGAATCCGGATCGGATCACCTCATTTCTTCATTCTGCCCCTCCAGCTGTGGAGCGGATACGGCATTCTGATCCGCACAATATCAAATTGCATTGTAGGCATGACGAGATTAATTGTTTTGATAACGAAGGAATCAGAACCTTATTACAAATGAATTGATTTTCTGCTAATTCCTTTCTTTTCTAAAAACCACTTCGTTTCTTGGTGTCAAAAAATAGGATGGTACAAAGGTTGAGAATCTATTCCCTTTTTCTCCCCCAACAGGTCTTGGAGATTTGTAATGCTTACTCTCAAACTGTTCGTTTATACGGTGGTGATATTCTTTGTTTCGCTCTTCATCTTCGGATTCCTATCTAATGACCCAGGACGTAATCCTGGACGCGAGGAATAAAGAATAATGTATTTTTCCTTTCCTTTTTTTATTTCTCAATCCATCTTCTTAACTTAGGATTTGATCTATTCCATAGATTTCATTTAGATAAATGAAACCATCAATTCAACCAAGCCAATCAGGATTTCTAGAATTATAAAGATAA

>GDSYD_1

GAAAAATAAAGATTTTACCATTTTCATTTTTAATATAAAAAGAAAGATGAGATAAGTAGTGCATTAAGACAAGAACCCTCGATTCAAACATTGAACTGAACATTCTTTGATAGACTCCGTGAATCCGGATCGGATCACCTCATTTCTTCATTCTGCCCCTCCAGCTGTGGAGCGGATACGGCATTCTGATCCGCACAATATCAAATTGCATTGTAGGCATGACGAGATTAATTGTTTTGATAACGAAGGAATCAGAACCTTATTACAAATGAATTGATTTTCTGCTAATTCCTTTCTTTTCTAAAAACCACTTCGTTTCTTGGTGTCAAAAAATAGGATGGTACAAAGGTTGAGAATCTATTCCCTTTTTCTCCCCCAACAGGTCTTGGAGATTTGTAATGCTTACTCTCAAACTGTTCGTTTATACGGTGGTGATATTCTTTGTTTCGCTCTTCATCTTCGGATTCCTATCTAATGACCCAGGACGTAATCCTGGACGCGAGGAATAAAGAATAATGTATTTTTCCTTTCCTTTTTTTATTTCTCAATCCATCTTCTTAACTTAGGATTTGATCTATTCCATAGATTTCATTTAGATAAATGAAACCATCAATTCAACCAAGCCAATCAGGATTTCTAGAATTATAAAGATAA

>NDXFS_1

GAAAAATAAAGATTTTACCATTTTCATTTTTAATATAAAAAGAAAGATGAGATAAGTAGTGCATTAAGACAAGAACCCTCGATTCAAACATTGAACTGAACATTCTTTGATAGACTCCGTGAATCCGGATCGGATCACCTCATTTCTTCATTCTGCCCCTCCAGCTGTGGAGCGGATACGGCATTCTGATCCGCACAATATCAAATTGCATTGTAGGCATGACGAGATTAATTGTTTTGATAACGAAGGAATCAGAACCTTATTACAAATGAATTGATTTTCTGCTAATTCCTTTCTTTTCTAAAAACCACTTCGTTTCTTGGTGTCAAAAAATAGGATGGTACAAAGGTTGAGAATCTATTCCCTTTTTCTCCCCCAACAGGTCTTGGAGATTTGTAATGCTTACTCTCAAACTGTTCGTTTATACGGTGGTGATATTCTTTGTTTCGCTCTTCATCTTCGGATTCCTATCTAATGACCCAGGACGTAATCCTGGACGCGAGGAATAAAGAATAATGTATTTTTCCTTTCCTTTTTTTATTTCTCAATCCATCTTCTTAACTTAGGATTTGATCTATTCCATAGATTTCATTTAGATAAATGAAACCATCAATTCAACCAAGCCAATCAGGATTTCTAGAATTATAAAGATAA

>TWYL_1

GAAAAATAAAGATTTTACCATTTTCATTTTTAATATAAAAAGAAAGATGAGATAAGTAGTGCATTAAGACAAGAACCCTCGATTCAAACATTGAACTGAACATTCTTTGATAGACTCCGTGAATCCGGATCGGATCACCTCATTTCTTCATTCTGCCCCTCCAGCTGTGGAGCGGATACGGCATTCTGATCCGCACAATATCAAATTGCATTGTAGGCATGACGAGATTAATTGTTTTGATAACGAAGGAATCAGAACCTTATTACAAATGAATTGATTTTCTGCTAATTCCTTTCTTTTCTAAAAACCACTTCGTTTCTTGGTGTCAAAAAATAGGATGGTACAAAGGTTGAGAATCTATTCCCTTTTTCTCCCCCAACAGGTCTTGGAGATTTGTAATGCTTACTCTCAAACTGTTCGTTTATACGGTGGTGATATTCTTTGTTTCGCTCTTCATCTTCGGATTCCTATCTAATGACCCAGGACGTAATCCTGGACGCGAGGAATAAAGAATAATGTATTTTTCCTTTCCTTTTTTTATTTCTCAATCCATCTTCTTAACTTAGGATTTGATCTATTCCATAGATTTCATTTAGATAAATGAAACCATCAATTCAACCAAGCCAATCAGGATTTCTAGAATTATAAAGATAA

>QYBSZ_1

GAAAAATAAAGATTTTACCATTTTCATTTTTAATATAAAAAGAAAGATGAGATAAGTAGTGCATTAAGACAAGAACCCTCGATTCAAACATTGAACTGAACATTCTTTGATAGACTCCGTGAATCCGGATCGGATCACCTCATTTCTTCATTCTGCCCCTCCAGCTGTGGAGCGGATACGGCATTCTGATCCGCACAATATCAAATTGCATTGTAGGCATGACGAGATTAATTGTTTTGATAACGAAGGAATCAGAACCTTATTACAAATGAATTGATTTTCTGCTAATTCCTTTCTTTTCTAAAAACCACTTCGTTTCTTGGTGTCAAAAAATAGGATGGTACAAAGGTTGAGAATCTATTCCCTTTTTCTCCCCCAACAGGTCTTGGAGATTTGTAATGCTTACTCTCAAACTGTTCGTTTATACGGTGGTGATATTCTTTGTTTCGCTCTTCATCTTCGGATTCCTATCTAATGACCCAGGACGTAATCCTGGACGCGAGGAATAAAGAATAATGTATTTTTCCTTTCCTTTTTTTATTTCTCAATCCATCTTCTTAACTTAGGATTTGATCTATTCCATAGATTTCATTTAGATAAATGAAACCATCAATTCAACCAAGCCAATCAGGATTTCTAGAATTATAAAGATAA

>YNTC_1

GAAAAATAAAGATTTTACCATTTTCATTTTTAATATAAAAAGAAAGATGAGATAAGTAGTGCATTAAGACAAGAACCCTCGATTCAAACATTGAACTGAACATTCTTTGATAGACTCCGTGAATCCGGATCGGATCACCTCATTTCTTCATTCTGCCCCTCCAGCTGTGGAGCGGATACGGCATTCTGATCCGCACAATATCAAATTGCATTGTAGGCATGACGAGATTAATTGTTTTGATAACGAAGGAATCAGAACCTTATTACAAATGAATTGATTTTCTGCTAATTCCTTTCTTTTCTAAAAACCACTTCGTTTCTTGGTGTCAAAAAATAGGATGGTACAAAGGTTGAGAATCTATTCCCTTTTTCTCCCCCAACAGGTCTTGGAGATTTGTAATGCTTACTCTCAAACTGTTCGTTTATACGGTGGTGATATTCTTTGTTTCGCTCTTCATCTTCGGATTCCTATCTAATGACCCAGGACGTAATCCTGGACGCGAGGAATAAAGAATAATGTATTTTTCCTTTCCTTTTTTTATTTCTCAATCCATCTTCTTAACTTAGGATTTGATCTATTCCATAGATTTCATTTAGATAAATGAAACCATCAATTCAACCAAGCCAATCAGGATTTCTAGAATTATAAAGATAA

*psbM-trnD* (pairwise identity: 100%)


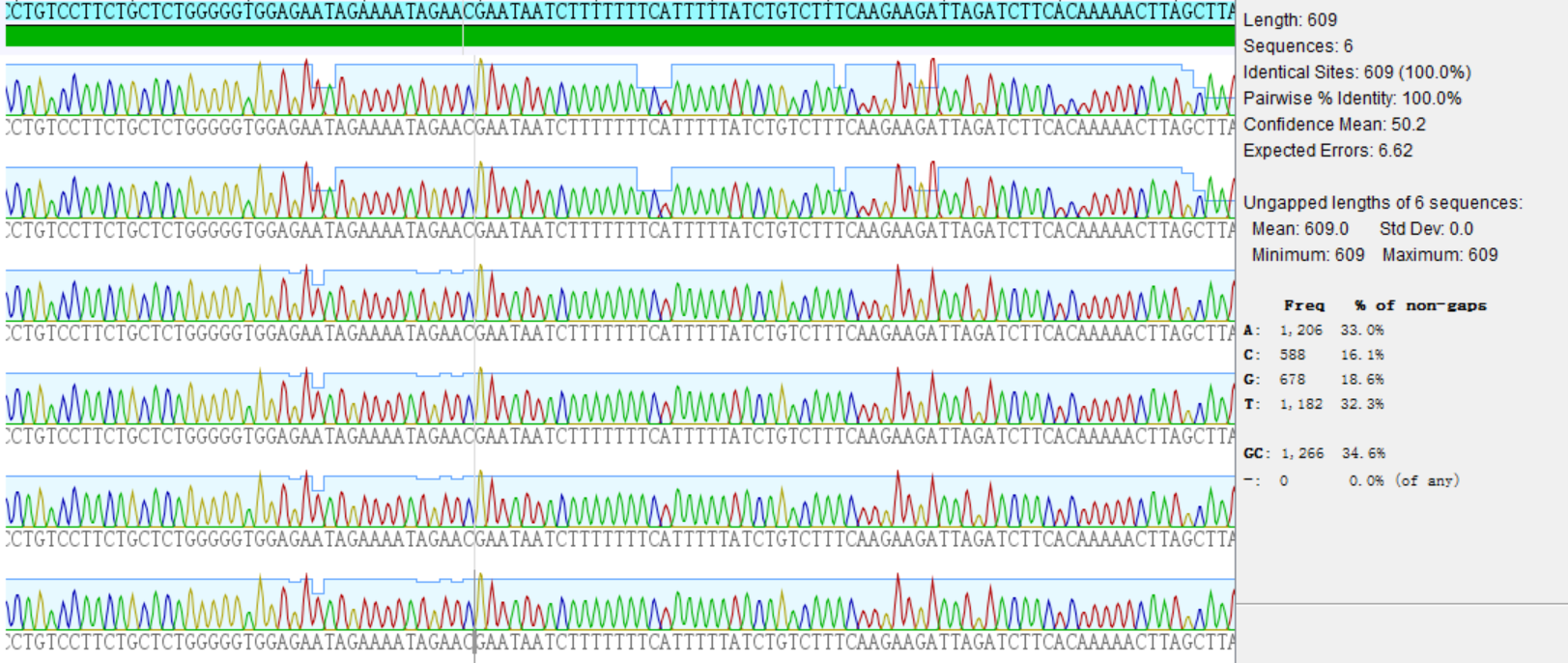


>NXQZS_1

TTTTTACTTCTTTGGTAATCTACCAAGGGTTATAGTCATAGTGATCCTCCTATTTAACTACTTCAACCATTTTCGAACACCTCATATCATTATCTGGGTATATGACGGTTCGATCGTCTAGAGAAGATCCCCCGTACACTACCCTTTCCAAAATATTGGGTTTCGAAGATACAATACGGATCCTTCCTTTCTACTGTACCAAAGCGACCTAGCCATGCTAGCTCGCCTCAATTATTCTTTCCTATTGAACCATGCGATACCCCAATTCTCTTATGCCCTATCAATCTGATTCAGTTTATGAAATAACTGACAAGGAGCAACCGATCCCTTCTTTCGATACCACTTGCTTCCCATTCCCAGATCTATTGCCCTCTCCCTTCTATCAACTGATTTGATTGTTGGATCTTAGTGGTGATATTGAGAAAAGATCAACCATTTATGGTATGGATTATCGCAATTTCTGTGTATATCTGTTCAATGCAATTACAATGAATAAATTCTCCTAATTTATATTTCTGTGTATACTATATTACTATAATATGATCTATACTACTATTTATTATAATCGAATAAAGTAGTTAAGTTAAAGTTAAGTTAATAGTAATAGTAGCATATGGTTTTGTTACTGTTTCATTTTTTTTCTCCCTCGGATGAATCGAAAATCTCAGAGTATTTATTTTCACTATTTTCACCCGGAGTATATGTTTTCATGGGTCGAACCAAAAAAATAAACTTGGAAAATTTGCCTCTTCCTCTTTAACTTTTTTGTCTGCTATAGGAAACTAGGGAATGAACGTTCCTTTTCATGATTCGGATTTATTCGCCATTGGCGAAACCAAAATATCGGATGCATCGATAGGGA

>GDSYD_1

TTTTTACTTCTTTGGTAATCTACCAAGGGTTATAGTCATAGTGATCCTCCTATTTAACTACTTCAACCATTTTCGAACACCTCATATCATTATCTGGGTATATGACGGTTCGATCGTCTAGAGAAGATCCCCCGTACACTACCCTTTCCAAAATATTGGGTTTCGAAGATACAATACGGATCCTTCCTTTCTACTGTACCAAAGCGACCTAGCCATGCTAGCTCGCCTCAATTATTCTTTCCTATTGAACCATGCGATACCCCAATTCTCTTATGCCCTATCAATCTGATTCAGTTTATGAAATAACTGACAAGGAGCAACCGATCCCTTCTTTCGATACCACTTGCTTCCCATTCCCAGATCTATTGCCCTCTCCCTTCTATCAACTGATTTGATTGTTGGATCTTAGTGGTGATATTGAGAAAAGATCAACCATTTATGGTATGGATTATCGCAATTTCTGTGTATATCTGTTCAATGCAATTACAATGAATAAATTCTCCTAATTTATATTTCTGTGTATACTATATTACTATAATATGATCTATACTACTATTTATTATAATCGAATAAAGTAGTTAAGTTAAAGTTAAGTTAATAGTAATAGTAGCATATGGTTTTGTTACTGTTTCATTTTTTTTCTCCCTCGGATGAATCGAAAATCTCAGAGTATTTATTTTCACTATTTTCACCCGGAGTATATGTTTTCATGGGTCGAACCAAAAAAATAAACTTGGAAAATTTGCCTCTTCCTCTTTAACTTTTTTGTCTGCTATAGGAAACTAGGGAATGAACGTTCCTTTTCATGATTCGGATTTATTCGCCATTGGCGAAACCAAAATATCGGATGCATCGATAGGGA

>NDXFS_1

TTTTTACTTCTTTGGTAATCTACCAAGGGTTATAGTCATAGTGATCCTCCTATTTAACTACTTCAACCATTTTCGAACACCTCATATCATTATCTGGGTATATGACGGTTCGATCGTCTAGAGAAGATCCCCCGTACACTACCCTTTCCAAAATATTGGGTTTCGAAGATACAATACGGATCCTTCCTTTCTACTGTACCAAAGCGACCTAGCCATGCTAGCTCGCCTCAATTATTCTTTCCTATTGAACCATGCGATACCCCAATTCTCTTATGCCCTATCAATCTGATTCAGTTTATGAAATAACTGACAAGGAGCAACCGATCCCTTCTTTCGATACCACTTGCTTCCCATTCCCAGATCTATTGCCCTCTCCCTTCTATCAACTGATTTGATTGTTGGATCTTAGTGGTGATATTGAGAAAAGATCAACCATTTATGGTATGGATTATCGCAATTTCTGTGTATATCTGTTCAATGCAATTACAATGAATAAATTCTCCTAATTTATATTTCTGTGTATACTATATTACTATAATATGATCTATACTACTATTTATTATAATCGAATAAAGTAGTTAAGTTAAAGTTAAGTTAATAGTAATAGTAGCATATGGTTTTGTTACTGTTTCATTTTTTTTCTCCCTCGGATGAATCGAAAATCTCAGAGTATTTATTTTCACTATTTTCACCCGGAGTATATGTTTTCATGGGTCGAACCAAAAAAATAAACTTGGAAAATTTGCCTCTTCCTCTTTAACTTTTTTGTCTGCTATAGGAAACTAGGGAATGAACGTTCCTTTTCATGATTCGGATTTATTCGCCATTGGCGAAACCAAAATATCGGATGCATCGATAGGGA

>TWYL_1

TTTTTACTTCTTTGGTAATCTACCAAGGGTTATAGTCATAGTGATCCTCCTATTTAACTACTTCAACCATTTTCGAACACCTCATATCATTATCTGGGTATATGACGGTTCGATCGTCTAGAGAAGATCCCCCGTACACTACCCTTTCCAAAATATTGGGTTTCGAAGATACAATACGGATCCTTCCTTTCTACTGTACCAAAGCGACCTAGCCATGCTAGCTCGCCTCAATTATTCTTTCCTATTGAACCATGCGATACCCCAATTCTCTTATGCCCTATCAATCTGATTCAGTTTATGAAATAACTGACAAGGAGCAACCGATCCCTTCTTTCGATACCACTTGCTTCCCATTCCCAGATCTATTGCCCTCTCCCTTCTATCAACTGATTTGATTGTTGGATCTTAGTGGTGATATTGAGAAAAGATCAACCATTTATGGTATGGATTATCGCAATTTCTGTGTATATCTGTTCAATGCAATTACAATGAATAAATTCTCCTAATTTATATTTCTGTGTATACTATATTACTATAATATGATCTATACTACTATTTATTATAATCGAATAAAGTAGTTAAGTTAAAGTTAAGTTAATAGTAATAGTAGCATATGGTTTTGTTACTGTTTCATTTTTTTTCTCCCTCGGATGAATCGAAAATCTCAGAGTATTTATTTTCACTATTTTCACCCGGAGTATATGTTTTCATGGGTCGAACCAAAAAAATAAACTTGGAAAATTTGCCTCTTCCTCTTTAACTTTTTTGTCTGCTATAGGAAACTAGGGAATGAACGTTCCTTTTCATGATTCGGATTTATTCGCCATTGGCGAAACCAAAATATCGGATGCATCGATAGGGA

>QYBSZ_1

TTTTTACTTCTTTGGTAATCTACCAAGGGTTATAGTCATAGTGATCCTCCTATTTAACTACTTCAACCATTTTCGAACACCTCATATCATTATCTGGGTATATGACGGTTCGATCGTCTAGAGAAGATCCCCCGTACACTACCCTTTCCAAAATATTGGGTTTCGAAGATACAATACGGATCCTTCCTTTCTACTGTACCAAAGCGACCTAGCCATGCTAGCTCGCCTCAATTATTCTTTCCTATTGAACCATGCGATACCCCAATTCTCTTATGCCCTATCAATCTGATTCAGTTTATGAAATAACTGACAAGGAGCAACCGATCCCTTCTTTCGATACCACTTGCTTCCCATTCCCAGATCTATTGCCCTCTCCCTTCTATCAACTGATTTGATTGTTGGATCTTAGTGGTGATATTGAGAAAAGATCAACCATTTATGGTATGGATTATCGCAATTTCTGTGTATATCTGTTCAATGCAATTACAATGAATAAATTCTCCTAATTTATATTTCTGTGTATACTATATTACTATAATATGATCTATACTACTATTTATTATAATCGAATAAAGTAGTTAAGTTAAAGTTAAGTTAATAGTAATAGTAGCATATGGTTTTGTTACTGTTTCATTTTTTTTCTCCCTCGGATGAATCGAAAATCTCAGAGTATTTATTTTCACTATTTTCACCCGGAGTATATGTTTTCATGGGTCGAACCAAAAAAATAAACTTGGAAAATTTGCCTCTTCCTCTTTAACTTTTTTGTCTGCTATAGGAAACTAGGGAATGAACGTTCCTTTTCATGATTCGGATTTATTCGCCATTGGCGAAACCAAAATATCGGATGCATCGATAGGGA

>YNTC_1

TTTTTACTTCTTTGGTAATCTACCAAGGGTTATAGTCATAGTGATCCTCCTATTTAACTACTTCAACCATTTTCGAACACCTCATATCATTATCTGGGTATATGACGGTTCGATCGTCTAGAGAAGATCCCCCGTACACTACCCTTTCCAAAATATTGGGTTTCGAAGATACAATACGGATCCTTCCTTTCTACTGTACCAAAGCGACCTAGCCATGCTAGCTCGCCTCAATTATTCTTTCCTATTGAACCATGCGATACCCCAATTCTCTTATGCCCTATCAATCTGATTCAGTTTATGAAATAACTGACAAGGAGCAACCGATCCCTTCTTTCGATACCACTTGCTTCCCATTCCCAGATCTATTGCCCTCTCCCTTCTATCAACTGATTTGATTGTTGGATCTTAGTGGTGATATTGAGAAAAGATCAACCATTTATGGTATGGATTATCGCAATTTCTGTGTATATCTGTTCAATGCAATTACAATGAATAAATTCTCCTAATTTATATTTCTGTGTATACTATATTACTATAATATGATCTATACTACTATTTATTATAATCGAATAAAGTAGTTAAGTTAAAGTTAAGTTAATAGTAATAGTAGCATATGGTTTTGTTACTGTTTCATTTTTTTTCTCCCTCGGATGAATCGAAAATCTCAGAGTATTTATTTTCACTATTTTCACCCGGAGTATATGTTTTCATGGGTCGAACCAAAAAAATAAACTTGGAAAATTTGCCTCTTCCTCTTTAACTTTTTTGTCTGCTATAGGAAACTAGGGAATGAACGTTCCTTTTCATGATTCGGATTTATTCGCCATTGGCGAAACCAAAATATCGGATGCATCGATAGGGA

*ITS1-ITS4* (pairwise identity: 100%)


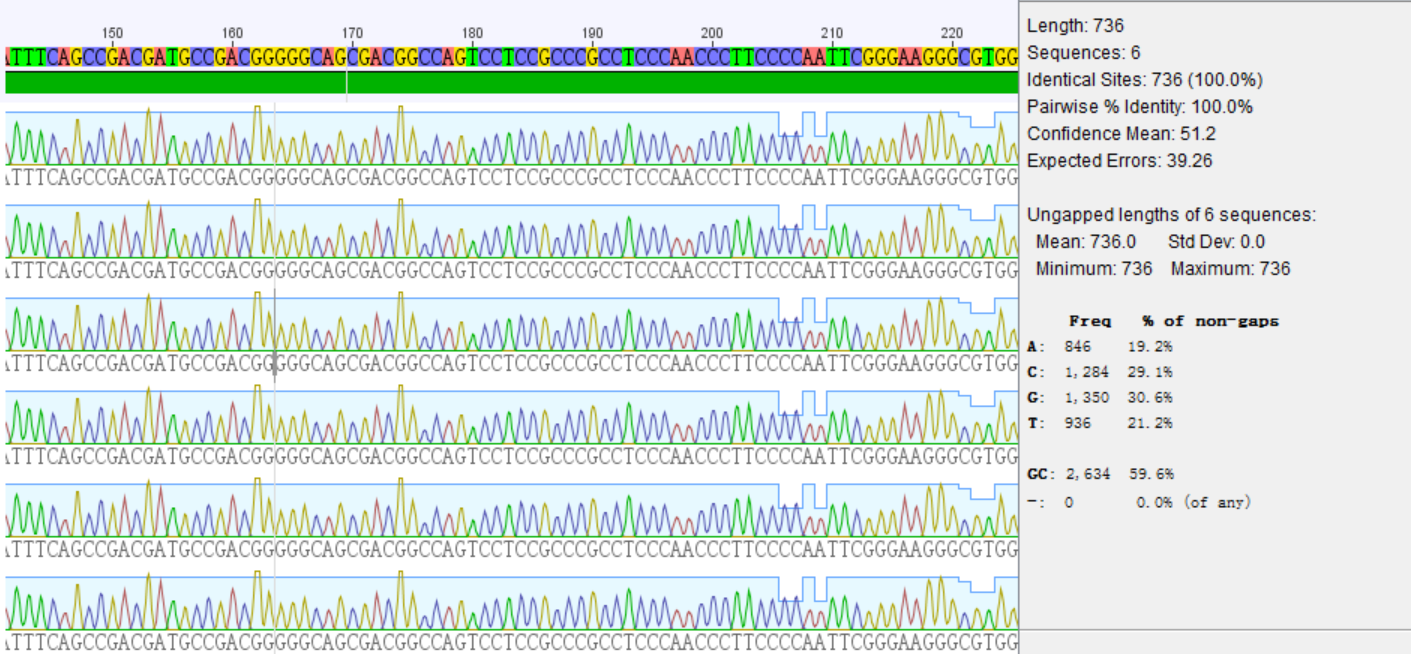


>NXQZS_1

TCGCTAGGCTGGAGGCATTGCCCCCTCAGAGTTCCTCTTGCACCGGGGCATCGCATGGTTGGACGGCTAAAGGTGTTCCACCGCTTGCCGCAACTTAACGAGACGCGGGCCTAGCATTTCAGCCGACGATGCCGACGGGGGCAGCGACGGCCAGTCCTCCGCCCGCCTCCCAACCCTTCCCCAATTCGGGAAGGGCGTGGGAGGAGAGCGACGCATAGCGTGACGCCCAGGCAGGTGTGCCCTCGACCGGGTGGTCTCGGGCGCAACTTGCGTTCAAAGACTCGATGATTCACGGGATTCTGCAATTCACATCAAGTATCGCATTTCGCTACGTTCTTCATCGTTGCGGGAGCCAAGATATCCGTTGCCGAGAGTCGTTTAGATAGATAGAGGCAAAGGTCACCCCCCATTGCCATGGGTGCGGGCCGATGCTCTTTGTTGGTATTGTCCTTGACGCCTGTGGCGCCGTTTTGCTTGTTACGGCCGCAACGACATCCCCACCACGCGTAACCCGACCGGGACCCGAGGGTCCTAGATCAAGGCGGCGAGGGGAGGGGAGCGCAACGACCGGTCGGGGGACGCCCACCCGGGGGCGGGCAATCCGACGACGAGTGATGATAACGTGTTCGCGGGTGTGTCGATCAGGATTGGCAATGATCCTTCCGCAGGTTCACCTACGGAAACCT

>GDSYD_1

TCGCTAGGCTGGAGGCATTGCCCCCTCAGAGTTCCTCTTGCACCGGGGCATCGCATGGTTGGACGGCTAAAGGTGTTCCACCGCTTGCCGCAACTTAACGAGACGCGGGCCTAGCATTTCAGCCGACGATGCCGACGGGGGCAGCGACGGCCAGTCCTCCGCCCGCCTCCCAACCCTTCCCCAATTCGGGAAGGGCGTGGGAGGAGAGCGACGCATAGCGTGACGCCCAGGCAGGTGTGCCCTCGACCGGGTGGTCTCGGGCGCAACTTGCGTTCAAAGACTCGATGATTCACGGGATTCTGCAATTCACATCAAGTATCGCATTTCGCTACGTTCTTCATCGTTGCGGGAGCCAAGATATCCGTTGCCGAGAGTCGTTTAGATAGATAGAGGCAAAGGTCACCCCCCATTGCCATGGGTGCGGGCCGATGCTCTTTGTTGGTATTGTCCTTGACGCCTGTGGCGCCGTTTTGCTTGTTACGGCCGCAACGACATCCCCACCACGCGTAACCCGACCGGGACCCGAGGGTCCTAGATCAAGGCGGCGAGGGGAGGGGAGCGCAACGACCGGTCGGGGGACGCCCACCCGGGGGCGGGCAATCCGACGACGAGTGATGATAACGTGTTCGCGGGTGTGTCGATCAGGATTGGCAATGATCCTTCCGCAGGTTCACCTACGGAAACCT

>NDXFS_1

TCGCTAGGCTGGAGGCATTGCCCCCTCAGAGTTCCTCTTGCACCGGGGCATCGCATGGTTGGACGGCTAAAGGTGTTCCACCGCTTGCCGCAACTTAACGAGACGCGGGCCTAGCATTTCAGCCGACGATGCCGACGGGGGCAGCGACGGCCAGTCCTCCGCCCGCCTCCCAACCCTTCCCCAATTCGGGAAGGGCGTGGGAGGAGAGCGACGCATAGCGTGACGCCCAGGCAGGTGTGCCCTCGACCGGGTGGTCTCGGGCGCAACTTGCGTTCAAAGACTCGATGATTCACGGGATTCTGCAATTCACATCAAGTATCGCATTTCGCTACGTTCTTCATCGTTGCGGGAGCCAAGATATCCGTTGCCGAGAGTCGTTTAGATAGATAGAGGCAAAGGTCACCCCCCATTGCCATGGGTGCGGGCCGATGCTCTTTGTTGGTATTGTCCTTGACGCCTGTGGCGCCGTTTTGCTTGTTACGGCCGCAACGACATCCCCACCACGCGTAACCCGACCGGGACCCGAGGGTCCTAGATCAAGGCGGCGAGGGGAGGGGAGCGCAACGACCGGTCGGGGGACGCCCACCCGGGGGCGGGCAATCCGACGACGAGTGATGATAACGTGTTCGCGGGTGTGTCGATCAGGATTGGCAATGATCCTTCCGCAGGTTCACCTACGGAAACCT

>TWYL_1

TCGCTAGGCTGGAGGCATTGCCCCCTCAGAGTTCCTCTTGCACCGGGGCATCGCATGGTTGGACGGCTAAAGGTGTTCCACCGCTTGCCGCAACTTAACGAGACGCGGGCCTAGCATTTCAGCCGACGATGCCGACGGGGGCAGCGACGGCCAGTCCTCCGCCCGCCTCCCAACCCTTCCCCAATTCGGGAAGGGCGTGGGAGGAGAGCGACGCATAGCGTGACGCCCAGGCAGGTGTGCCCTCGACCGGGTGGTCTCGGGCGCAACTTGCGTTCAAAGACTCGATGATTCACGGGATTCTGCAATTCACATCAAGTATCGCATTTCGCTACGTTCTTCATCGTTGCGGGAGCCAAGATATCCGTTGCCGAGAGTCGTTTAGATAGATAGAGGCAAAGGTCACCCCCCATTGCCATGGGTGCGGGCCGATGCTCTTTGTTGGTATTGTCCTTGACGCCTGTGGCGCCGTTTTGCTTGTTACGGCCGCAACGACATCCCCACCACGCGTAACCCGACCGGGACCCGAGGGTCCTAGATCAAGGCGGCGAGGGGAGGGGAGCGCAACGACCGGTCGGGGGACGCCCACCCGGGGGCGGGCAATCCGACGACGAGTGATGATAACGTGTTCGCGGGTGTGTCGATCAGGATTGGCAATGATCCTTCCGCAGGTTCACCTACGGAAACCT

>QYBSZ_1

TCGCTAGGCTGGAGGCATTGCCCCCTCAGAGTTCCTCTTGCACCGGGGCATCGCATGGTTGGACGGCTAAAGGTGTTCCACCGCTTGCCGCAACTTAACGAGACGCGGGCCTAGCATTTCAGCCGACGATGCCGACGGGGGCAGCGACGGCCAGTCCTCCGCCCGCCTCCCAACCCTTCCCCAATTCGGGAAGGGCGTGGGAGGAGAGCGACGCATAGCGTGACGCCCAGGCAGGTGTGCCCTCGACCGGGTGGTCTCGGGCGCAACTTGCGTTCAAAGACTCGATGATTCACGGGATTCTGCAATTCACATCAAGTATCGCATTTCGCTACGTTCTTCATCGTTGCGGGAGCCAAGATATCCGTTGCCGAGAGTCGTTTAGATAGATAGAGGCAAAGGTCACCCCCCATTGCCATGGGTGCGGGCCGATGCTCTTTGTTGGTATTGTCCTTGACGCCTGTGGCGCCGTTTTGCTTGTTACGGCCGCAACGACATCCCCACCACGCGTAACCCGACCGGGACCCGAGGGTCCTAGATCAAGGCGGCGAGGGGAGGGGAGCGCAACGACCGGTCGGGGGACGCCCACCCGGGGGCGGGCAATCCGACGACGAGTGATGATAACGTGTTCGCGGGTGTGTCGATCAGGATTGGCAATGATCCTTCCGCAGGTTCACCTACGGAAACCT

>YNTC_1

TCGCTAGGCTGGAGGCATTGCCCCCTCAGAGTTCCTCTTGCACCGGGGCATCGCATGGTTGGACGGCTAAAGGTGTTCCACCGCTTGCCGCAACTTAACGAGACGCGGGCCTAGCATTTCAGCCGACGATGCCGACGGGGGCAGCGACGGCCAGTCCTCCGCCCGCCTCCCAACCCTTCCCCAATTCGGGAAGGGCGTGGGAGGAGAGCGACGCATAGCGTGACGCCCAGGCAGGTGTGCCCTCGACCGGGTGGTCTCGGGCGCAACTTGCGTTCAAAGACTCGATGATTCACGGGATTCTGCAATTCACATCAAGTATCGCATTTCGCTACGTTCTTCATCGTTGCGGGAGCCAAGATATCCGTTGCCGAGAGTCGTTTAGATAGATAGAGGCAAAGGTCACCCCCCATTGCCATGGGTGCGGGCCGATGCTCTTTGTTGGTATTGTCCTTGACGCCTGTGGCGCCGTTTTGCTTGTTACGGCCGCAACGACATCCCCACCACGCGTAACCCGACCGGGACCCGAGGGTCCTAGATCAAGGCGGCGAGGGGAGGGGAGCGCAACGACCGGTCGGGGGACGCCCACCCGGGGGCGGGCAATCCGACGACGAGTGATGATAACGTGTTCGCGGGTGTGTCGATCAGGATTGGCAATGATCCTTCCGCAGGTTCACCTACGGAAACCT

**References:**

**Shaw J, Lickey EB, Beck JT, Farmer SR, Liu WS, Miller J, Siripun KC, Winder CT, Schilling EE, Samll RR.** **2016.** The tortoise and the hare II : Relative utility of 21 noncoding chloroplast DNA sequences for phylogenetic analysis. *American Journal of Botany* **92**(1): 142-166 DOI: 10.3732/ajb.92.1.142

**Shaw J, Lickey EB, Schilling EE, Samll RR.** **2007.** Comparison of whole chloroplast genome sequences to choose noncoding regions for phylogenetic studies in angiosperms : The Tortoise and the hare III. *American Journal of Botany* **94**(3): 275–288 DOI:10.3732/ajb.94.3.275.

**Chen JM, Du ZY, Long ZC, Gichia AW, Wang QF. 2017.** Molecular divergence among varieties of *Ottelia acuminata* (Hydrocharitaceae) in the Yunnan-Guizhou Plateau. *Aquatic Botany* **140**: 62-68 DOI: 10.1016/j.aquabot.2017.03.001
